# Studies on a Sulfoxide-Bridged Tn Antigen Mimetic: Interaction with Macrophage Galactose Lectin and Inhibition of Sialyltransferase ST6GALNAC1

**DOI:** 10.1021/acsomega.6c00385

**Published:** 2026-04-11

**Authors:** Andrea Sodini, Emanuele Casali, Maria Alejandra Travecedo, Filipa Marcelo, Fabrizio Chiodo, Filippo Rambaldi, Sandra J. van Vliet, Fabio Dall’Olio, Cristina Nativi

**Affiliations:** † Department of Chemistry, DICUS, 9300 University of Florence, via della Lastruccia 3-13, Sesto Fiorentino 50019, Italy; ‡ Department of Chemistry, 19001 University of Pavia, via Taramelli 10, Pavia 27100, Italy; § UCIBIO − Applied Molecular Biosciences Unit, Department of Chemistry, NOVA School of Science and Technology, 119482 NOVA University Lisbon, Caparica 2829-516, Portugal; ∥ Associate Laboratory i4HB − Institute for Health and Bioeconomy, NOVA School of Science and Technology, NOVA University Lisbon, Caparica 2829-516, Portugal; ⊥ Bio-Organic Chemistry Unit, 9327 Institute of Biomolecular Chemistry CNR, via Campi Flegrei 34, Napoli 80078, Italy; # Department of Molecular Cell Biology and Immunology Amsterdam UMC, 1209 Vrije Universiteit Amsterdam, Amsterdam 1081HV, The Netherlands; ∇ Amsterdam Institute for Immunology and Infectious Diseases, Cancer Immunology, Amsterdam 1081 HV, The Netherlands; ○ Department of Medical and Surgical Sciences, DIMEC, University of Bologna, via San Giacomo 14, Bologna 40126, Italy

## Abstract

Aberrant O-glycosylation is a defining hallmark of epithelial cancers, where truncated mucin-type glycans such as Tn and sialyl-Tn (sTn) are prominently displayed. Despite their tumor specificity, these tumor-associated carbohydrate antigens (TACAs) elicit only weak immune responses, limiting their impact in vaccine-based immunotherapy. Growing evidence implicates two major factors in this poor immunogenicity: the intrinsic engagement of Tn/sTn with the immunosuppressive macrophage galactose-type lectin (MGL) on antigen-presenting cells and the “self” nature of Tn/sTn mucin carriers such as MUC1. Both processes critically depend on the *N*-acetyl functionalities of the Tn determinant. We previously developed a stable Tn mimetic, 2-deoxy-2-thio-α-O-galactoside (compound **1**), which lacks the NHAc group and exhibits notable immunostimulatory properties in vivo. In this study, we provide new structural insights into the role of the NHAc moiety in the mimetic **1** presentation and its interaction with MGL, thereby advancing the design principles for next-generation Tn analogues with improved immunological behavior. An additional focus is on the pathogenic upregulation of sTn in tumors, primarily driven by overexpression of the sialyltransferase ST6GALNAC1. We demonstrate that mimetic **1** and its oxidized analogue **2** act as inhibitors of ST6GALNAC1—representing, to the best of our knowledge, the first reported monosaccharide inhibitors of this enzyme. Although the inhibitory potency is modest, these compounds establish a valuable chemical starting point for targeting cancer-associated sialylation, an effort currently constrained by the lack of structural information for ST6GALNAC1.

## Graphical abstract

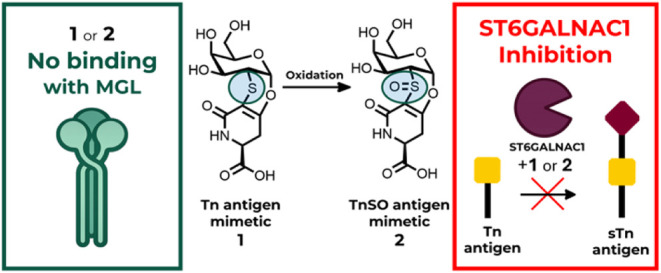

## Introduction

Altered glycosylation can be considered a hallmark of cancer.^1^ Compared with their normal tissue counterparts, tumors display a wide array of tumor-associated glycans, also known as tumor-associated carbohydrate antigens (TACAs). Among the most relevant TACAs are those derived from the incomplete synthesis of mucin-type O-linked chains. This type of O-glycosylation involves the initial addition of an *N*-acetylgalactosamine (GalNAc) residue to serine or threonine. This reaction, which is catalyzed by the large family of glycosyltransferases called polypeptide *N*-acetylgalactosaminyl transferases (GalNAc-Ts), differing in subtle substrate specificity, results in the biosynthesis of the Tn antigen (GalNAcα1-O-Ser/Thr) (Figure 1).^2^ The successive addition of a sialic acid residue to GalNAc, mediated mainly by sialyltransferase ST6GALNAC1, results in the biosynthesis of the sialyl-Tn (sTn) antigen. Addition of Gal to GalNAc and of sialic acid to Gal produces the Thomsen–Friedenreich (TF) and the sialyl-TF antigens (sTF), respectively (Figure 1). In normal tissues, the biosynthesis of O-linked chains proceeds further toward more complex structures, but in cancer a strong tendency to accumulation of truncated structures, like Tn and sTn antigens, is observed.

**Figure 1:**
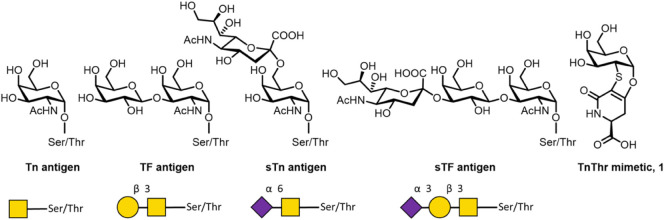
Structure of MUC1 Tn, TF, sTn, and sTF antigens and of Tn-Thr mimetic, **1**.

TACAs are exposed to the immune system;^3,4^ however, because of their intrinsic poor immunogenicity and tumor escape mechanisms, they are ineffective at eliciting a protective immune response.^5^ In fact, the Tn and sTn determinants (Figure 1), frequently selected as antigens in cancer vaccine development, have afforded only modest results.

The abnormal O-glycan Tn is structurally characterized by an alpha-glycosidic linkage between the GalNAc moiety and serine (Ser) or threonine (Thr) residues in glycoproteins. Interestingly, the attachment of GalNAc to either Thr or Ser results in conformational differences in Tn protein carriers such as mucin 1 (MUC1),^6–9^ affecting the shape of the peptide backbone and influencing its antigenic properties (see Figure S10). In particular, an interaction between the peptide backbone and the GalNAc O-linked to a Thr unit was highlighted^10^ and attributed to the hydrogen bond between the NH proton of the Thr and the carbonyl group of the sugar.^8^ This hydrogen bond locks the orientation of the pyranose ring relative to the peptide backbone and is crucial for its presentation in tumor microenvironments.^11,12^ The Tn-Thr antigen is indeed more immunogenic than Tn-Ser, but still “too self” and mainly tolerated by the immune system.

The Tn antigen and its sialylated form sTn (Figure 1) are coexpressed on tumor cells and recognized by macrophage galactose-type lectin (MGL) on antigen-presenting cells (APCs) such as macrophages and dendritic cells (DCs).^13,14^ In its native trimeric form, MGL acts as a receptor for short cancer-associated O-glycans (Tn, sTn, and TF). Binding of MGL to MUC1 Tn/sTn, CD43, and CD45 triggers immunosuppressive effects, including IL-10 production and effector T-cell apoptosis.^15^ The concomitant engagement of MGL with MUC1 Tn/sTn, CD43, and CD45 activates immunosuppressive mechanisms, including IL-10 production and effector T-cell apoptosis.^16–18^

The crystal structure of MGL complexed with Tn reveals key interactions involving the acetamide group (NHAc), which account for the markedly higher affinity of MGL for GalNAc relative to Gal.^19^

Taken together, the interaction of Tn/sTn with the immunosuppressive MGL receptor on APCs, along with the intrinsic “self” nature of MUC1 and other Tn-bearing carriers, very likely contributes to the notably weak immunogenicity of these TACAs.

Building on this, analogues of Tn and sTn antigens have been developed for MUC1-based cancer vaccines to prevent antigen escape and overcome tumor-induced TACA immunotolerance.^20^

In particular, a conjugate vaccine containing four copies of the stable 2-deoxy-2-thio-α-O-galactoside **1** (see Figure 1) as a mimetic of the Tn-Thr determinant, linked to the protein carrier CRM197, induced a strong T-cell-dependent immune response against triple-negative breast cancer in mice.^21^ The Tn-Thr mimetic **1** is structurally locked, presents an α-O-glycosidic linkage, and retains the native Tn^4^C_1_ chair conformation but lacks the NHAc residue. The bioactivity of **1** was confirmed by saturation-transfer-difference (STD) NMR spectroscopy by using a galactoside-specific model lectin,^22^ but to MGL it binds even more weakly than Gal (millimolar affinity).^23^

To reproduce the multiple copies of Tn carried on MUC1, engineered nanoparticles,^24^ niosomes,^25^ peptide nanofibers,^26^ or outer membrane vesicles^27^ were glycosylated to display multiple residues of **1**. The glyco-constructs so obtained exhibited strong in vitro immunogenic properties^24–26^ and the ability to elicit in vivo high titers of specific antibodies, along with remarkable efficacy in a mouse model of aggressive triple-negative breast cancer (TNBC).^21,27^ Altogether, these data indicate that the locked structure featuring the mimetic **1** and the absence of the NHAc (accounting for the lack of interaction with the immunosuppressive MGL receptor) provide a strong contribution to break the tolerance toward the native Tn antigen and make **1** an effective nonpeptide Tn mimetic.

Building on the advances reported for mimetic **1**, we sought to investigate whether replacing the sp^2^ oxygen of the NHAc C=O group with an S=O functionality could promote the formation of a novel binding network with native ligands. To this end, we report the synthesis and conformational studies of sulfoxide **2,** as well as the binding interactions with MGL.

An important aspect of cancer-associated mucin biology is the relationship between Tn and sTn antigens. The sTn mucin-type antigen (Neu5Acα2–6GalNAc-O-Ser/Thr; Figure 1) is rarely detected in normal tissues but is highly expressed in most adenocarcinomas, including gastric, colorectal, ovarian, breast, and pancreatic cancers.^28^ sTn expression is linked to greater tumor aggressiveness and poor prognosis in several carcinomas; it appears in the sera of patients with gastric, colorectal, and ovarian cancers. Its presence in premalignant gastrointestinal lesions also suggests a role in the early tumorigenesis. In cancer, sTn is upregulated mostly because of the overexpression of sialyltransferase ST6GALNAC1.^29^

Given the potential in vivo applicability of mimetic **1**, assessing its ability to modulate the Tn/sTn balance through the inhibition of ST6GALNAC1 is particularly relevant. Accordingly, we examined the capacity of mimetics **1** and **2** to inhibit the ST6GALNAC1 activity.

## Results and Discussion

### Synthesis of Sulfoxide **2**

The Tn-Thr mimetic **1** ^22^ was oxidized by treatment with *meta*-chloroperbenzoic acid (*m*CPBA) in dichloromethane as the solvent at 0 °C. After 16 h, the organic solvent was evaporated, and the crude was purified by column chromatography on silica gel to afford the sulfoxide **2** (69% yield, rt, 16 h) as a single diastereoisomer and as an amorphous solid (Scheme 1). The tentative oxidation of **2** to afford the corresponding sulfone^30^ failed (see Supporting Information), even under harsh conditions (excess of oxidant, by heating up to 80 °C). To assign the configuration of the chiral sulfur, ^1^H NMR spectra were recorded, but no unambiguous signal shifts were detected (see Figures S8 and S9 and Table S1).

**Scheme 1:**
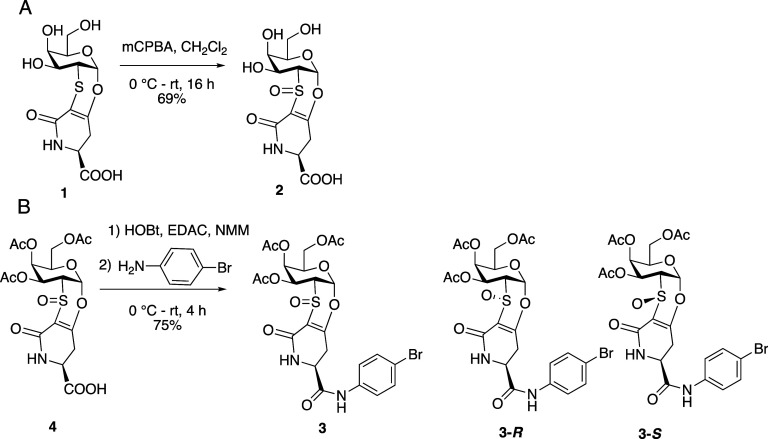
Panel A: Synthesis of Sulfoxides **2.** Panel B: Synthesis of Derivative **3** and Structure of Diastereoisomers **3-** *R* and **3-** *S*

### X-ray Structure of Sulfoxide Derivative **3**

To undoubtedly define the stereochemistry of compound **2**’s stereogenic sulfur, X-ray analysis was necessary. Unfortunately, although different solvents/mixtures of solvents were screened to crystallize sulfoxide **2**, only needle-like crystals were obtained, which were not suitable for X-ray diffraction. Thus, we synthesized the bromo derivative **3** from peracetylated sulfoxide **4** (see Supporting Information for details), by treatment with 1-hydroxybenzotriazole (HOBt), 1-ethyl-3-(3-(dimethylamino)­propyl)­carbodiimide (EDAC), and *N*-methyl morpholine (NMM) in dimethylformamide as the solvent (see Scheme 1). After 10 min of stirring at 0 °C, *para*-bromoaniline was added. The reaction was completed in 4 h, after purification by chromatography on silica gel, affording the bromosulfoxide **3** (75%, see Scheme 1) as a white foam. Upon crystallization from dichloromethane/methanol (1:1), yellow crystals of **3**, suitable for X-ray analysis, were obtained (Figure 2).

**Figure 2:**
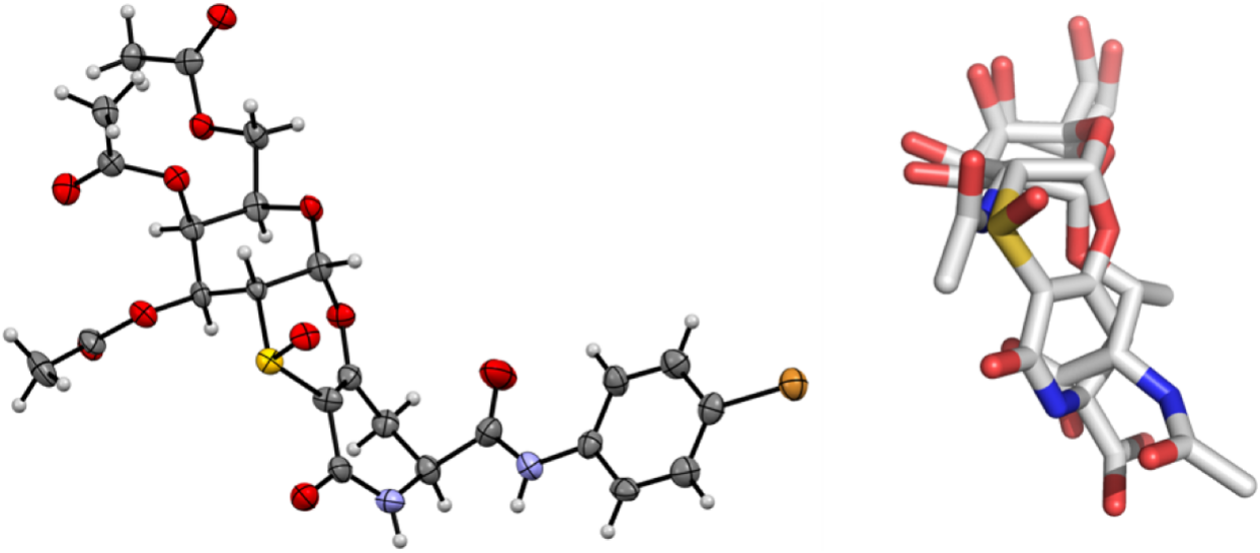
Left: Crystal structures of compound **3**-*S*. Color code: red, oxygen; light blue, nitrogen; gray, carbon; yellow, sulfur; light orange, bromine. Solvent molecules are omitted for clarity (image elaborated with Mercury). Right: Superimposed structures of native Tn and Tn mimetic sulfoxide **2**-*S*.

The X-ray structure of derivative **3** allowed to assign the *S* stereochemistry of the stereogenic sulfur of **2** and highlighted the spatial orientation of the S=O residue, which points far from the pyranose ring. To further clarify the origin of the preferential oxidation at the sulfur atom leading to **2**-*S*, we investigated the two possible oxidation pathways of **1** with *m*CPBA=those yielding **2**-*S* or **2**-*R*=using DFT calculations (see the SI for additional computational details). Analysis of the reaction free-energy profile showed that the reaction occurs through a single-step process in each direction, showing the pathway leading to the experimentally observed **2**-*S* isomer, with an activation barrier nearly 4 kcal/mol lower than that of the pathway leading to **2**-*R* (see Figure 3).

**Figure 3:**
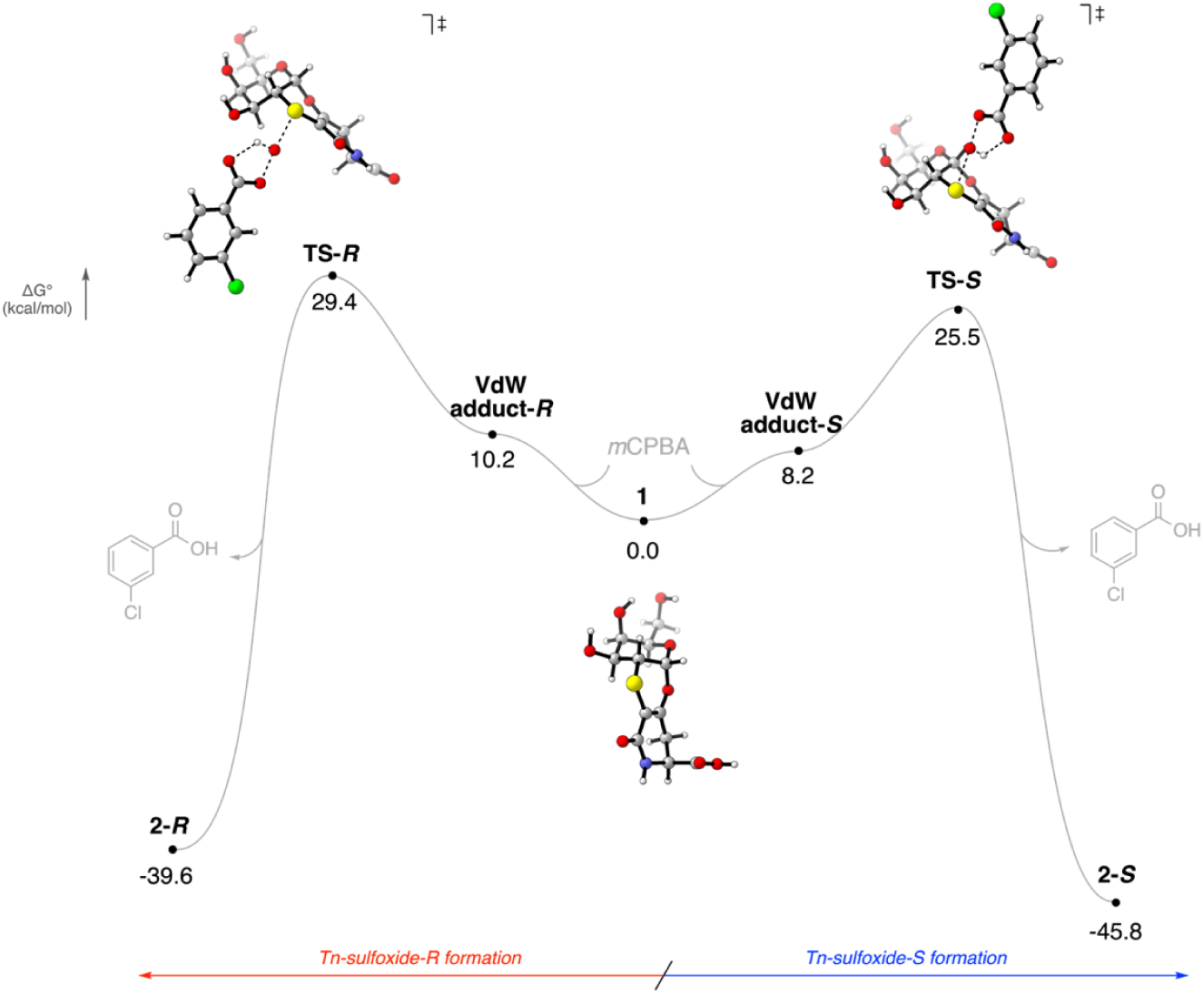
Full Gibbs free-energy reaction profile comparing the two possible reaction pathways leading to **2**-*S* and **2**-*R*. The energies reported were obtained by using DFT at the PCM­(H_2_O)-GD3-M06–2X/def2TZVP//PCM­(H_2_O)-B3LYP/6–31g­(d) calculation level.

A closer inspection of the transition-state structures showed that the *m*CPBA approach in **TS**-*S* is less sterically hindered compared with **TS**-*R*. In addition, the three hydroxyl groups of the glycoside moiety remain engaged in a three-center hydrogen-bond network, which limits their ability to participate in secondary interactions that could otherwise facilitate the approach toward the pro-*R* face. This result is fully consistent with the experimental observations and can be attributed to the different steric accessibilities of the two faces of the thioxane moiety. The reduced steric hindrance on the convex face of the thioxane ring, relative to the more crowded concave face, facilitates the approach of *m*CPBA and leads to a lower-energy oxidation pathway. Moreover, the relative stability of the two final products mirrors that of their corresponding transition states: **2**-*S* is almost 6 kcal/mol more stable than **2**-*R*, indicating that **2**-*S* is not only the kinetically favored product but also the thermodynamically more stable one.

### NMR Studies of Sulfoxide **2** and Trivalent Sulfoxide **5** vs MGL and ELISA Tests

The binding properties of sulfoxide **2** against MGL were evaluated by NMR spectroscopy. Moreover, since the Tn density and presentation modulate recognition, the trivalent sulfoxide **5** was also synthesized for MGL binding studies. Derivative **5** was prepared from acetyl derivative **6** (see Scheme 2) by reaction with tris­(2-aminoethyl)­amine (TREN) in the presence of NMM, HOBt, and EDAC in dimethylformamide as the solvent. After 16 h, the crude compound was purified by chromatography on silica gel to afford the trivalent derivative **7** (73%) (Scheme 2). The acetyl protecting groups were removed with ammonia in methanol (2M, 22 h, rt) to afford, upon precipitation from CH_3_OH, the derivative **8** (85%). This latter was dissolved in DMF, cooled to 0 °C, and treated with *m*CPBA. The mixture was stirred at rt until the complete conversion of the starting material (by TLC and MS). The solvent was then removed under vacuum, and the crude product was repeatedly washed with CH_2_Cl_2_ to afford the trivalent sulfoxide **5** (79%) (Scheme 2).

**Scheme 2:**
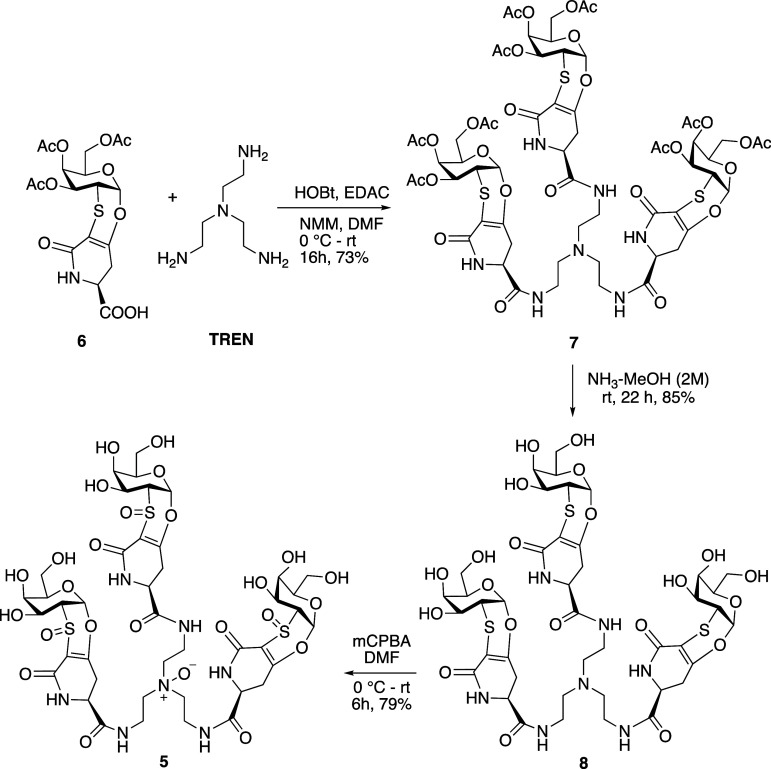
Synthesis of Trivalent Sulfoxide **5**

Binding studies of the monovalent sulfoxide **2** and the trivalent sulfoxide **5** against the uniformly ^15^N-labeled carbohydrate recognition domain of MGL (MGL-CRD) were performed in solution by NMR. The recombinant MGL-CRD was expressed and purified for this purpose, as previously described.^31^ ^1^H–^15^N HSQC-based titration of the ^15^N-MGL-CRD at a fixed concentration (200 μM) in the presence of increasing concentrations of either **2** or **5** was recorded (Figures S4 and S5).

Chemical shift perturbations (CSPs) were monitored by comparing the variations in ^1^H and ^15^N chemical shifts between the apo form of MGL-CRD and the complexes formed with **2** or **5** (see Supporting Information for details). The histograms of the associated combined CSP (Δδ_comb_) are shown for sulfoxide **2** (Figure 4A) and for the trivalent sulfoxide derivative **5** (Figure 4B). In detail, the amide cross-peaks of Y236, Q267, D269, W271, E280, C296, N292, and the indole NH of W271 residues are moderately perturbed in the presence of **2** or **5** (Δδ_comb_ > 0.05 ppm). The CSP patterns observed upon addition of **2** or **5** are consistent with those detected when MGL-CRD binds to native Tn.^15,31^ The ^1^H, ^15^N-HSQC titration further shows that ligands **2** and **5** bind more weakly than the Tn antigen, as evidenced by the fast exchange/intermediate regime on the NMR chemical shift time scale and the need for high ligand concentrations to reach saturation (Figures S4 and S5).

**Figure 4:**
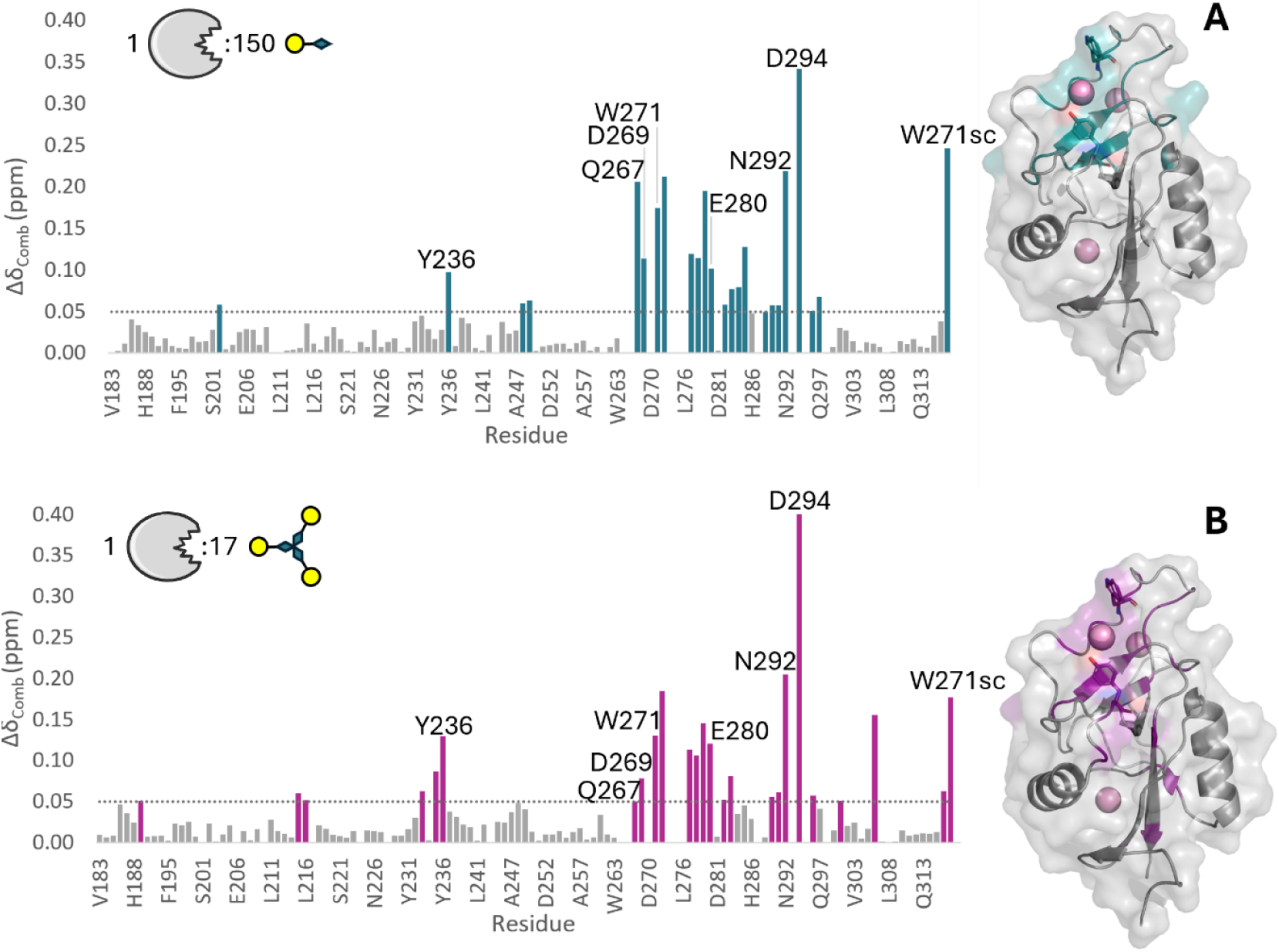
Molecular recognition of compounds **2** and **5** by MGL-CRD. Histogram of the ^1^H, ^15^N-associated combined chemical shift (Δδ_comb_) for each amino acid of MGL-CRD/**2** complex in the presence of 150 equiv of monovalent **2** (panel A) and of MGL-CRD/**5** complex in the presence of 17 equiv of trivalent **5** (panel B). The most perturbed residues (Δδ_comb_ > 0.05 ppm) are highlighted in blue and purple for the monovalent **2** and trivalent **5**, respectively. In both panels, these residues were mapped in the X-ray crystallography structure of the MGL-CRD/GalNAc complex (PDB code: 6PY1). In sticks are displayed the key MGL residues (Y236 and W271). The calcium ions are represented as purple spheres.

The apparent dissociation constants (*K*_Dapp_) were qualitatively deduced by ^1^H–^15^N HSQC-based titration. The data suggest that the monomer **2** binds in the high millimolar range (*K*_Dapp_ ≈ 2.20 ± 0.75 mM), whereas the trimer **5** displays an improved affinity (*K*_Dapp_ ≈ 0.46 ± 0.08 mM) (see Supporting Information for details). While our measurements are qualitative, the stronger apparent binding observed for ligand **5** is consistent with the possibility that, in solution, this ligand can simultaneously engage multiple MGL-CRDs. However, a different behavior is expected at the cellular level, as the trimeric organization of MGL on the cell surface constrains each CRD to a fixed orientation and defined spatial separation. Our findings highlight that both the monovalent and the trivalent sulfoxides interact with the same binding site of the native Tn antigen, although with a clear lower affinity.

To confirm the lack of binding between sulfoxide **2** and MGL, a solid-phase assay, ELISA-based, was also performed (Figure S3). Two multivalent Tn-sulfoxide (TnSO) BSA conjugates, namely TnSO­[BSA]_19_ and TnSO­[BSA]_24_, were prepared as previously reported^21^ and screened against MGL by coating them on an ELISA plate. Also, in the ELISA experimental conditions, TnSO presented in a multivalent manner on BSA showed no binding or very weak binding to MGL compared to the positive control used (polyacrylamide polymers coated with Tn antigen).

### ST6GALNAC1 Inhibition

Aberrant sialylation is closely linked to higher cancer aggressiveness and worse patient outcomes. For example, the sTn antigen helps cancer cells leave the primary tumor, invade surrounding tissue, and enter lymphatic vessels.^32^ The sTn increase is mainly caused by cancer-driven overexpression of the enzyme ST6GALNAC1, which adds a sialic acid to the Tn antigen.^33^ Because sialyltransferases (STs) play an important role in tumor progression, blocking them has become a promising strategy for developing new antimetastatic cancer treatments.

A large number of sialyltransferase inhibitors have been developed.^34^ Basically, these inhibitors can be grouped into three categories: i) mimics of the donor substrate CMP-sialic acid (CMP-Sia); ii) mimics of the acceptor substrates; and iii) other molecules. Inhibitors of the first group exhibit low *K*_i_ values in the order of μM but poor specificity toward individual sialyltransferase enzymes because they target the common acceptor substrate CMP-Sia. To the second group belong the methyl 5a′-carbadisaccharides, which have been found to inhibit ST6GAL1 and ST3GAL1 with *K*_i_ values ranging between 0.2 and 0.9 mM. Also, fluorinated compounds derived from mucin-type oligosaccharides^35^ have been found to display a modest *K*_i_ (1.9 mM) for ST6GAL1, but no activity on ST3GAL1. At present, there are no synthetic compounds specifically aimed at the inhibition of the six ST6GALNAC enzymes (ST6GALNAC1 to ST6GALNAC6). Of these enzymes, only ST6GALNAC1 and, to a lesser extent, ST6GALNAC2, can synthesize the sTn antigen.

Thus, the inhibitory activity of compounds **1** and **2** on ST6GALNA1 was assessed by using an in vitro assay based on the recombinant ST6GALNAC1-mediated enzymatic transfer of radiolabeled sialic acid from CMP-[^3^H] sialic acid to a saturating concentration of asialo bovine submaxillary mucin (aBSM). After incubation, the acid-insoluble radioactivity (i.e., radioactivity incorporated into aBSM) was collected and counted (see Supporting Information for details). After subtraction of blank incorporation (without enzyme), this method provides an accurate, sensitive, and relatively affordable routine method to measure sialyltransferase. The assay used several concentrations of compounds **1** and **2**, which differ only in their oxidized or reduced status (Figure 5A).

**Figure 5:**
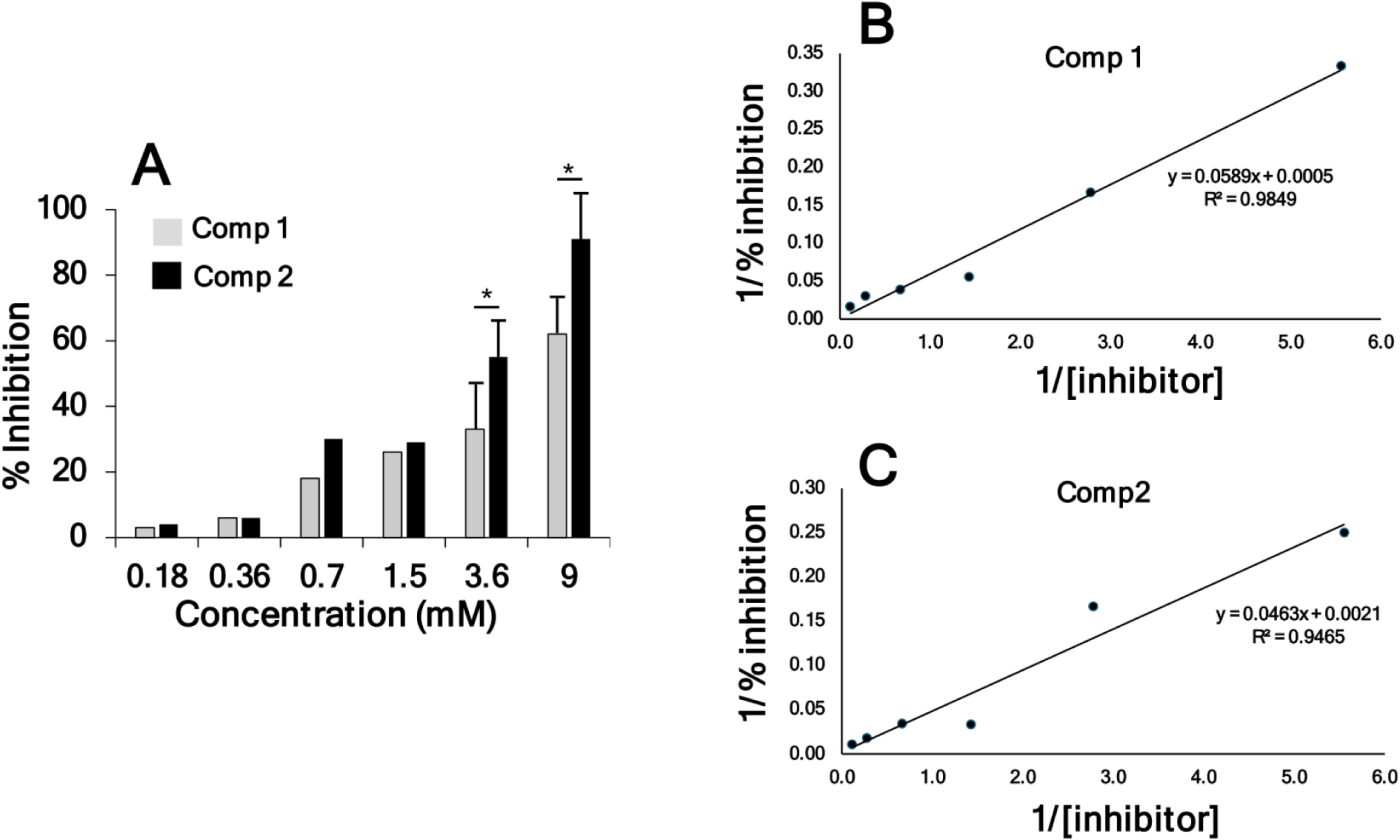
Inhibition of the ST6GALNAC1 enzyme activity in vitro by Tn-Thr mimetic **1** and the corresponding sulfoxide **2**. (A) Data are the mean ± SD of at least 3 independent determinations. * *p* ≤ 0.05 according to Student’s *t*-test. B and C: Data from panel A are presented as double reciprocal plots for compounds **1** (B) and **2** (C). The IC_50_ values calculated from the reported equations were 3 mM and 2.4 mM for compounds **1** and **2**, respectively.

The calculated IC_50_ concentrations of compounds **1** and **2** were 3 mM and 2.4 mM, respectively (Figure 5B and C). Although both *K*_i_ and IC_50_ reflect the inhibitory potential of the drug, they are not the same and cannot be directly compared. We have calculated the approximate IC_50_ in our experimental conditions (i.e., with a single concentration of donor and acceptor substrates, varying only the inhibitor concentration). *K*_i_ determination would require a much more complex investigation, which goes beyond the purpose of the present work. As reported,^36^ we have calculated an approximate concentration of native ST6GALNAC1 acceptor sites (i.e., terminal GalNAc residues) in aBSM from our assay (see SI for details), finding a value of 1.2 mM. A similar concentration of our inhibitors results in an approximate 25% inhibition of incorporation on aBSM. If the affinity of native Tn and our inhibitors for ST6GALNAC1 were the same, a 50% inhibition would be expected. This indicates that the affinity of the two inhibitors for ST6GALNAC1 is approximately half that of the native Tn carried by aBSM. Although both compounds show only modest affinity for ST6GALNAC1—insufficient at this stage to support their use in vivo—they nonetheless represent the first synthetic inhibitors ever reported for this enzyme. Their discovery provides an essential starting point for the development of analogues with enhanced potency. Indeed, the versatile structure of **1** and **2** will allow for the introduction of simple aliphatic or aromatic rings as well as a sialic acid moiety linked at C-3 or C-6.

In this rapidly evolving field, the identification of initial hit compounds is particularly valuable, given the current lack of a crystal structure for ST6GALNAC1. Once this structure is determined, it will be possible to advance these hit compounds and design more effective inhibitors.

## Conclusion

Because it lacks an NHAc group, compound **1** is a unique Tn-Thr mimetic. Its carbohydrate part stays in a fixed position and, unlike the native antigen, does not rely on hydrogen bonds or water bridges with underlying peptides to assume an immunogenic presentation able to overcome tumor-induced tolerance.^8^ In fact, when multiple copies of mimetic **1** were attached to structurally distinct scaffolds, such as cyclic peptides,^37^ liposomes,^25^ nanomaterials,^24,26,38^ proteins,^21^ or outer membrane vesicles,^27^ the resulting constructs triggered strong immune responses and the production of specific anti-Tn antibodies in TNBC animal models. The absence of the NHAc group very likely accounts for the lack of interactions between **1** and immunomodulating MGL lectin. To evaluate the impact of removing the NHAc group, we substituted the C=O moiety of native Tn with a sulfoxide (S=O).

Relying on the sulfur-bridged scaffold of mimetic **1**, we synthesized its sulfoxide derivative **2**, thereby introducing the S=O functionality. Molecular calculations, fully consistent with the X-ray structure (Figure 3), indicate that the **2**-*S* stereoisomer is preferred, as it results from the lower steric hindrance encountered by *m*CPBA approaching the concave face of the thioxane ring. In this isomer, however, the sp^2^ oxygen is oriented away from the pyranose ring and occupies a spatial position distinct from that of the C=O group in the native Tn antigen (see Figure 2, right panel). Thus, it is not surprising that both monovalent sulfoxide **2**-*S* and trivalent sulfoxide **5** exhibited only negligible binding to MGL.

In this study, we also demonstrated that, in addition to their well-known role as immunogens, the Tn-Thr mimetic **1** and the corresponding sulfoxide **2** are, based on current knowledge, the only inhibitors of the sialyltransferase ST6GALNAC1, the enzyme responsible for generating the cancer-associated sTn antigen. Although their inhibitory activity is modest, it provides a valuable starting point for structural optimization, which is currently hindered by the lack of an ST6GALNAC1 crystal structure.

Concluding, i) the different accessibility of one of the two faces of the thioxane ring confirms the structural rigidity of the mimetic **1,** which well explains the higher immunogenicity compared to the native Tn; ii) the lack of the NHAc residue is confirmed to be directly related to the poor binding interactions of **1** and **2** with immunosuppressive MGL; iii) the mimetic **1** emerges as a versatile molecule endowed with multiple potential biological functions relevant to cancer control. Collectively, our findings shed light on fundamental aspects of Tn/MGL recognition, expand the repertoire of Tn antigen analogues, and identify pioneering leads for sialyltransferase inhibition. These advances provide new opportunities for the development of more effective MUC1-based cancer vaccines and therapeutic strategies aimed at modulating aberrant glycosylation in cancer.

## Experimental Section

Complete crystallographic data of compound **3**, in CIF format, have been deposited with the Cambridge Crystallographic Data Centre. Data can be obtained free of charge from the Cambridge Crystallographic Data Centre via www.ccdc.cam.ac.uk/data_request/cif.

### NMR Binding Studies

All the NMR experiments were acquired on a Bruker Avance III 600 MHz spectrometer equipped with a 5 mm inverse detection triple-resonance cryogenic probe head with z-gradients in 3 mm NMR tubes at 293 K. Recombinant MGL-CRD was uniformly ^15^N-labeled and prepared in buffer with 20 mM Ca^2+^ (10 mM Tris buffer (pH 7.4) containing 75 mM NaCl and 20 mM CaCl_2_) as described.^16^ ^1^H–^15^N HSQC spectra of MGL-CRD (200 μM) were obtained following titration with increasing concentrations of the monovalent compound **2,** up to a 1:150 protein-to-ligand molar ratio (Figure S4), or with the trivalent compound **5** at a 1:17 protein-to-ligand molar ratio (Figure S5).

### Inhibition of ST6GALNAC1

Asialo bovine submaxillary mucin (aBSM) to be used as the ST6GALNAC1 acceptor substrate was obtained by subjecting bovine submaxillary mucin (Sigma) to mild acid hydrolysis (50 mM H_2_SO_4_, 80 °C, 1 h), followed by extensive dialysis against water and drying. The ST6GALNAC1 assay mixture contained a final volume of 50 μL:25 mM Tris/HCl buffer, pH 7.5, 10 mM MnCl_2_, 1.2 × 10^4^ Bq CMP-[^3^H]­Sialic acid (0.55 μM) (American Radiolabeled Chemicals), 300 μg aBSM, 0.15 μg recombinant ST6GALNAC1 (R&D Systems), and variable amounts of inhibitors (from 0 to 9 mM). Samples were incubated for 2 h at 37 °C. Samples without enzyme were incubated in parallel as blanks. The acid-insoluble radioactivity incorporated into aBSM was measured after precipitation with 1% phosphotungstic acid in 0.5 M HCl, followed by three washings with 1% phosphotungstic acid in 0.5 M HCl. Samples were then solubilized by boiling in 0.5 M HCl, transferred into liquid scintillation vials, and, after the addition of 3 mL of liquid scintillation cocktail (Beckman Coulter), counted in a liquid scintillation counter. The incorporation of the samples without enzyme was subtracted. The IC_50_ concentration of the two compounds was calculated as reported (see Supporting Information).

## Supplementary Material



## Notes

Computational data, X-ray analysis, ELISA tests, NMR binding studies, estimation of dissociation constants by NMR, inhibition of ST6GALNAC1, synthesis and NMR spectra of compounds **2**–**9**, **11**, TnSO­[BSA]_24_, and TnSO­[BSA]_19_ (PDF)

## ◆

CIC bioGUNE, Basque Research and Technology Alliance, Bizkaia Technology Park, Ed. 800. E-48160, Derio, Spain

## References

[1] Mereiter S., Balmaña M., Campos D., Gomes J., Reis C. A. (2019). Review
Glycosylation in the Era of Cancer-Targeted Therapy: Where Are We
Heading?. Cancer Cell.

[2] Gill D. J., Clausen H., Bard F. (2011). Location, Location,
Location: New
Insights into O -GalNAc Protein Glycosylation. Trends Cell Biol..

[3] Hanisch F. G., Müller S. (2000). MUC1: The Polymorphic Appearance
of a Human Mucin. Glycobiology.

[4] Karsten U., Serttas N., Paulsen H., Danielczyk A., Goletz S. (2004). Binding Patterns of DTR-Specific
Antibodies Reveal
a Glycosylation-Conditioned Tumor-Specific Epitope of the Epithelial
Mucin (MUC1). Glycobiology.

[5] Dobrochaeva K., Khasbiullina N., Shilova N., Antipova N., Obukhova P., Ovchinnikova T., Galanina O., Blixt O., Kunz H., Filatov A. (2020). Specificity of Human
Natural Antibodies Referred to
as Anti-Tn. Mol. Immunol..

[6] Mazal D., Lo-Man R., Bay S., Pritsch O., Dériaud E., Ganneau C., Medeiros A., Ubillos L., Obal G., Berois N., Bollati-Fogolin M., Leclerc C., Osinaga E. (2013). Monoclonal
Antibodies toward Different Tn-Amino Acid Backbones Display Distinct
Recognition Patterns on Human Cancer Cells. Implications for Effective
Immuno-Targeting of Cancer. Cancer Immunol.,
Immunother..

[7] Coelho H., Matsushita T., Artigas G., Hinou H., Cañada F. J., Lo-Man R., Leclerc C., Cabrita E. J., Jiménez-Barbero J., Nishimura S. I., Garcia-Martín F., Marcelo F. (2015). The Quest
for Anticancer Vaccines: Deciphering the Fine-Epitope Specificity
of Cancer-Related Monoclonal Antibodies by Combining Microarray Screening
and Saturation Transfer Difference NMR. J. Am.
Chem. Soc..

[8] Martínez-Sáez N., Peregrina J. M., Corzana F. (2017). Principles of Mucin Structure: Implications
for the Rational Design of Cancer Vaccines Derived from MUC1-Glycopeptides. Chem. Soc. Rev..

[9] Coelho H., Rivas M. D., Grosso A. S., Diniz A., Soares C. O., Francisco R. A., Dias J. S., Compan I., Sun L., Narimatsu Y. (2022). Atomic and Specificity Details of Mucin 1 O-Glycosylation
Process by Multiple Polypeptide GalNAc-Transferase Isoforms Unveiled
by NMR and Molecular Modeling. JACS Au..

[10] Schuman J., Campbell A. P., Koganty R. R., Longenecker B. M. (2003). Probing
the Conformational and Dynamical Effects of O-Glycosylation within
the Immunodominant Region of a MUC1 Peptide Tumor Antigen. J. Pept. Res..

[11] Corzana F., Busto J. H., Jiménez-Osés G., De Luis M. G., Asensio J. L., Jiménez-Barbero J., Peregrina J. M., Avenoza A. (2007). Serine versus Threonine Glycosylation:
The Methyl Group
Causes a Drastic Alteration on the Carbohydrate Orientation and on
the Surrounding Water Shell. J. Am. Chem. Soc..

[12] Martínez-Sáez N., Supekar N. T., Wolfert M. A., Bermejo I. A., Hurtado-Guerrero R., Asensio J. L., Jiménez-Barbero J., Busto J. H., Avenoza A., Boons G. J., Peregrina J. M., Corzana F. (2016). Mucin Architecture behind the Immune Response: Design,
Evaluation and Conformational Analysis of an Antitumor Vaccine Derived
from an Unnatural MUC1 Fragment. Chem. Sci..

[13] Tumoglu B., Keelaghan A., Avci F. Y. (2023). Tn Antigen Interactions of Macrophage
Galactose-Type Lectin (MGL) in Immune Function and Disease. Glycobiology.

[14] Tsuiji M., Fujimori M., Ohashi Y., Higashi N., Onami T. M., Hedrick S. M., Irimura T. (2002). Molecular
Cloning and Characterization
of a Novel Mouse Macrophage C-Type Lectin, MMGL2, Which Has a Distinct
Carbohydrate Specificity from MMGL1 *. J. Biol.
Chem..

[15] Grosso A. S., Diniz A., Soares C. O., Goerdeler F., Gimeno A., Coelho P., Coelho H., Lima C. D. L., Pinheiro B., Lete M. G. (2026). Presentation Is Essential
for Glycan-Lectin Recognition at the Molecular and Cellular Levels:
The Interaction of Tumor-Associated O-Glycans with the Macrophage
Galactose-Type Lectin. JACS Au..

[16] da
Costa V., van Vliet S. J., Carasi P., Frigerio S., García P. A., Croci D. O., Festari M. F., Costa M., Landeira M., Rodríguez-Zraquia S. A., Cagnoni A. J., Cutine A. M., Rabinovich G. A., Osinaga E., Mariño K. V., Freire T. (2021). The Tn Antigen Promotes Lung Tumor Growth by Fostering
Immunosuppression and Angiogenesis via Interaction with Macrophage
Galactose-Type Lectin 2 (MGL2). Cancer Lett..

[17] Neill R. E. O., Cao X. (2019). Co-Stimulatory and Co-Inhibitory Pathways in Cancer
Immunotherapy. Immunotherapy Of Cancer.

[18] Marcelo F., Supekar N., Corzana F., Van Der Horst J. C., Vuist I. M., Live D., Boons G. J. P. H., Smith D. F., Van Vliet S. J. (2019). Identification of a Secondary Binding
Site in Human Macrophage Galactose-Type Lectin by Microarray Studies:
Implications for the Molecular Recognition of Its Ligands. J. Biol. Chem..

[19] Gabba A., Bogucka A., Luz J. G., Diniz A., Coelho H., Corzana F., Cañada F. J., Marcelo F., Murphy P. V., Birrane G. (2021). Crystal Structure of
the Carbohydrate Recognition Domain
of the Human Macrophage Galactose C-Type Lectin Bound to GalNAc and
the Tumor-Associated Tn Antigen. Biochemistry.

[20] Nativi C., Papi F., Roelens S. (2019). Tn Antigen Analogues: The Synthetic
Way to “Upgrade” an Attracting Tumour Associated Carbohydrate
Antigen (TACA). Chem. Commun..

[21] Amedei A., Asadzadeh F., Papi F., Vannucchi M. G., Ferrucci V., Bermejo I. A., Fragai M., De Almeida C. V., Cerofolini L., Giuntini S., Bombaci M., Pesce E., Niccolai E., Natali F., Guarini E., Gabel F., Traini C., Catarinicchia S., Ricci F., Orzalesi L., Berti F., Corzana F., Zollo M., Grifantini R., Nativi C. (2020). A Structurally Simple Vaccine Candidate Reduces Progression
and Dissemination of Triple-Negative Breast Cancer. iScience.

[22] Jiménez-Barbero J., Dragoni E., Venturi C., Nannucci F., Ardá A., Fontanella M., André S., Cañada F. J., Gabius H.-J., Nativi C. (2009). α- O
-Linked Glycopeptide Mimetics:
Synthesis, Conformation Analysis, and Interactions with Viscumin,
a Galactoside-Binding Model Lectin. Chem. -
Eur. J..

[23] Ardá A., Bosco R., Sastre J., Cañada F. J., André S., Gabius H. J., Richichi B., Jiménez-Barbero J., Nativi C. (2015). Structural Insights into the Binding of Sugar Receptors
(Lectins) to a Synthetic Tricyclic Tn Mimetic and Its Glycopeptide
Version. Eur. J. Org. Chem..

[24] Manuelli M., Fallarini S., Lombardi G., Sangregorio C., Nativi C., Richichi B. (2014). Iron Oxide
Superparamagnetic Nanoparticles
Conjugated with a Conformationally Blocked α-Tn Antigen Mimetic
for Macrophage Activation. Nanoscale.

[25] Fallarini S., Papi F., Licciardi F., Natali F., Lombardi G., Maestrelli F., Nativi C. (2023). Niosomes as Biocompatible Scaffolds
for the Multivalent Presentation of Tumor-Associated Antigens (TACAs)
to the Immune System. Bioconjugate Chem..

[26] Fallarini S., Sodini A., Susini F., Lesca G., Scaglione S., Palamà M. E. F., Maestrelli F., Salvatici C., Cefalì F., Guler M. O. (2025). Mucin 1 Antigen Mimetic
Functionalized Mannosylated Peptide Nanofibers for Antigen Uptake
and Immune Modulation. Biomater. Sci..

[27] Pesce E., Sodini A., Palmieri E., Valensin S., Tinti C., Rossi M., De Rosa A., Fragai M., Papi F., Cordiglieri C., Berti F., Grifantini R., Micoli F., Nativi C. (2025). GMMA Decorated with Mucin 1 Tn/STn
Mimetics Elicit Specific Antibodies Response and Inhibit Tumor Growth. Npj Vaccines.

[28] Yonezawa S., Tachikawa T., Shin S., Sato E. (1992). Sialosyl-Tn
Antigen
Its Distribution in Normal Human Tissues and Expression in Adenocarcinomas. Am. J. Clin. Pathol..

[29] Marcos N. T., Pinho S., Grandela C., Cruz A., Harduin-Lepers A., Almeida R., Silva F., Morais V., Costa J., Kihlberg J. (2004). Role of
the Human ST6GalNAc-I and ST6GalNAc-II
in the Synthesis of the Cancer-Associated Sialyl-Tn Antigen. Cancer Res..

[30] Bartolozzi A., Capozzi G., Falciani C., Menichetti S., Nativi C., Bacialli A. P. (1999). Regio- and Stereoselective Synthesis
of 4’-Thiaspiroacetals from Carbohydrates. J. Org. Chem..

[31] Diniz A., Coelho H., Dias J. S., Vliet S. J., Jiménez-Barbero J., Corzana F., Cabrita E. J., Marcelo F. (2019). The Plasticity of the
Carbohydrate Recognition Domain Dictates the Exquisite Mechanism of
Binding of Human Macrophage Galactose-Type Lectin. Chem. – A Eur. J..

[32] Munkley J. (2016). The Role of
Sialyl-Tn in Cancer. Int. J. Mol. Sci..

[33] Brockhausen I., Yang J., Dickinson N., Ogata S., Itzkowitz S. H. (1998). Enzymatic
Basis for Sialyl-Tn Expression in Human Colon Cancer Cells. Glycoconj. J..

[34] Perez S. J. L. P., Fu C. W., Li W. S. (2021). Sialyltransferase Inhibitors for
the Treatment of Cancer Metastasis: Current Challenges and Future
Perspectives. Molecules.

[35] Xia J., Xue J., Locke R. D., Chandrasekaran E. V., Srikrishnan T., Matta K. L. (2006). Synthesis of Fluorinated
Mucin Core 2 Branched Oligosaccharides
with the Potential of Novel Substrates and Enzyme Inhibitors for Glycosyltransferases
and Sulfotransferases. J. Org. Chem..

[36] Kim J., Ryu C., Ha J., Lee J., Kim D., Ji M., Park C. S., Lee J., Kim D. K., Kim H. H. (2020). Structural
and Quantitative Characterization of Mucin-Type O -Glycans and the
Identification of O -Glycosylation Sites in Bovine Submaxillary Mucin. Biomolecules.

[37] Richichi B., Thomas B., Fiore M., Bosco R., Qureshi H., Nativi C., Renaudet O., BenMohamed L. (2014). A Cancer Therapeutic
Vaccine Based on Clustered Tn-Antigen Mimetics Induces Strong Antibody-Mediated
Protective Immunity. Angew. Chem. Int. Ed..

[38] Gracia R., Marradi M., Salerno G., Pérez-Nicado R., Pérez-San Vicente A., Dupin D., Rodriguez J., Loinaz I., Chiodo F., Nativi C. (2018). Biocompatible Single-Chain
Polymer Nanoparticles Loaded with an Antigen Mimetic as Potential
Anticancer Vaccine. ACS Macro Lett..

