# Supplementary material for: Studies on a Sulfoxide-Bridged Tn Antigen Mimetic: Interaction with Macrophage Galactose Lectin and Inhibition of Sialyltransferase ST6GALNAC1
Source: ACS Omega. 2026 Apr 11;11(15):23350–8. doi: 10.1021/acsomega.6c00385 (PMC13103762; doi:10.1021/acsomega.6c00385)
Supplement: Supplementary file 1 [file ao6c00385_si_001.pdf]

Supporting Information

Studies on a Sulfoxide-Bridged Tn Antigen Mimetic: Interaction with Macrophage Galactose Lectin and Inhibition of Sialyltransferase ST6GALNAC1

Andrea Sodini,[1,§] Emanuele Casali,[2,§] Maria Alejandra Travecedo,[3,φ] Filipa Marcelo,[3] Fabrizio Chiodo,[4,5] Filippo Rambaldi,[1] Sandra J. van Vliet,[5,6] Fabio Dall'Olio,[7,*] and Cristina Nativi[1,*]

[§]Co-first authors

[1] Department of Chemistry, DICUS, University of Florence, via della Lastruccia 3-13, 50019 Sesto F.no (FI), Italy

[2] Department of Chemistry, University of Pavia, via Taramelli 10, 27100 Pavia, Italy

[3] UCIBIO – Applied Molecular Biosciences Unit, Department of Chemistry, NOVA School of Science and Technology, NOVA University Lisbon, 2829-516 Caparica, Portugal and Associate Laboratory i4HB - Institute for Health and Bioeconomy, NOVA School of Science and Technology, NOVA University Lisbon, 2829-516 Caparica, Portugal;

[4] Bio-Organic Chemistry Unit, Institute of Biomolecular Chemistry CNR, via Campi Flegrei 34, 80078 Napoli, Italy

[5] Department of Molecular Cell Biology and Immunology Amsterdam UMC, Vrije Universiteit Amsterdam Amsterdam 1081HV, The Nederlands

[6] Amsterdam Institute for Immunology and Infectious Diseases, Cancer Immunology, Amsterdam, the Netherland

[7] Department of Medical and Surgical Sciences, DIMEC, University of Bologna, Via San Giacomo 14, 40126, Bologna, Italy

Corresponding Authors

Cristina Nativi -; orcid.org/0000-0002-6312-3230; Email: cristina.nativi@unifi.it.

Fabio Dall'Olio - orcid.org/0000-0001-9384-2845; Email: fabio.dallolio@unibo

[φ] present address: CIC bioGUNE, Basque Research and Technology Alliance, Bizkaia Technology Park, Ed. 800. E-48160, Derio, Spain.

Table of Content

Materials and Methods

Synthesis of compound **2**	Pag.	S2
Synthesis of compound **7**		S3
Synthesis of compound **8**		S4
Synthesis of compound **5**		S5
Synthesis of compound **4**		S6
Synthesis of compound **3**		S7
Synthesis of compound **9**		S8
Synthesis of compound **11**		S9
Synthesis of **TnSO[BSA]$_{24}$**		S10
Synthesis of **TnSO[BSA]$_{19}$**		S11
Figure S1		S10
Figure S2		S11
Computational details		S12
X-ray analysis		S12
ELISA tests		S13
Figure S3		S14
NMR Binding Studies		S15
Figure S4 – S7		S15-S18
Estimation of the Dissociation Constant (K_D) by NMR		S18
Figure S8		S19
Table S1		S19
Figure S9		S20
Figure S10		S20
Inhibition of ST6GALNAC1		S21
1D and 2D NMR Spectra (Figs. S11-S17)		S22-S34
Cartesian coordinates		S35
References		S38

Materials and Methods

Synthesis of compound 2

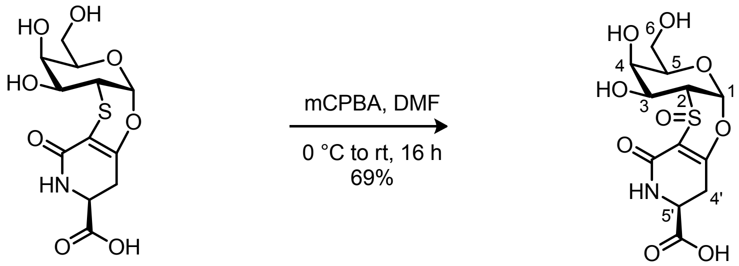

1 **2**

To a suspension of compound **1**[1] (119 mg, 0.35 mmol) in DMF (4 mL) cooled to 0 °C, mCPBA (90 mg, 0.53 mmol) was added. The mixture was stirred at room temperature and monitored via ESI-MS. After 16 h the organic solvent was removed under reduced pressure to afford the crude (250 mg) which was purified by column chromatography on silica gel (CH_2Cl_2:CH_3OH:HCOOH=5:5:0.01) to afford compound **2** (83 mg, 69%) as a white glassy solid.

MW: ($C_{12}H_{15}NO_9S$), 349.31 g/mol.

^{1}H-NMR (500 MHz, D_2O): δ = 5.91 (d, $J_{H1\text{-}H2}$ = 3.0 Hz, 1H, H-1), 4.17-4.12 (m, 2H, H-5 +H-5'), 3.95 (bd, $J_{H4\text{-}H3}$ = 3.0 Hz, 1H, H-4), 3.91-3.77 (A part of an ABX system, $J_{A\text{-}B}$ = 12.0 Hz, $J_{A\text{-}X}$ = 7.9 Hz, H-6a), 3.74-3.66 (m, 2H, H3 + H-6b), 3.62 (dd, $J_{H2\text{-}H3}$ = 12.0 Hz, $J_{H2\text{-}H1}$ = 3.0 Hz, H-2), 3.13-3.08 (A part of an ABX system, $J_{A\text{-}B}$ = 17.5 Hz, $J_{A\text{-}X}$ = 6.7 Hz, H-4'a), 2.94-2.88 (B part of an ABX system, $J_{B\text{-}A}$ = 8.7 Hz, $J_{B\text{-}X}$ = 7.9 Hz, H-4'b) ppm.

^{13}C-NMR (125 MHz, D_2O): δ = 176.3 (Cq, CO), 169.9 (Cq, CO), 166.7 (Cq, C-3'), 103.8 (Cq, C-2'), 92.5 (CH, C-1), 74.7 (CH, C-5'), 67.8 (CH, C-4), 63.9 (CH, C-3), 61.0 (CH_2, C-6), 54.8 (CH, C-2), 52.2 (CH, C-5), 31.6 (CH_2, C-4') ppm.

Synthesis of compound 7

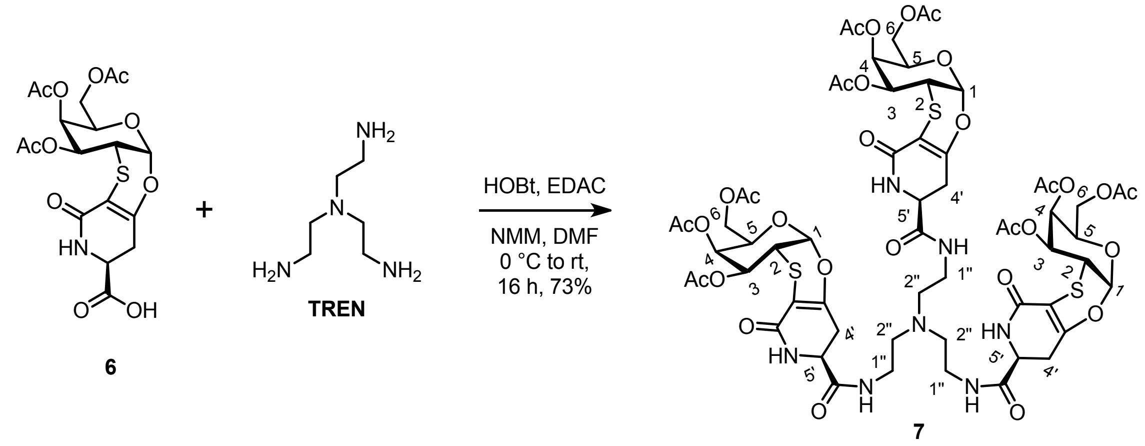

To a solution of compound **6**[2] (291 mg, 0.63 mmol) in DMF (2 mL) cooled to 0 °C HOBt (110 mg, 0.81 mmol) and EDAC (159 mg, 0.83 mmol) were added, after 10 min a solution of **TREN** (23 mg, 0.16 mmol) and NMM (95 µL, 0.86 mmol) in DMF (3 mL) was added. The mixture was stirred at rt for 16 h until TLC (CH_2Cl_2:CH_3OH=8:2) showed the disappearance of starting material. The mixture was diluted with EtOAc (50 mL) and washed with H_2O (2 x 50 mL), the organic layer was dried over Na_2SO_4 and concentrated to dryness to give a crude (215 mg), which was purified on column chromatography (CH_2Cl_2:CH_3OH=93:7) to give compound **7** (168 mg, 73% yield) as a white foam.

MW: ($C_{60}H_{75}N_7O_{30}S_3$), 1470,46 g/mol.

ESI-MS *m/z* (%): 1492.00 (100) $[M + Na]^+$.

$[\alpha]_D^{23}$: +122 (c 0.055 in CH_3OH).

^{1}H-NMR (500 MHz, CD_3OD): δ = 5.81 (d, $J_{H1\text{-}H2}$ = 2.8 Hz, 1H, H-1), 5.42-5.39 (m, 1H, H-4), 5.09 (dd, $J_{H3\text{-}H2}$ = 11.7 Hz, $J_{H3\text{-}H4}$ = 3.1 Hz, 1H, H-3), 4.53 (bt, $J_{H5\text{-}H6}$ = 6.8 Hz, 1H, H-5), 4.26 (t, $J_{H5'\text{-}H4'}$ = 6.6 Hz, 1H, H-5'), 4.20-4.16 (A part of an ABX system, $J_{A\text{-}B}$ = 11.3 Hz, $J_{A\text{-}X}$ = 6.5 Hz, 1H, H-6a), 4.16-4.11 (B part of an ABX system, $J_{B\text{-}A}$ = 11.3 Hz, $J_{B\text{-}X}$ = 6.8 Hz, 1H, H-6b), 3.73 (dd, $J_{H2\text{-}H3}$ = 11.7 Hz, $J_{H2\text{-}H1}$ = 2.8 Hz, 1H, H-2), 3.37-3.31 (m, 1H, one H of H-1''), 3.26-3.19 (m, 1H, 1H of H-1''), 3.16-3.09 (A part of an ABX system, $J_{A\text{-}B}$ = 16.8 Hz, $J_{A\text{-}X}$ = 7.1 Hz, 1H, H-4'a), 2.91-2.85 (B part of an ABX system, $J_{B\text{-}A}$ = 16.8 Hz, $J_{B\text{-}X}$ = 6.0 Hz, 1H, H-4'b), 2.65-2.54 (m, 1H, H-2''), 2.16 (s, 3H, *Ac*), 2.04 (s, 3H, *Ac*), 2.01 (s, 3H, *Ac*) ppm.

^{13}C-NMR (125 MHz, CD_3OD): δ = 172.9 (Cq, CO), 172.1 (Cq, CO), 171.9 (Cq, CO), 171.5 (Cq, CO), 166.9 (Cq, CO), 157.6 (Cq), 97.6 (CH, C-1), 97.0 (Cq), 70.1 (CH, C-5), 68.7 (CH, C-4), 67.2 (CH, C-3),

62.8 (CH_2, C-6), 55.0 (CH_2, C-2''), 53.2 (CH, C-5'), 39.4 (CH_2, C-1''), 37.4 (CH, C-2), 31.7 (CH_2, C-4''), 20.7 ($2CH_3$, *Ac*), 20.6 (CH_3, *Ac*) ppm.

Synthesis of compound 8

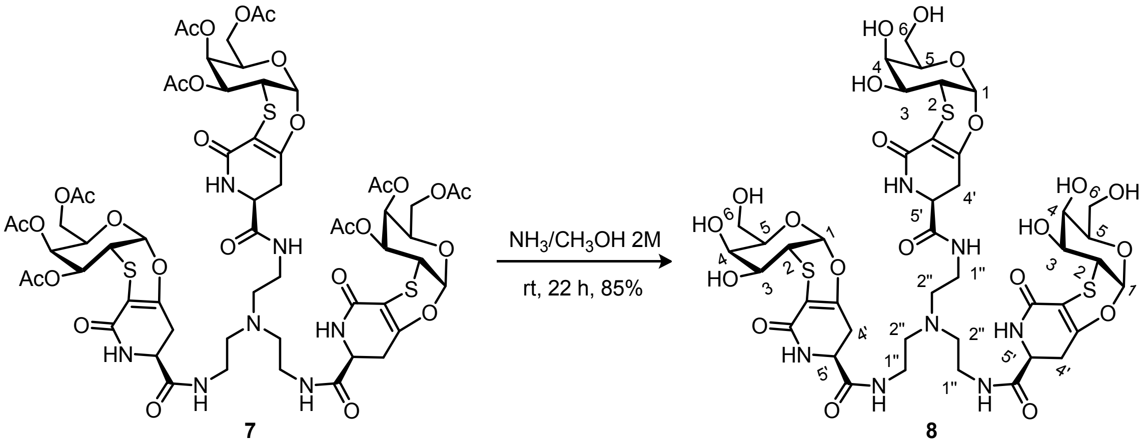

To a solution of **7** (135 mg, 0.092 mmol) in CH_3OH (2.5 mL) NH_3 in CH_3OH 4M (2.5 mL, 10 mmol) was added. The mixture was stirred at rt for 22 h until ^{1}H-NMR control showed the conclusion of the reaction. The precipitate was filtered and washed with CH_3OH (3 x 2 mL) to give pure compound **8** (84 mg, 0.077 mmol, 85% yield) as a white solid.

MW: ($C_{42}H_{57}N_7O_{21}S_3$), 1092.13 g/mol.

ESI-MS *m/z* (%): 1114.00 (100) $[M + Na]^+$.

MP: 218-223 °C dec.

$[\alpha]_D^{24}$: +166 (c 0.05 in H_2O).

^{1}H-NMR (500 MHz, D_2O): δ = 5.83 (d, J_{H1-H2} = 2.7 Hz, 1H, H-1), 4.34-4.30 (X part of an ABX system, J_{X-A} = 7.0 Hz, J_{X-B} = 4.8 Hz, 1H, H-5'), 4.18-4.13 (X part of an ABX system, J_{X-A} = 7.1 Hz, J_{X-B} = 4.7 Hz, 1H, H-5), 4.06-4.04 (m, 1H, H-4), 3.86-3.80 (A part of an ABX system, J_{A-B} = 11.8 Hz, J_{A-X} = 7.8 Hz, 1H, H-6a), 3.79-3.72 (m, 2H, H3 + H-6b), 3.52 (dd, J_{H2-H3} = 11.2 Hz, J_{H2-H1} = 2.7 Hz, 1H, H-2), 3.41-3.33 (m, 1H, one H of H-1''), 3.28-3.21 (m, 1H, one H of H-1''), 3.20-3.13 (A part of an ABX system, J_{A-B} = 17.2 Hz, J_{A-X} = 7.6 Hz, 1H, H-4'a), 2.89-2.83 (B part of an ABX system, J_{B-A} = 17.2 Hz, J_{B-X} = 4.6 Hz, 1H, H-4'b), 2.72-2.63 (m, 2H, H-2'') ppm.

^{13}C-NMR (125 MHz, D_2O): δ = 171.8 (Cq, CO), 167.2 (Cq, CO), 158.1 (Cq), 96.7 (CH, C-1), 94.1 (Cq), 73.4 (CH, C-5), 68.4 (CH, C-4), 66.4 (CH, C-3), 61.0 (CH_2, C-6), 52.6 (CH_2, C-2''), 51.3 (CH, C-5'), 38.1 (CH, C-2), 37.7 (CH_2, C-1''), 30.3 (CH_2, C-4') ppm.

Synthesis of compound 5

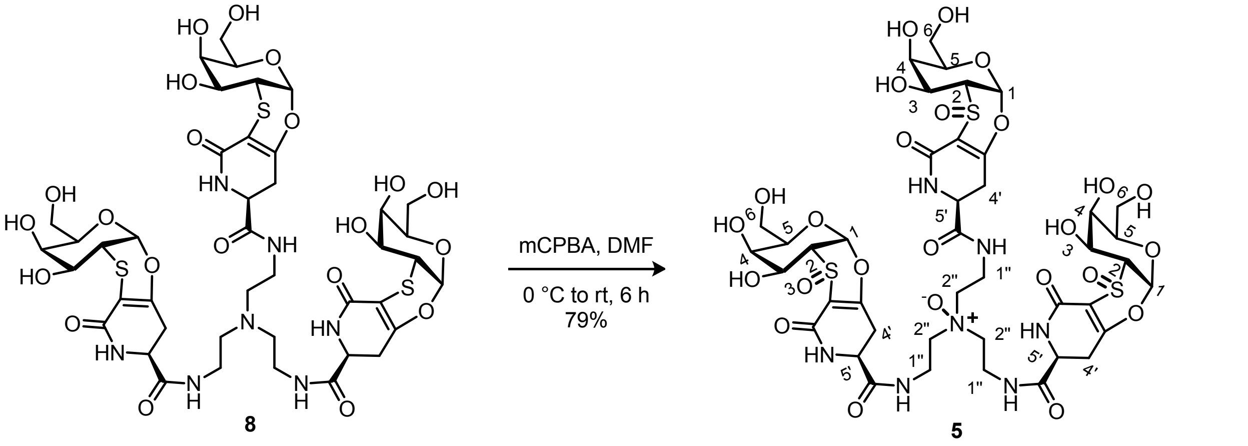

To a solution of compound **8** (43 mg, 0.04 mmol) in DMF (2 mL) cooled to 0 °C, mCPBA (34 mg, 0.20 mmol) was added. The mixture was stirred at room temperature and monitored via ESI-MS. After 6 h the organic solvent was removed under reduced pressure to afford the crude (79 mg) which was purified by several washings with CH_3OH (6 x 2 mL) affording compound **5** (35 mg, 79%) as a white glassy solid.

MW: ($C_{42}H_{57}N_7O_{25}S_3$), 1156.13 g/mol.

^{1}H-NMR (500 MHz, D_2O): δ = 5.91 (d, $J_{H1\text{-}H2}$ = 1.1 Hz, 1H, H-1), 4.36 (dd, $J_{H5'\text{-}H4'a}$ = 7.5 Hz, $J_{H5'\text{-}H4'b}$ = 4.7 Hz, H-5'), 4.11 (dd, $J_{H5\text{-}H6a}$ = 7.9 Hz, $J_{H5\text{-}H6b}$ = 4.1 Hz, H-5), 3.95-3.93 (m, 1H, H-4), 3.81-3.77 (A part of an ABX system, $J_{A\text{-}B}$ = 12.0 Hz, $J_{A\text{-}X}$ = 7.9 Hz, H-6a), 3.74-3.69 (B part of an ABX system, $J_{B\text{-}A}$ = 8.7 Hz, $J_{B\text{-}X}$ = 4.11 Hz, H-6b), 3.65-3.59 (m, 4H, H-2 + H-3 + H-2''), 3.45-3.40 (m, 2H, H-1''), 3.27 (dd, $J_{H4'a\text{-}H4'b}$ = 17.6 Hz, $J_{H4'a\text{-}H5'}$ = 7.6 Hz, 1H, H-4'a), 3.00 (dd, $J_{H4'b\text{-}H4'a}$ = 17.6 Hz, $J_{H4'b\text{-}H5'}$ = 4.7 Hz, 1H, H-4'a) ppm.

^{13}C-NMR (125 MHz, D_2O): δ = 171.7 (Cq, CO), 168.2 (Cq, CO), 160.4 (Cq), 98.7 (CH, C-1), 94.1 (Cq), 73.4 (CH, C-5), 68.4 (CH, C-4), 66.4 (CH, C-3), 61.4 (CH_2, C-6), 53.6 (CH_2, C-2''), 51.3 (CH, C-5'), 38.2 (CH, C-2), 38.9 (CH_2, C-1''), 31.3 (CH_2, C-4') ppm.

Synthesis of compound 4

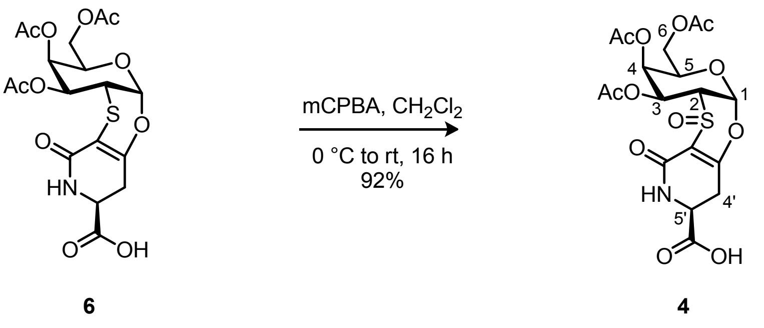

To a solution of compound **6** (100 mg, 0.22 mmol) in CH_2Cl_2 (5 mL) cooled to 0 °C, mCPBA (56 mg, 0.33 mmol) was added. The mixture was stirred at room temperature and monitored by TLC. After 16 h the organic solvent was removed under reduced pressure to afford the crude (153 mg) which was purified by column chromatography on silica gel (CH_2Cl_2:CH_3OH=85:15 to 70:30) to yield compound **4** (95 mg, 92%) as a white glassy solid.

MW: ($C_{18}H_{21}NO_{12}S$), 475.42 g/mol.

^{1}H-NMR (500 MHz, DMSO-d$_6$): δ = 7.29 (s, 1H, NH), 5.92 (d, J_{H1-H2} = 3.1 Hz, 1H, H-1), 5.29 (bd, J_{H4-H3} = 2.9 Hz, 1H, H-4), 5.01 (dd, J_{H3-H2} = 12.4 Hz, J_{H3-H4} = 2.9 Hz, 1H, H-3), 4.68 (dt, J_{H5-H6} = 6.4 Hz, 1H, H-5), 4.10 (d, J_{H6-H5} = 6.4 Hz, 2H, H-6), 3.77 (dd, $J_{H5'-H4'a}$ = 11.1 Hz, $J_{H5'-H4'b}$ = 5.8 Hz, 1H, H-5'), 3.50 (dd, J_{H2-H3} = 12.4 Hz, J_{H2-H1} = 3.1 Hz, 1H, H-2), 2.95 ($J_{H4'b-H4'a}$ = 17.3 Hz, $J_{H4'b-H5'}$ = 5.8 Hz, 1H, H-4'b), 2.71 ($J_{H4'a-H4'b}$ = 17.3 Hz, $J_{H4'a-H5'}$ = 11.1 Hz, 1H, H-4'a), 2.15-8 (s, 3H, Ac), 2.03 (s, 3H, Ac),), 1.96 (s, 3H, Ac),) ppm.

^{13}C-NMR (125 MHz, DMSO-d$_6$): δ = 170.0 (Cq, CO), 169.9 (Cq, CO), 169.1 (Cq, CO), 167.4 (Cq, CO), 163.5 (Cq, CO), 106.4 (Cq, C-3'), 91.6 (CH, C-1), 69.5 (CH, C-5), 66.2 (CH, C-4), 64.4 (CH, C-3), 61.5 (CH_2, C-6), 52.5 (CH, C-2), 51.6 (CH, C-5'), 31.7 (CH_2, C-4'), 20.6 (CH_3, Ac), 20.4 (CH_3, Ac), 20.3 (CH_3, Ac) ppm

Synthesis of compound 3

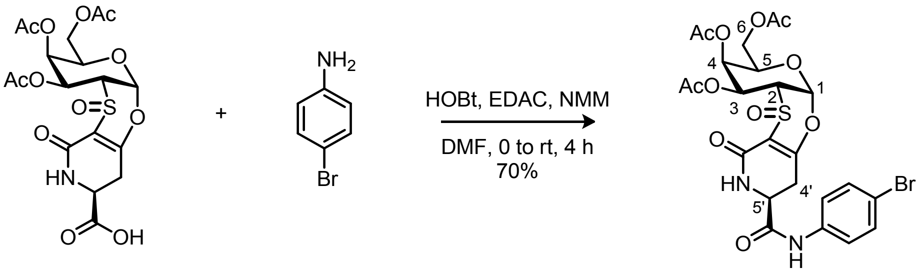

4 **3**

To a solution of compound **4** (66 mg, 0.14 mmol) in DMF (4 mL) cooled to 0 °C HOBt (75 mg, 0.56 mmol) EDAC (106 mg, 0.56 mmol) and NMM (60 μL, 0.56 mmol) were added. After 10 min stirring at 0 °C, 4-bromoaniline (47 mg, 0.28 mmol) was added and the mixture was stirred at rt for 4 h until TLC (CH_2Cl_2:CH_3OH=8:2) showed the disappearance of starting material. The mixture was diluted with EtOAc (50 mL) and washed with citric acid 10 % (w/w) (3 x 50 mL) $NaHCO_3$ s.s (50 mL) and eventually brine (50 mL). The organic layer was dried over Na_2SO_4 and concentrated to dryness to give a crude (96 mg), which was purified on column chromatography (CH_2Cl_2:acetone=6:4) affording compound **3** (65 mg, 70% yield) as a white foam.

MW: ($C_{24}H_{25}BrN_2O_{11}S$), 629.43 g/mol.

^{1}H-NMR (500 MHz, CDCl$_3$): δ = 8.89 (s, H, NH), 7.45-7.42 (AA' part of an AA'BB' system, $J_{A\text{-}B}$ = 9.0 Hz, 1H, Ar), 7.39-7.37 (BB' part of an AA'BB' system, $J_{B\text{-}A}$ = 9.0 Hz, 1H, Ar), 5.98 (d, $J_{H1\text{-}H2}$ = 3.1 Hz, 1H, H-1), 5.43 (bd, $J_{H4\text{-}H3}$ = 2.8 Hz, 1H, H-4), 4.85 (dd, $J_{H3\text{-}H2}$ = 12.5 Hz, $J_{H3\text{-}H4}$ = 3.1 Hz, 1H, H-3), 4.47 (bt, $J_{H5\text{-}H6}$ = 6.6 Hz, 1H, H-5), 4.39-4.35 (m, 1H, H-5'), 4.19 (d, $J_{H6\text{-}H5}$ = 6.6 Hz, 2H, H-5), 3.70 (dd, $J_{H2\text{-}H3}$ = 12.6 Hz, $J_{H2\text{-}H1}$ = 2.9 Hz, 3H, H-2), 3.20-3.15 (A part of an ABX system, $J_{A\text{-}B}$ = 17.1 Hz, $J_{A\text{-}X}$ = 6.7 Hz, H-4'a), 3.15-3.10 (B part of an ABX system, $J_{B\text{-}A}$ = 17.1 Hz, $J_{B\text{-}X}$ = 7.2 Hz, H-4'b), 2.20 (s, 3H, Ac), 2.08 (s, 3H, Ac), 2.00 (s, 3H, Ac) ppm.

^{13}C-NMR (125 MHz, CDCl$_3$): δ = 211.0 (Cq, CO), 170.5 (Cq, CO), 170.0 (Cq, CO), 167.7 (Cq, CO), 166.8 (Cq, CO), 164.7 (Cq), 137.0 (Cq, Ar), 131.9 (2CH, Ar), 121.8 (2CH, Ar), 117.3 (Cq, Ar), 106.8 (Cq, C-3'), 92.7 (CH, C-1), 70.2 (CH, C-5), 66.0 (CH, C-4), 64.4 (CH, C-3), 61.4 (CH_2, C-6), 53.5 (CH, C-2), 52.7 (CH, C-5'), 29.3 (CH_2, C-4'), 20.7 (CH_3, Ac), 20.6 (CH_3, Ac), 20.5 (CH_3, Ac) ppm.

Synthesis of compound 9

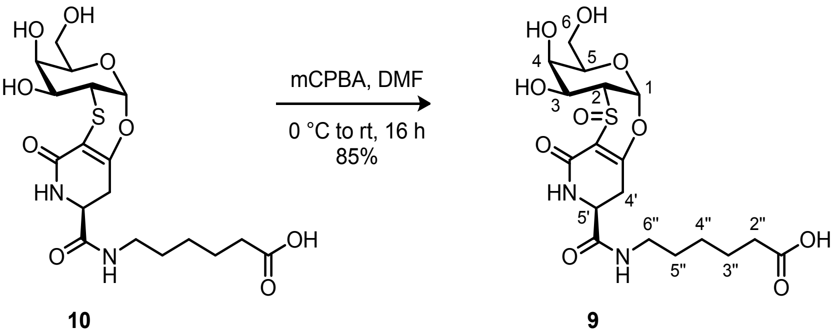

10 **9**

To a solution of compound **10**[3] (100 mg, 0.22 mmol) in DMF (5 mL) cooled to 0 °C, mCPBA (56 mg, 0.33 mmol) was added. The mixture was stirred at room temperature and monitored by TLC (CH_2Cl_2:CH_3OH=7:3). After 16 h the organic solvent was removed under reduced pressure to afford the crude (153 mg) which was purified by column chromatography on silica gel (CH_2Cl_2:CH_3OH=85:15 to 70:30) to yield compound **9** (95 mg, 85%) as a white glassy solid.

MW: ($C_{18}H_{26}N_2O_{10}S$), 462.47 g/mol.

^{1}H-NMR (500 MHz, D_2O): δ = 5.89 (d, $J_{H1\text{-}H2}$ = 2.6 Hz, 1H, H-1), 4.31 (dd, $J_{H5'\text{-}H4'a}$ = 7.5 Hz, $J_{H5'\text{-}H4'b}$ = 5.4 Hz, 1H, H-5'), 4.12 (dd, $J_{H5\text{-}H6a}$ = 7.9 Hz, $J_{H5\text{-}H6b}$ = 4.1 Hz, 1H, H-5), 3.94 (bd, $J_{H4\text{-}H3}$ = 2.6 Hz, 1H, H-4), 3.79 (dd, $J_{H6a\text{-}H6b}$ = 12.0 Hz, $J_{H6a\text{-}H5}$ = 7.9 Hz, 1H, H-6a), 3.71 (dd, $J_{H6b\text{-}H6a}$ = 12.0 Hz, $J_{H6b\text{-}H5}$ = 4.1 Hz, 1H, H-6b), 3.65-3.63 (m, 2H, H-6''), 3.24 (dd, $J_{H4'a\text{-}H4'b}$ = 17.6 Hz, $J_{H4'a\text{-}H5'}$ = 7.6 Hz, 1H, H-4'a), 3.19-3.15 (m, 2H, H-2 + H-3), 2.94 (dd, $J_{H4'b\text{-}H4'a}$ = 17.6 Hz, $J_{H4'b\text{-}H5'}$ = 5.4 Hz, 1H, H-4'b), 2.29 (t, $J_{H2''\text{-}H3''}$ = 7.3 Hz, 2H, H-2''), 1.57-1.49 (m 2H, H-5''), 1.49-1.42 (m, 2H, H-3''), 1.27-1.20 (m, 2H, H-4'') ppm.

^{13}C-NMR (125 MHz, D_2O): δ = 182.9 (Cq, CO), 172.6 (Cq, CO), 167.8 (Cq, CO), 157.7 (Cq), 98.3 (CH, C-1), 96.8 (Cq), 75.0 (CH, C-5), 70.4 (CH, C-4), 67.3 (CH, C-3), 62.6 (CH_2, C-6), 53.4 (CH, C-5'), 40.7 (CH, C-2), 40.5 (CH_2, C-6''), 39.0 (CH_2, C-2''), 32.1 (CH, C-4'), 30.0 (CH_2, C-3''), 27.9 (CH_2, C-5''), 27.2 (CH_2, C-4'') ppm.

Synthesis of compound 11

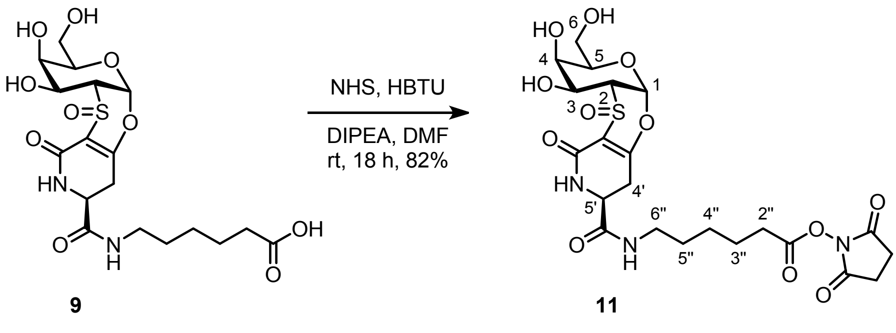

To a dispersion of compound **9** (68 mg, 0.15 mmol) in anhydrous DMF (5 mL), HBTU (200 mg, 0.52 mmol) and DIPEA (70 µL, 0.39 mmol) were added. The mixture was stirred at rt until dissolution and then NHS (50 mg, 0.42 mmol) was added. The mixture was stirred at rt and monitored via ESI-MS for 18 h until disappearance of starting material. The dispersion was evaporated under reduced pressure to give crude (320 mg) which was dispersed in EtOAc (2 mL). The precipitate was filtered and washed with EtOAc (3 x 2 mL) to give activated compound **11** (65 mg, 82%) as a light-brown cerous solid which was used immediately to prevent degradation.

MW: ($C_{22}H_{29}N_3O_{12}S$), 559.54 g/mol.

^{1}H-NMR (500 MHz, CD_3OD): δ = 5.88 (d, $J_{H1\text{-}H2}$ = 2.8 Hz, 1H, H-1), 4.23 (t, $J_{H\text{-}H}$ = 6.7 Hz, 1H), 4.05 (bt, $J_{H\text{-}H}$ = 6.1 Hz, 1H), 3.90 (d, $J_{H4\text{-}H3}$ = 2.5 Hz, 1H, H-4), 3.84-3.80 (A part of an ABX system, $J_{A\text{-}B}$ = 11.6 Hz, $J_{A\text{-}X}$ = 6.8 Hz, 1H, H-6a), 3.78-3.73 (B part of an ABX system, $J_{B\text{-}A}$ = 11.6 Hz, $J_{B\text{-}X}$ = 5.2 Hz, 1H, H-6b), 3.62 (dd, $J_{H3\text{-}H2}$ = 11.8 Hz, $J_{H3\text{-}H4}$ = 2.8 Hz, 1H, H-3), 3.52 (dd, $J_{H2\text{-}H3}$ = 11.8 Hz, $J_{H2\text{-}H1}$ = 2.8 Hz, 1H, H-3), 3.27-3.23 (m, 2H), 3.15 (dd, $J_{H4'a\text{-}H4'b}$ = 17.6 Hz, $J_{H4'a\text{-}H5'}$ = 7.6 Hz, 1H, H-4'a), 3.00 (dd, $J_{H4'b\text{-}H4'a}$ = 17.6 Hz, $J_{H4'b\text{-}H5'}$ = 5.4 Hz, 1H, H-4'b), 2.84 (s, 4H, *OSu*), 1.78-1.70 (m 2H, H-5''), 1.59-1.53 (m, 2H, H-3''), 1.49-1.43 (m, 2H, H-4'') ppm.

Synthesis of TnSO[BSA]$_{24}$

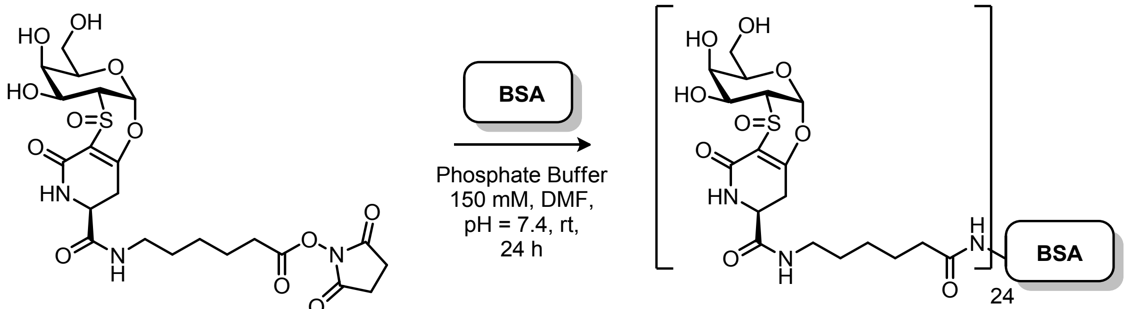

11 **TnSO[BSA]$_{24}$**

BSA (12 mg, 0.18 μmol) was dissolved in NAPi Phosphonate Buffer (2 mL, 150 mM, pH 7.4) and slowly treated with a solution of **11** (38 mg, 67 μmol) dissolved in anhydrous DMF (150 μL). The mixture was stirred at rt for 24 h, the success of the reaction was proved by an SDS-PAGE, then the conjugate was centrifuged to remove solid precipitate. The water layer was purified by HPLC-SEC and concentrated to 100 μM using a 35 kDa MWCO membrane centrifugal device (Millipore). The number of functionalized lysine was confirmed by MALDI/TOF-TOF UltraFlex III analysis: about 24 glycans were linked to BSA (**TnSO[BSA]$_{24}$**) (see Figure S1)

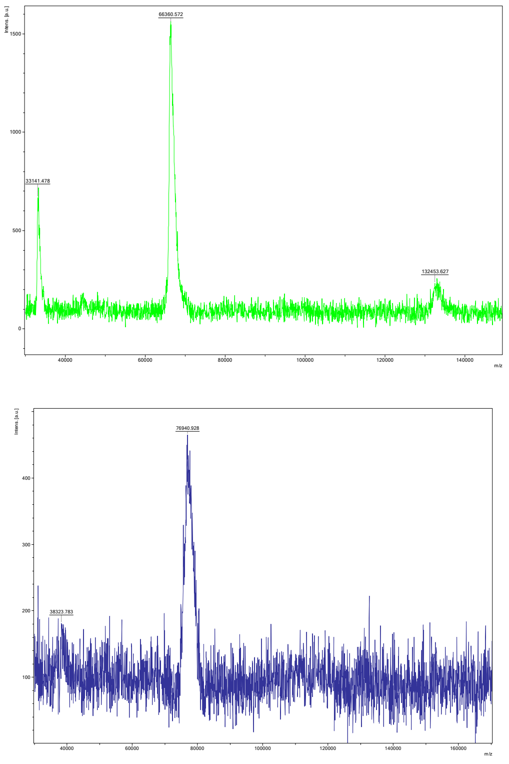

Figure S1. MALDI-TOF/TOF MS spectra of BSA alone (green plot), BSA functionalized with Tn mimetic sulfoxide derivative **11** (blue plot); the mass difference (10580 Da) indicates that 24 glycan units have been linked to BSA.

Synthesis of TnSO[BSA]$_{19}$

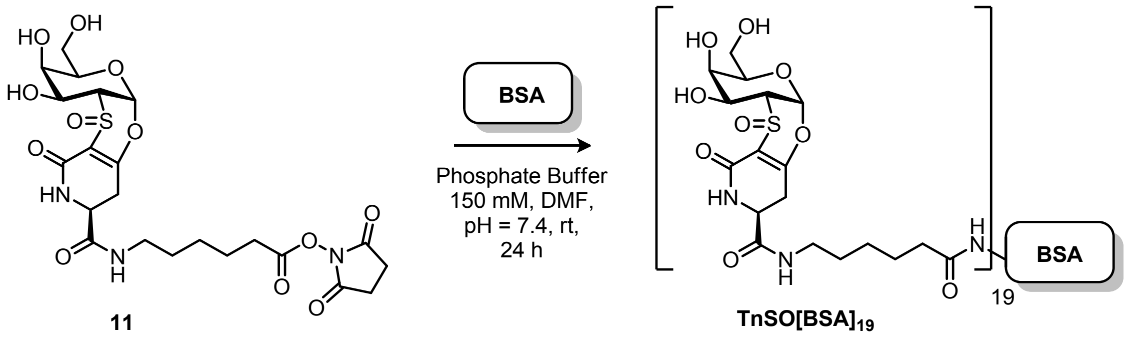

BSA (12 mg, 0.18 µmol) was dissolved in NAPi Phosphonate Buffer (2 mL, 150 mM, pH 7.4) and slowly treated with a solution of **11** (19 mg, 34 µmol) dissolved in anhydrous DMF (150 µL). The mixture was stirred at rt for 24 h, the success of the reaction was proved by an SDS-PAGE, then the conjugate was centrifuged to remove solid precipitate. The water layer was purified by HPLC-SEC and concentrated to 100 µM using a 35 kDa MWCO membrane centrifugal device (Millipore). The number of functionalized lysine was confirmed by MALDI/TOF-TOF UltraFlex III analysis: about 19 glycans were linked to BSA (**TnSO[BSA]$_{19}$**) (see Figure S2)

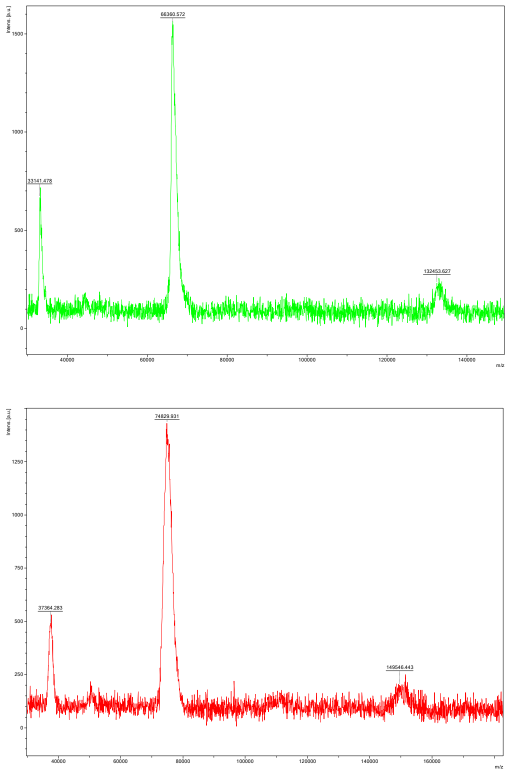

Figure S2. MALDI-TOF/TOF MS spectra of BSA alone (green plot), BSA functionalized with Tn mimetic sulfoxide derivative **11** (red plot); the mass difference (8469 Da) indicates that 19 glycan units have been linked to BSA.

Computational details

Conformational sampling was carried out at the outset of the structural investigation using a combination of manual searches and the open-source *CREST* tool, which is part of the *xTB* software package.[4] All structures involved in the mechanistic study were subsequently optimized with the Gaussian 16 program package,[5] employing the B3LYP hybrid functional.[6] The 6-31G(d) basis set was applied to all atoms,[7] and solvent effects were modeled using the polarizable continuum model (PCM).[8] Frequency calculations were performed for each optimized structure to confirm its nature as either a minimum or a transition state (TS). In addition, intrinsic reaction coordinate (IRC) calculations were carried out to verify the continuity of the reaction pathway, connecting TSs to the corresponding reactants, intermediates, or products.[9]

The final reported energies were thermally corrected to Gibbs free energies using a higher level of theory via single-point (SP) calculations. These calculations were performed on the optimized geometries using the M06-2X functional,[10] including D3 version of Grimme's dispersion with the original D3 damping function,[11] in combination with the def2-TZVP valence triple-ζ polarized basis set for all atoms.[12] Solvent effects were again accounted for using the PCM model.[8]

X-ray structure

Experimental details for crystal structure determination of compound 3 (ASO25_051)

Chemical formula moiety (C_{24} H_{25} Br N_2 O_{11} S, HO),

Chemical formula sum C_{24} H_{26} Br N2 O12 S.

M=646.44, Monoclinic, space group P 21, checked for P 21/m group with no result.

a=16.0873(8), b=5.3758(2), c=16.6332(8)Å, β=110.065(3), V=1351.2(1)Å^3, Z=2, D_c=1.589, μ=3.395mm^{-1}, F (000) = 662.

38076 reflections were collected with a 2.828<θ< 68.363 range with a completeness to theta 100%; 4967 were unique, the parameters were 368 and the final R index was 0.0617 for reflections having I>2σI.

A colourless prismatic crystal (0.040 x 0.040 x 0.220) was used for data collection.

Hydrogen atoms were all assigned in calculated positions as riding atoms.

Non-hydrogen atoms were refined anisotropically whereas hydrogen atoms were refined as isotropic.

A water molecule is cocrystallized in the asymmetric unit.

The two hydrogen atoms could not be assigned from the Fourier difference map. One of the two hydrogens was found by searching for possible hydrogen bonds in the cell. The position of the other remained undetermined, in the portion of space delimited by 109° from the first H-atom.

In molecular packing, two strong (intra and inter molecular) hydrogen bonds are present and listed below.

O12--H1A<br>0.732 Å	O12----O2 (0)<br>2.867 Å	H1A---O2 (0)<br>2.151 Å	O12--H1A---O2 (0)<br>165.80°
N1—H1<br>0.880 Å	N1----O12 (1)<br>2.925 Å	H1---O12 (1)<br>2.079 Å	N1--H1---O12 (1) 160.99

The relative symmetry operations are: (0) x, y, z

(1) -x+1, +y-1/2, -z+2

Complete crystallographic data, in CIF format, has been deposited with the Cambridge Crystallographic Data Centre. Data can be obtained free of charge from the Cambridge Crystallographic Data Centre via www.ccdc.cam.ac.uk/data_request/cif.

CCDC 2517100 contains the supplementary crystallographic data for this structure.

A single crystal was mounted in a loop and coated with a trace of silicone oil.

Data collection were performed at 173° K with a Bruker Apex-II CCD diffractometer, using a Cu–Kα (λ = 1.54184 Å) radiation.

Data were collected, reflections were indexed and processed, and the files scaled and corrected for absorption, using Bruker APEX2, SAINT and SADABS-2016/2 routine.

The integrated intensities, measured using the φ and ω scan mode, were corrected for Lorentz and polarization effects.

Structures were solved by direct methods of SIR2019,[13] the refinement was performed using the full-matrix least squares on F^2 provided, within WinGX v.2013.3 routine,[14] by SHELXL2018.[15]

ELISA tests

To confirm the lack of binding between the sulfoxide **2** and MGL, a solid-phase assay (ELISA-based) was performed (see Figure S3). The multivalent derivatives $TnSO[BSA]_{19}$ and $TnSO[BSA]_{24}$ were prepared as previously reported[2] and screened vs. MGL by coating them on ELISA plate. 50 µL from a 40µg/mL $TnSO[BSA]_{19}$ or $TnSO[BSA]_{24}$ solution in PBS (10 mM, pH 7.4), were used to coat NUNC MaxiSorp wells (overnight, 4°C). After discarding and washing the wells (2 x 150 µL) with calcium and magnesium-containing buffer TSM (20 mM tris(hydroxymethyl)aminomethane (Tris)-HCl, pH 8.0; 150 mM NaCl; 1 mM $CaCl_2$; 2 mM $MgCl_2$), wells were blocked with 80 µL 1% BSA solution (Sigma-Aldrich, lyophilized powder, ≥96%, agarose gel electrophoresis) in TSM at room temperature for 30 min. The blocking solution was discarded and 50 µL of MGL-Fc chimera at 1 µg/mL were added. After 1 h at room temperature, wells

were washed with TSM (2 x 150 μL) and 100 μL of anti-human horseradish peroxidase (0.3 μg/mL, Goat anti-human IgG-HRP from JacksonImmuno) were added. After 30 min, wells were washed with TSM (2 x 150 μL). Finally, 100 μL of a substrate solution (3,3′,5,5′- tetramethylbenzidine, TMB, in citric/acetate buffer, pH 4, and H_2O_2) were added. After 10 min at room temperature the reaction was stopped with 50 μL of H_2SO_4 (0.8 M) and the optical density (OD) was measured at 450 nm in an ELISA reader. The experiment was performed in duplicate, and data were normalized over the signal at 450 nm from the positive control used for MGL (Polyacrylamide polymer, GalNAcα-$OCH_2CH_2CH_2NH_2$ 0030-PA, PAA-Tn, at 10μg/mL).

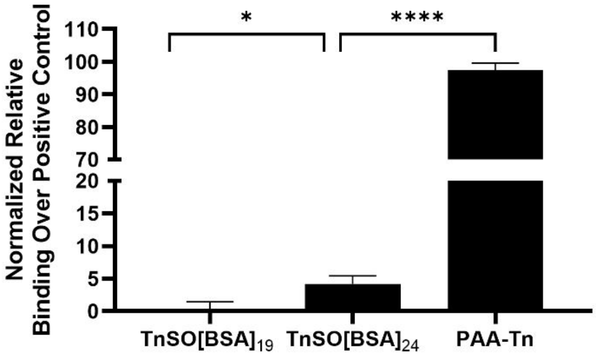

Figure S3. BSA glycosylated with **19** and **24** residues of sulfoxide **2**, namely TnSO[BSA]$_{19}$ and TnSO[BSA]$_{24}$ respectively, were used to coat ELISA wells. The binding with MGL was studied exploiting the Fc fragment on the MGL-chimera used in a calcium-containing buffer. The experiment was performed twice in duplicate, and data were normalized over signal from MGL positive control (polyacrylamide polymers coated with Tn antigen). Error bars indicate standard deviations. Ordinary one-way ANOVA multiple comparisons analysis was performed (Tukey's multiple comparisons test) with Alpha=0.05 using GraphPad Prism 10.6. **** $p \leq 0.0001$ and * $p \leq 0.05$

NMR binding studies

All the NMR experiments were acquired on a Bruker Avance III 600 MHz spectrometer equipped with a 5-mm inverse detection triple-resonance cryogenic probe head with z-gradients in 3 mm NMR tubes at 293 K. Recombinant MGL-CRD was uniformly ^{15}N-labeled and prepared in buffer with 20 mM Ca^{2+} (10 mM Tris buffer (pH 7.4) containing 75 mM NaCl and 20 mM $CaCl_2$) as described.[16] ^{1}H–^{15}N HSQC spectra of MGL-CRD (200 μM) were obtained following titration with increasing concentrations of the monovalent compound **2** up to a 1:150 protein-to-ligand molar ratio (Figure S4), or with the trivalent compound **5** at a 1:17 protein-to-ligand molar ratio (Figure S5). The ^{1}H-^{15}N-HSQC experiments were acquired with 2048x128 points and 32 scans in a spectral window of 9615.4 Hz (centre at 2801 Hz) x 1946 Hz (centre at 7175 Hz), in ^{1}H and ^{15}N sweep's width, respectively. The data was processed with Bruker TopSpin 3.5, CcpNMR Analysis Version 2.4.

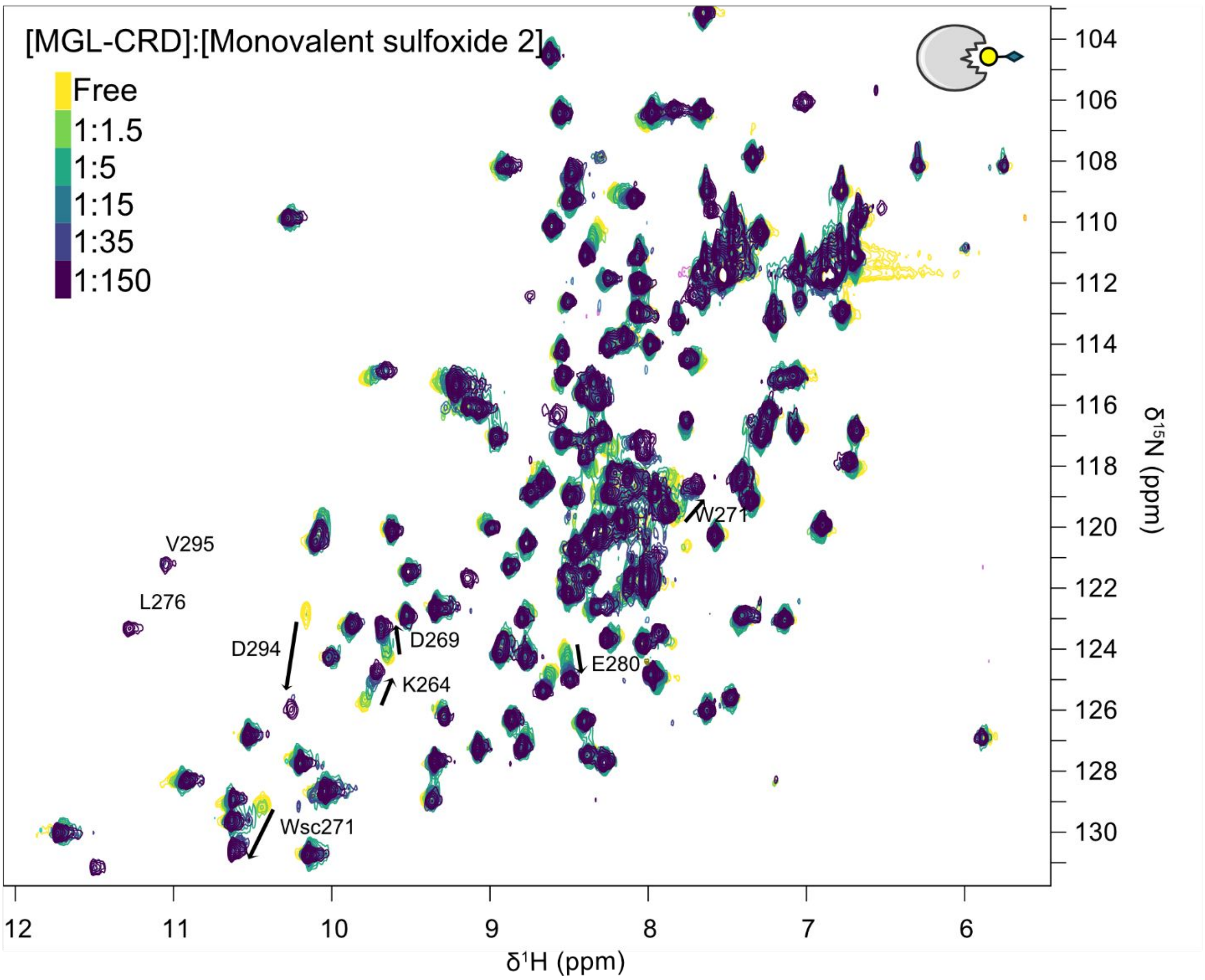

Figure S4. Region of 2D ^{1}H–^{15}N HSQC spectra of MGL-CRD/compound **2** complexes superimposed throughout the titration with the corresponding references: MGL-CRD apo (yellow), with respect to MGL-CRD in the presence of compound **2** (in 1:1.5, 5, 15, 35 and 150 molar ratio, in a color scale between green

and blue between 1.5 and 35, purple in the case of 150 equivalents). In contrast to GalNAc and the Tn-antigen, which exhibit a slow-exchange regime on the NMR chemical-shift timescale, ligand 2 displays a fast-exchange regime. Moreover, V295 is only detected in the MGL-CRD bound form in the presence of more than 15 equivalents and L276 is only detected in the presence of 150 equivalents.

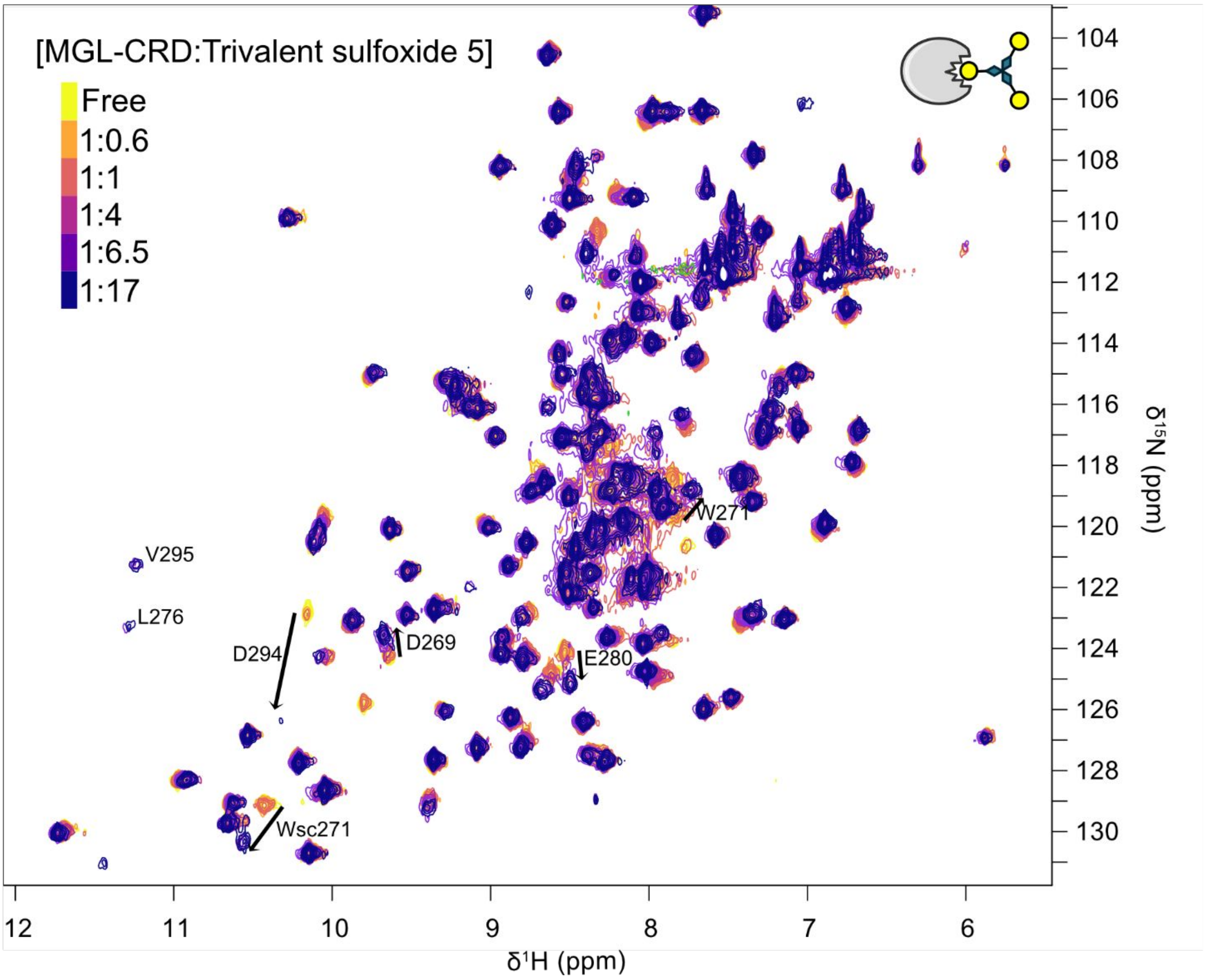

Figure S5. Region of 2D ^{1}H–^{15}N HSQC spectra of MGL-CRD/compound **5** complexes superimposed throughout the titration with the corresponding references: MGL-CRD apo (yellow) with respect to MGL-CRD in the presence of compound **5** (in 1:0.6, 1, 4, 6.5 and 17 molar ratio, in a color scale between orange and purple between 0.6 and 6.5, dark blue in the case of 17 equivalents) . In contrast to GalNAc and the Tn-antigen, which exhibit a slow-exchange regime on the NMR chemical-shift timescale, ligand **5** displays a fast-exchange/intermediate regime. Moreover, V295 is only detected in the MGL-CRD bound form in the presence of more than 4 equivalents and L276 is only detected in the presence of more than 6.5 equivalents.

Chemical Shift Perturbation (CSP): For the determination of Δδcomb it was used the equation:[4]

$$\Delta\delta \text{ comb} = \sqrt{(\Delta\delta\ ^{1}\text{H})^2 + (w_i \Delta\delta\ ^{15}\text{N})^2}$$

in which Δδcomb is the combined chemical shift observed, Δδ ^{1}H and Δδ ^{15}N are the chemical shifts of amide hydrogen and nitrogen atoms in ppm, respectively, between MGL-CRD apo and the MGL-CRD/compound **2** bound state in presence of compound **2** (in 1:150 molar ratio) or between the MGL-CRD apo and the MGL-CRD/compound **5** state in presence of compound **5** (in 1:17 molar ratio). w_i parameter is the ratio between the gyromagnetic constants of ^{15}N and ^{1}H. CSPs of residues D269 (A), W271sc (B), W271 (C) and Q267 (D) throughout the titration are shown as following (Figure S6 and S7).

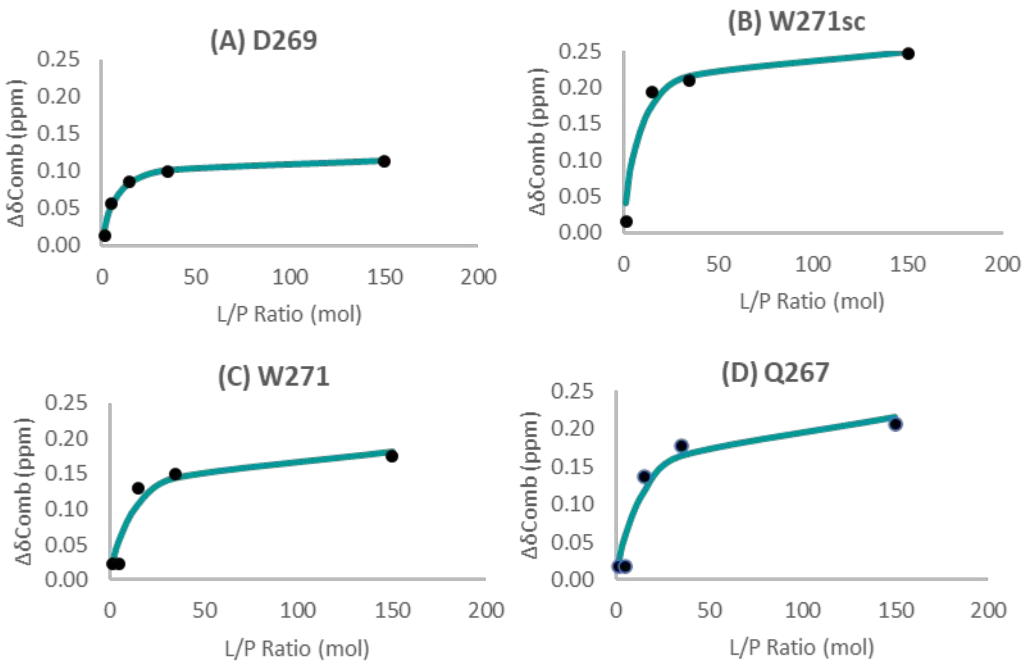

Figure S6. CSPs of residues Q267, D269, W271 and W271sc upon addition of compound **2**.

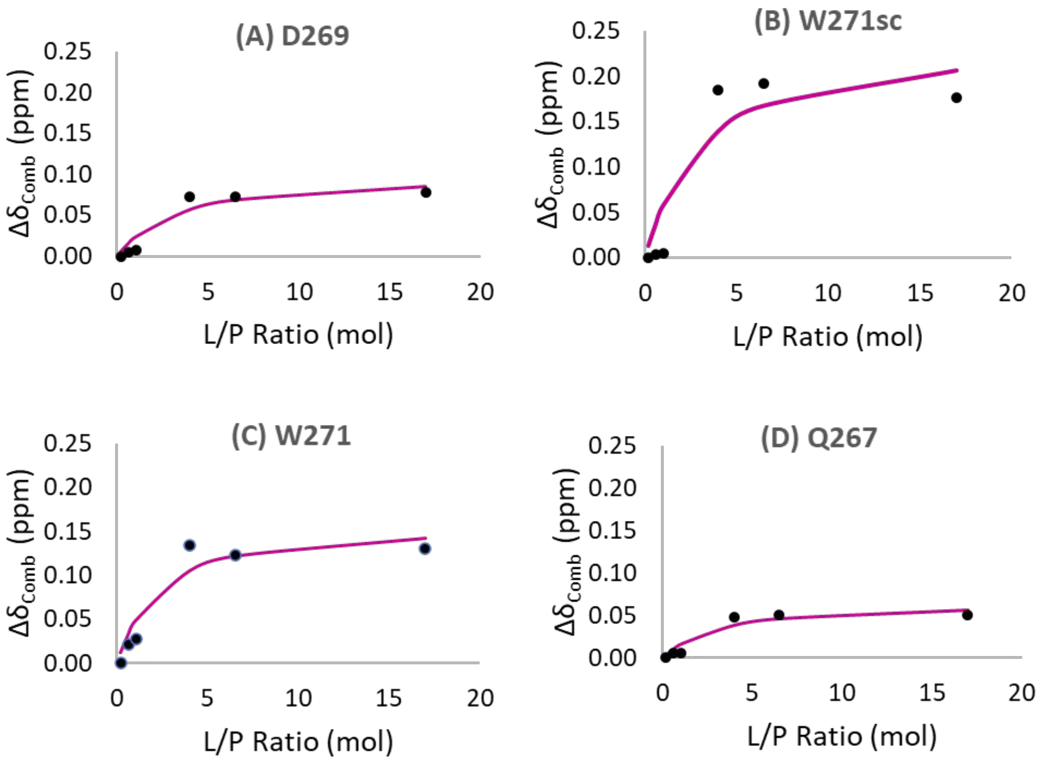

Figure S7. CSPs of residues Q267, D269, W271 and W271sc upon addition of compound **5**.

Estimation of the Dissociation Constant (K_D) by NMR

The apparent dissociation constants (K_D) were qualitatively deduced by the ^1H–^{15}N HSQC-based titration following the combined chemical shifts of the NH resonances of Y236, Q267, D269, W271, Wsc271 for the compound **2** and E280 and Q267, D269, W271, Wsc271 and E280 for the compound **5**, through the following equation[17]

$$\Delta\delta_{Comb} = \Delta\delta_{max}$$

$$(K_D+[L]_0+[P]_0)-\sqrt{(K_D+[L]_0+[P]_0)^2-4\times([L]_0\times[P]_0)}$$

Where $\Delta\delta_{comb}$ is the combined chemical shift deviation, $\Delta\delta_{max}$ is the maximum chemical shift deviation between free and bound state of protein, K_D is the dissociation constant, and $[P]_0$ and $[L]_0$ are the concentration of the protein and ligand, respectively. Finally, the average was calculated for each compound and the error was calculated through the standard deviation.

NMR comparison between compound 4 and 6:

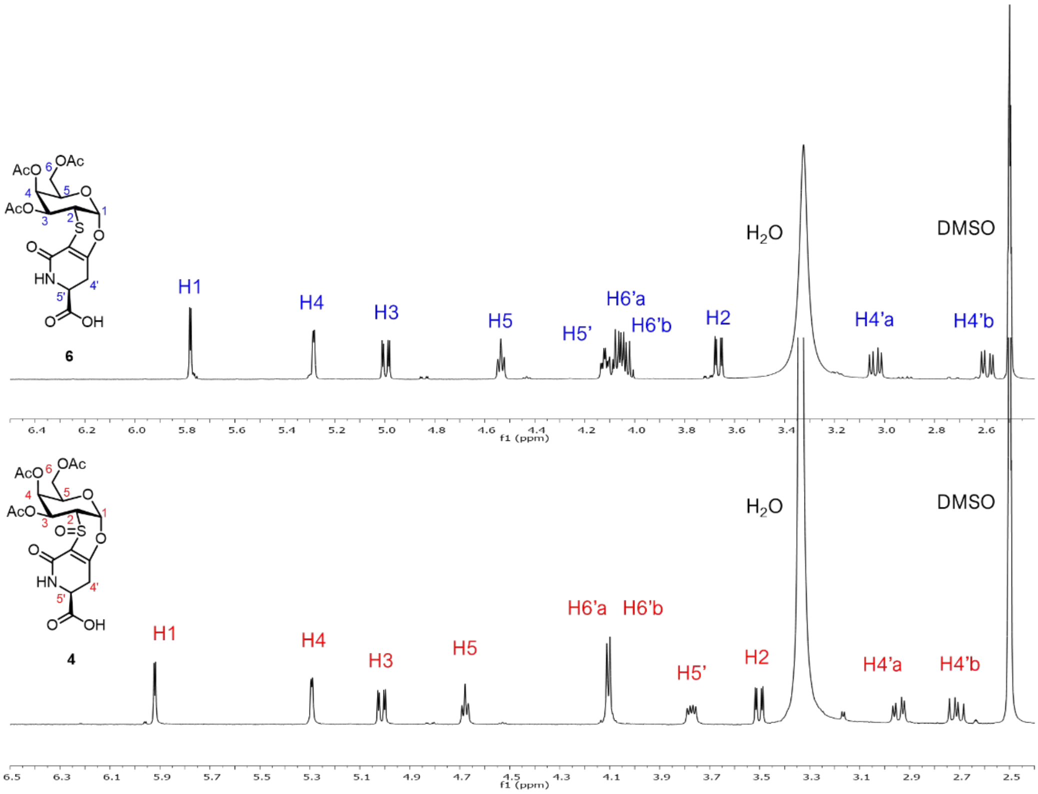

Figure S8. ^{1}H-NMR of compound **6** (above) and compound **4** (below), spectra recorded at 500 MHz and 25 °C in DMSO-d_6.

Table S1. Chemical shifts differences between compound **6** and **4.** Spectra recorded at 500 MHz and 25 °C in DMSO-d_6.

Proton	Compound 6 δ (ppm)	Compound 4 δ (ppm)	Δδ (ppm)
**H1**	5,78	5,92	0,14
**H2**	3,66	3,5	-0,16
**H3**	5,00	5,01	0,01
**H4**	5,28	5,29	0,01
**H5**	4,54	4,68	0,14
**H6a**	4,07	4,11	0,04
**H6b**	4,04	4,11	0,07
**H4'a**	3,04	2,95	-0,09
**H4'b**	2,59	2,71	0,12
**H5'**	4,12	3,77	-0,35

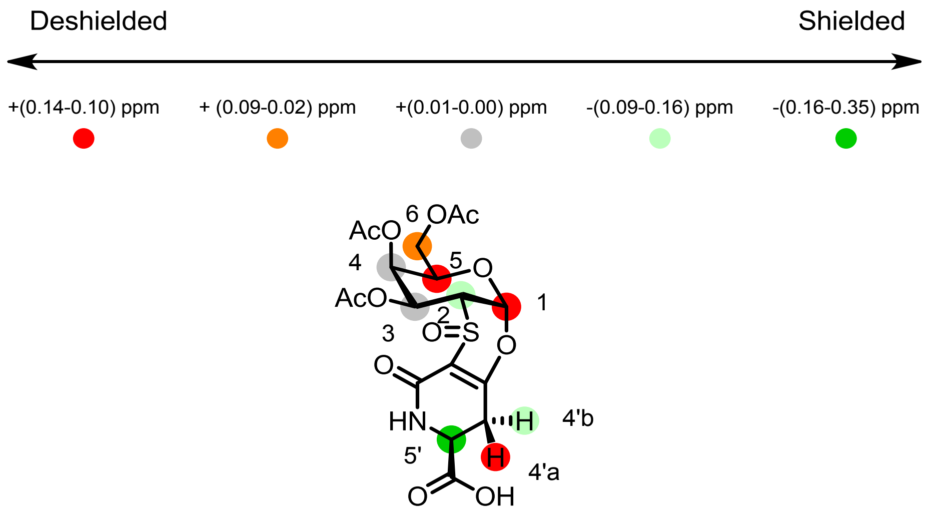

4

Figure S9. Schematic representation of the chemical shift perturbations observed between compound **6** and compound **4**. Residues highlighted in red indicate regions exhibiting the greatest deshielding, orange denotes moderate deshielding, grey corresponds to little or no chemical shift change, light green indicates moderate shielding, and green represents strong shielding effects.

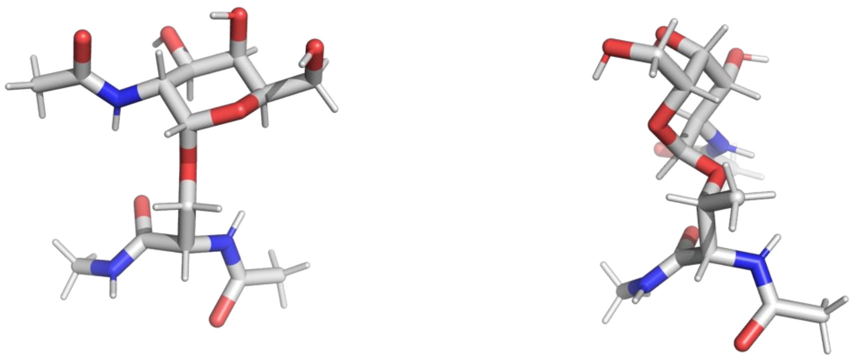

Figure S10. 3D structure of antigen Tn-Ser (left) and Tn-The (right) which highlight the different orientation of the GalNAc pyranose with respect to the peptide chain (for simplicity, only the first amino acid is here represented). For detail see ref.[5,6]

Inhibition of ST6GALNAC1

Asialo bovine submaxillary mucin (aBSM) to be used as ST6GALNAC1 acceptor substrate, was obtained by subjecting bovine submaxillary mucin (Sigma) to mild acid hydrolysis (50 mM H_2SO_4 80 °C 1 h), followed by extensive dialysis against water and drying. The ST6GALNAC1 assay mixture contained in a final volume of 50 µL: 25 mM Tris/HCl buffer pH 7.5, 10 mM $MnCl_2$, 1.2 x 10^4 Bq CMP-[^{3}H]Sialic acid (0.55 µM) (American Radiolabeled Chemicals), 300 µg aBSM, 0.15 µg recombinant ST6GALNAC1 (R&D systems) and variable amounts of inhibitors (from 0 to 9 mM). Samples were incubated for 2 h at 37 °C. Samples without enzyme were incubated in parallel as blanks. The acid insoluble radioactivity incorporated on aBSM was measured after precipitation with 1% phosphotungstic acid in 0.5 M HCl, followed by three washings with 1% phosphotungstic acid in 0.5 M HCl. Samples were then solubilized by boiling in 0.5 M HCl, transferred in liquid scintillation vials and, after addition of 3 mL of liquid scintillation cocktail (Beckman Coulter), counted in a liquid scintillation counter. The incorporation of the samples without enzyme was subtracted. The IC50 concentration of the two compounds was calculated as reported.[18]

We have roughly estimated the acceptor site concentration in the assay as follows. The sialic acid (Sia) content of our BSM preparation was 25% by weight (from the data sheet). According to a recently published paper,[7] about 25% of the BSM sialylated chains are represented by the disaccharide Sia-GalNAc, i.e the sialyl-Tn antigen. No di- or trisialylated chains were reported. On this basis, we calculated that in BSM the Sia (MW=309) concentration bound to sialyl-Tn was about 0.2 µmol/mg BSM. This is also the rough concentration of terminal GalNAc residues after de-sialylation. Consequently, in the 300 µg of asialo-BSM in a reaction volume of 50 µL, the concentration of the acceptor sites of ST6GALNAC1 is about 1.2 mM.

1D and 2D NMR Spectra

Compound 2 (Fugure S11)

^{1}H NMR (500 MHz in D_2O- 25 deg)

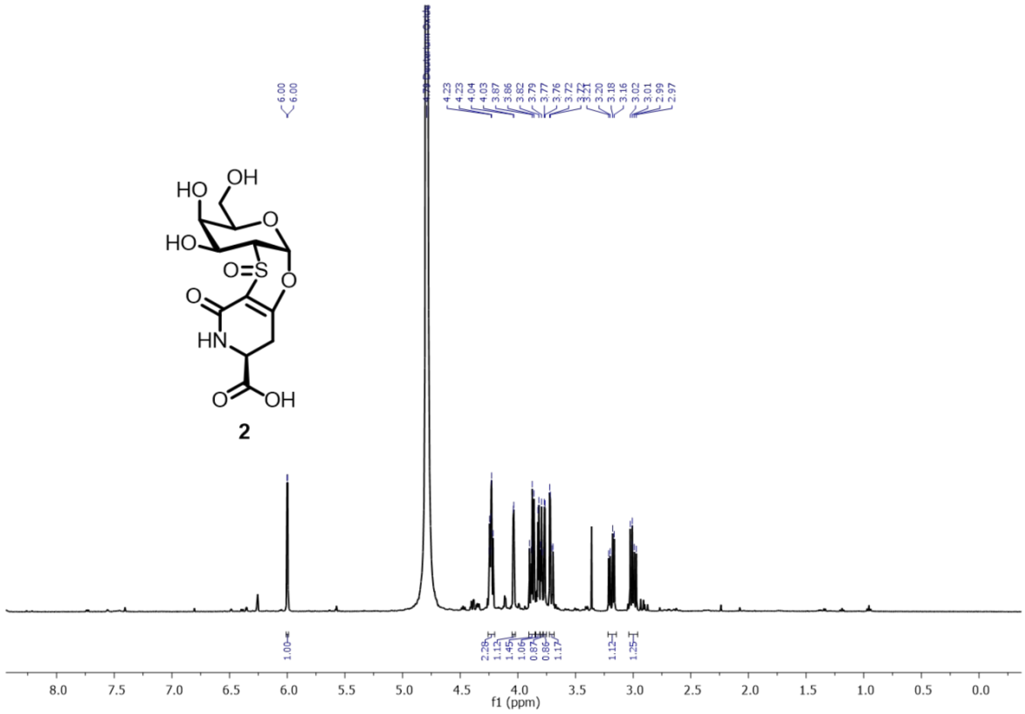

^{13}C NMR (125 MHz in D_2O - 25 deg)

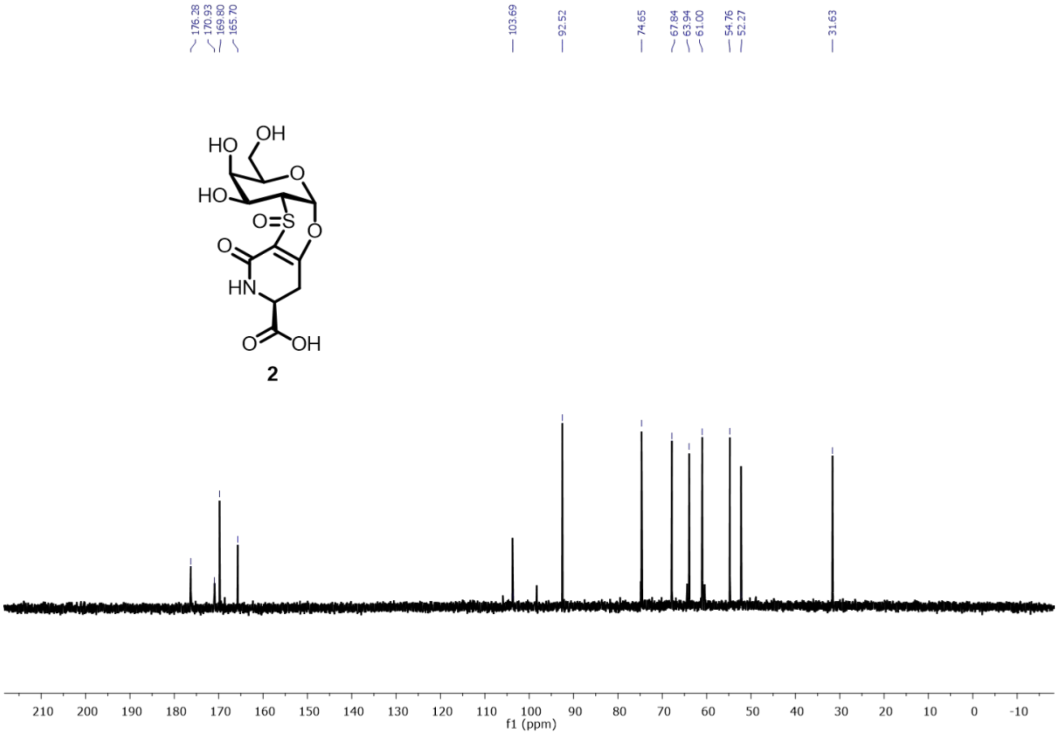

2D COSY (500 MHz in D_2O - 25 deg)

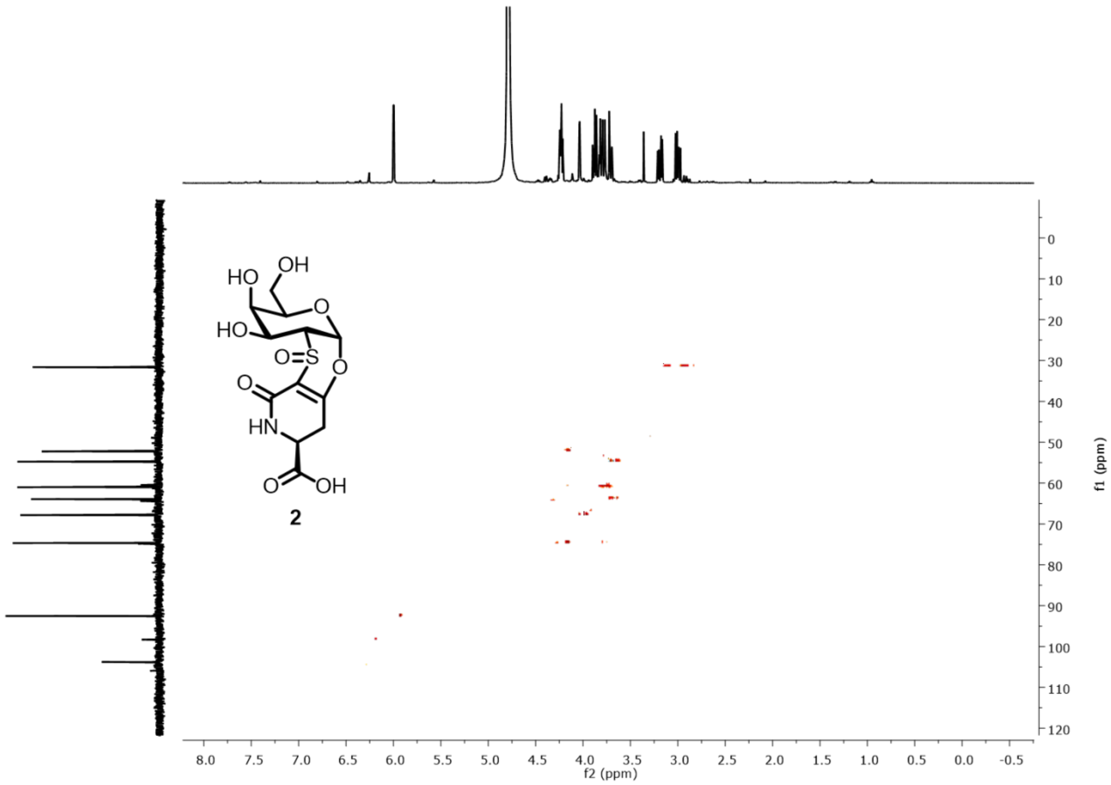

^1H-^{13}C HSQC (500 MHz in D_2O - 25 deg)

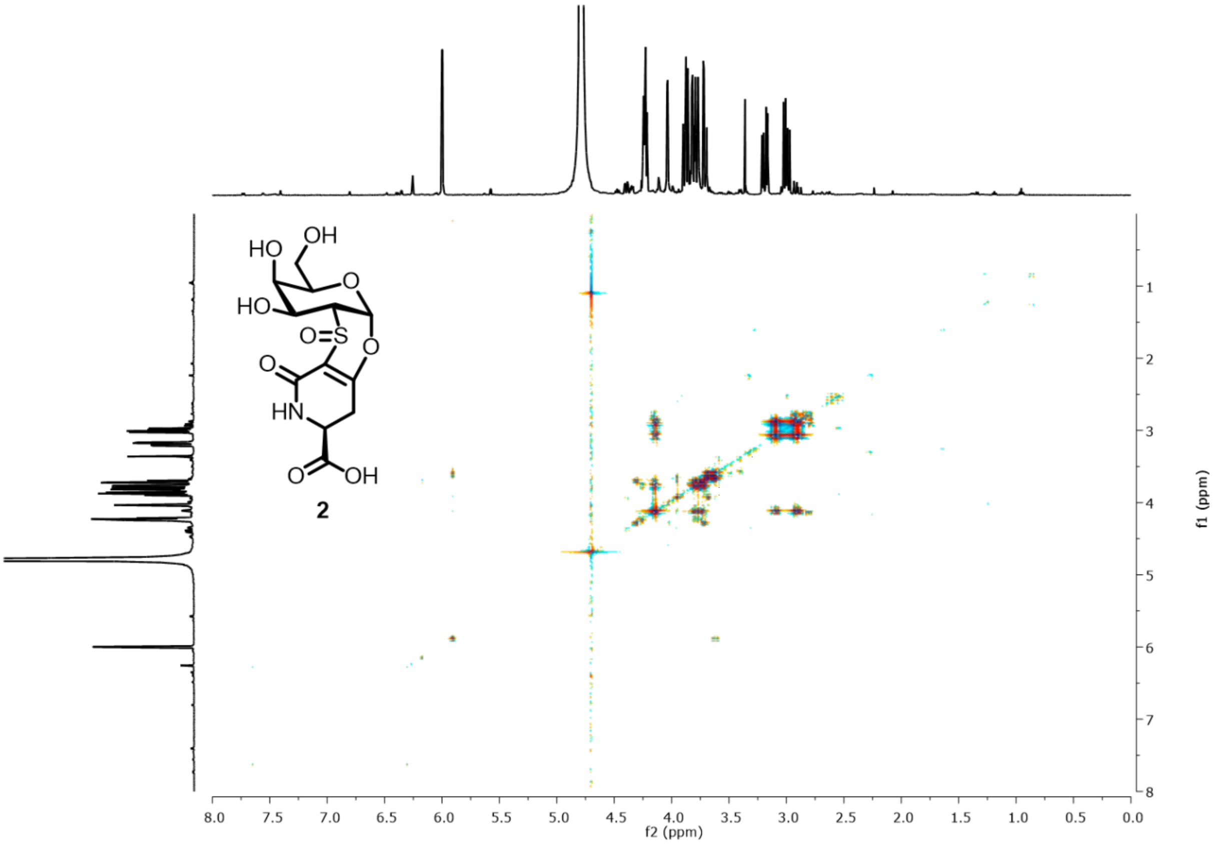

Compound 3 (Figure S12)

^{1}H NMR (500 MHz in $CDCl_3$ - 25 deg)

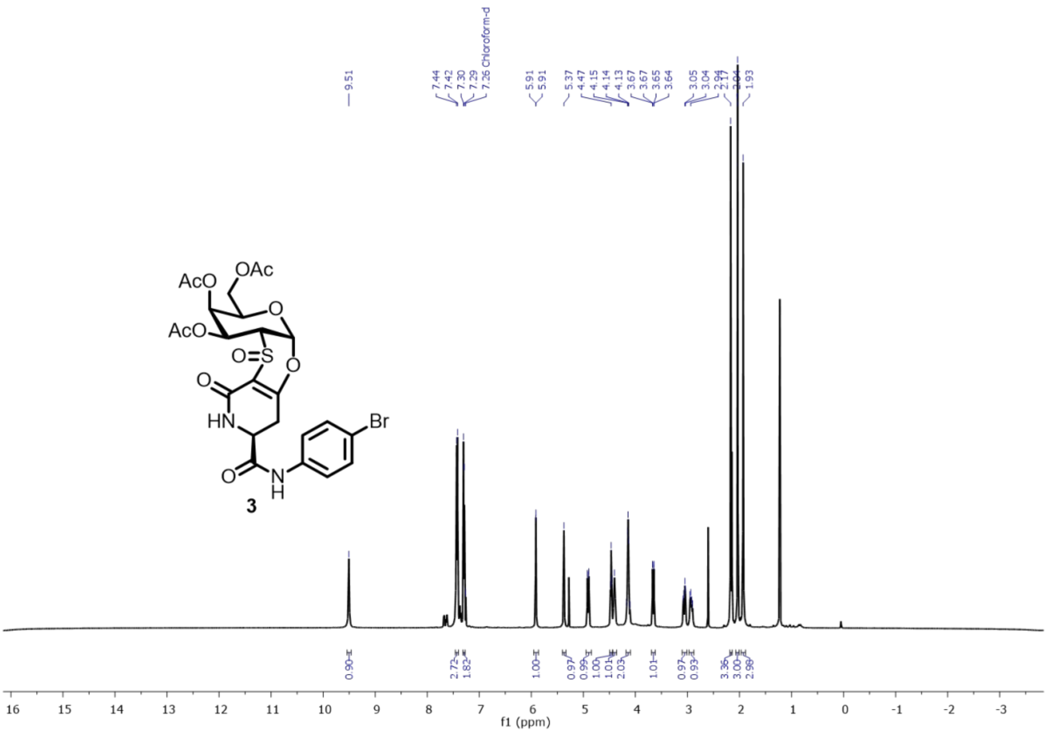

^{13}C NMR (125 MHz in $CDCl_3$ - 25 deg)

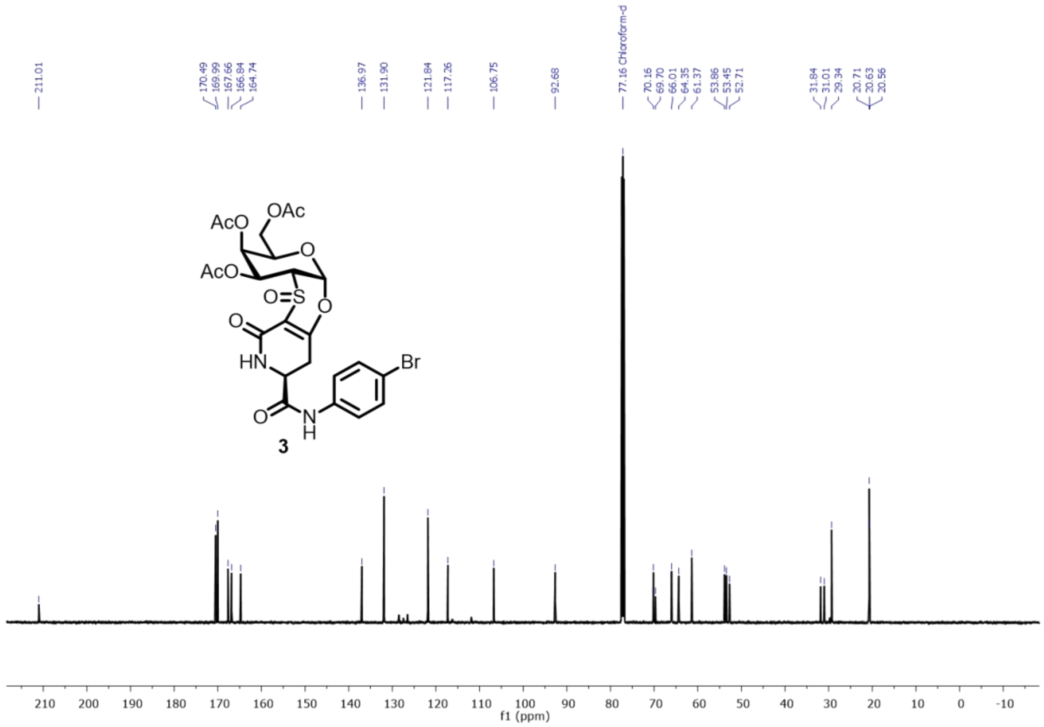

2D COSY (500 MHz in $CDCl_3$ - 25 deg)

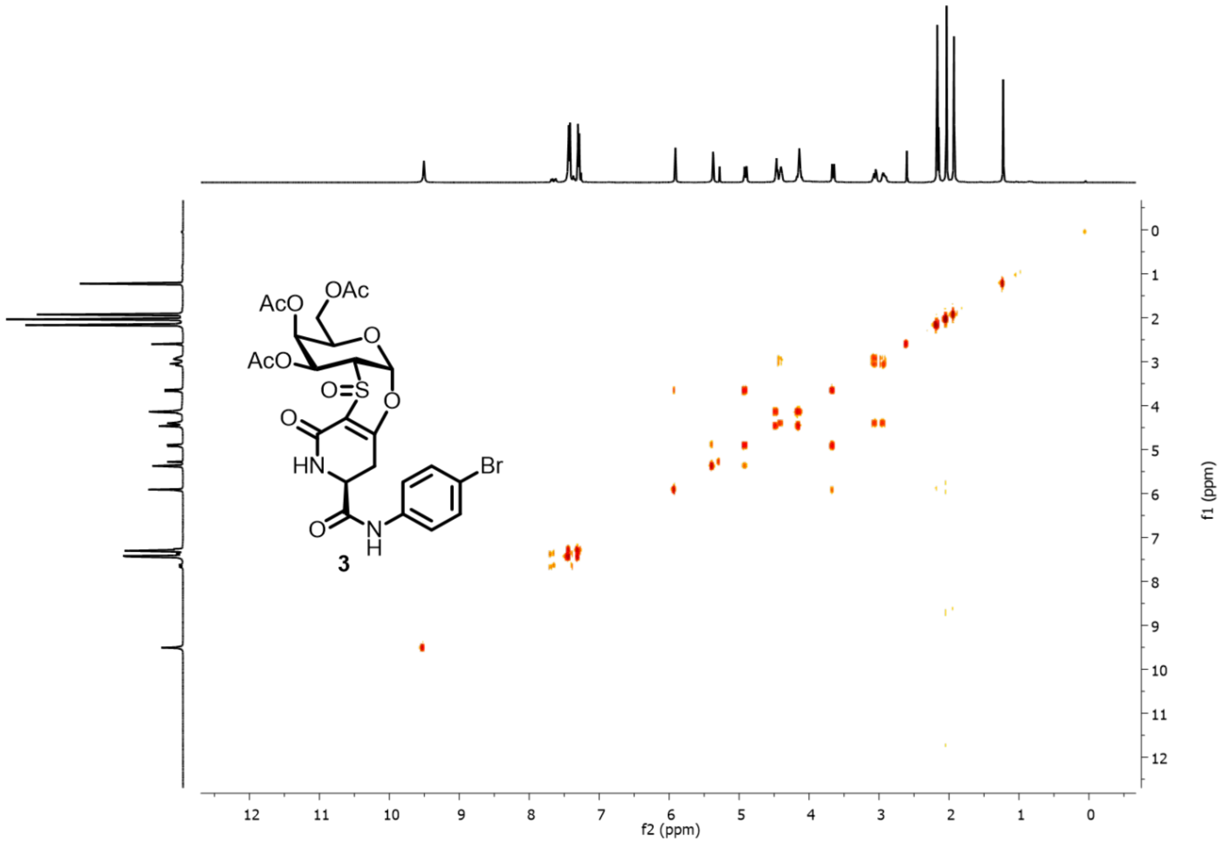

^{1}H-^{13}C HSQC (500 MHz in $CDCl_3$ - 25 deg)

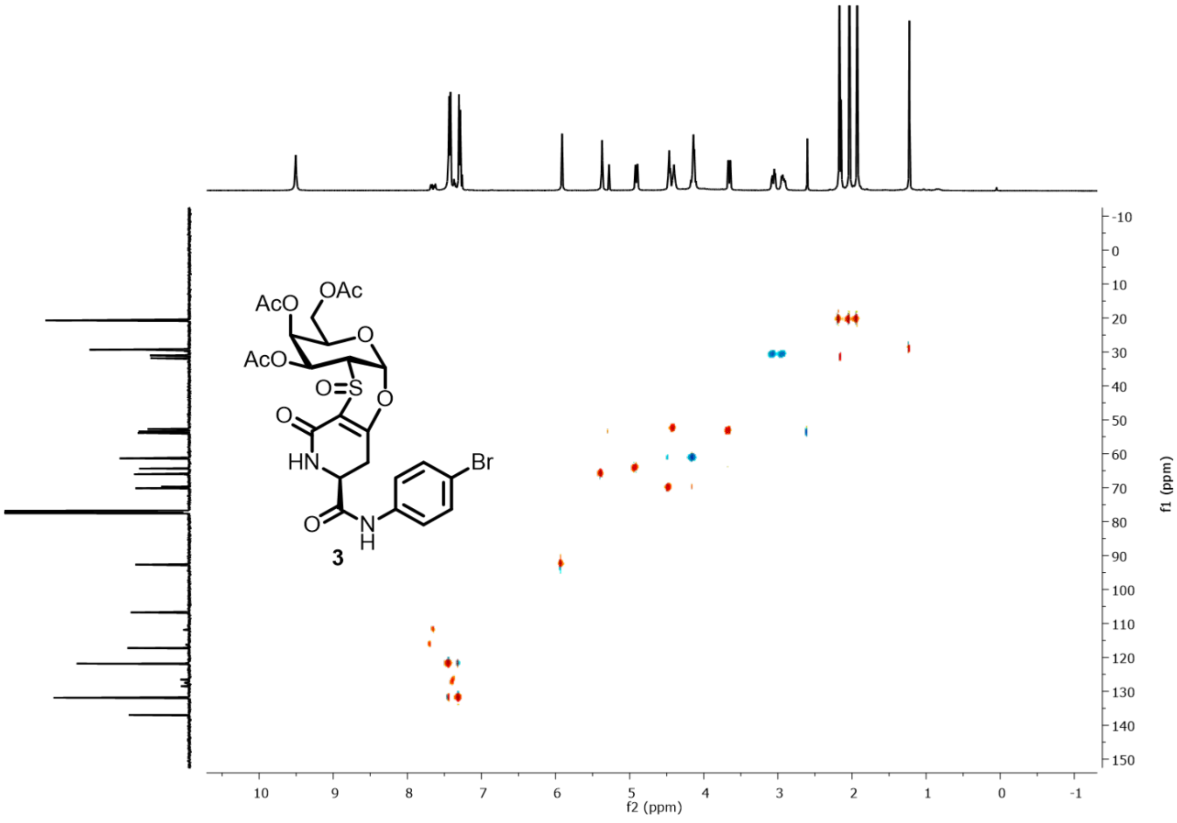

Compound 4 (Figure S13)

^{1}H NMR (500 MHz in DMSO-d_6 - 25 deg)

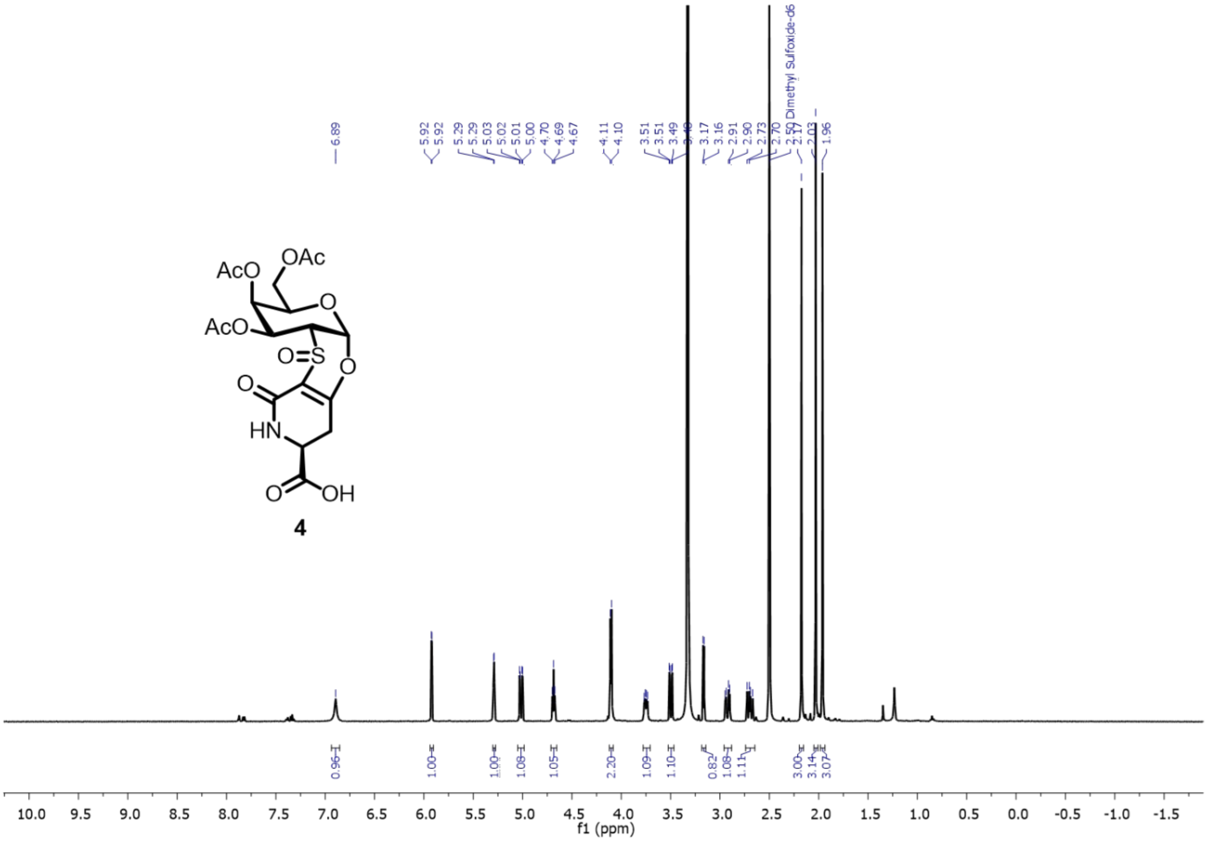

^{13}C NMR (125 MHz in DMSO-d_6 - 25 deg

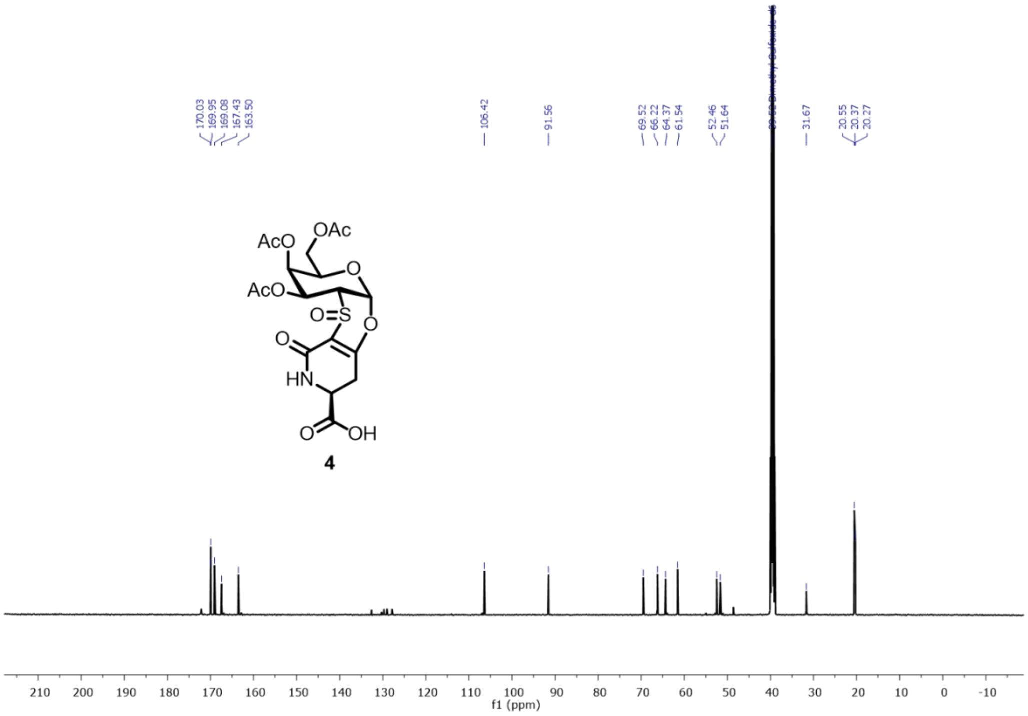

2D COSY (500 MHz in DMSO-d_6 - 25 deg)

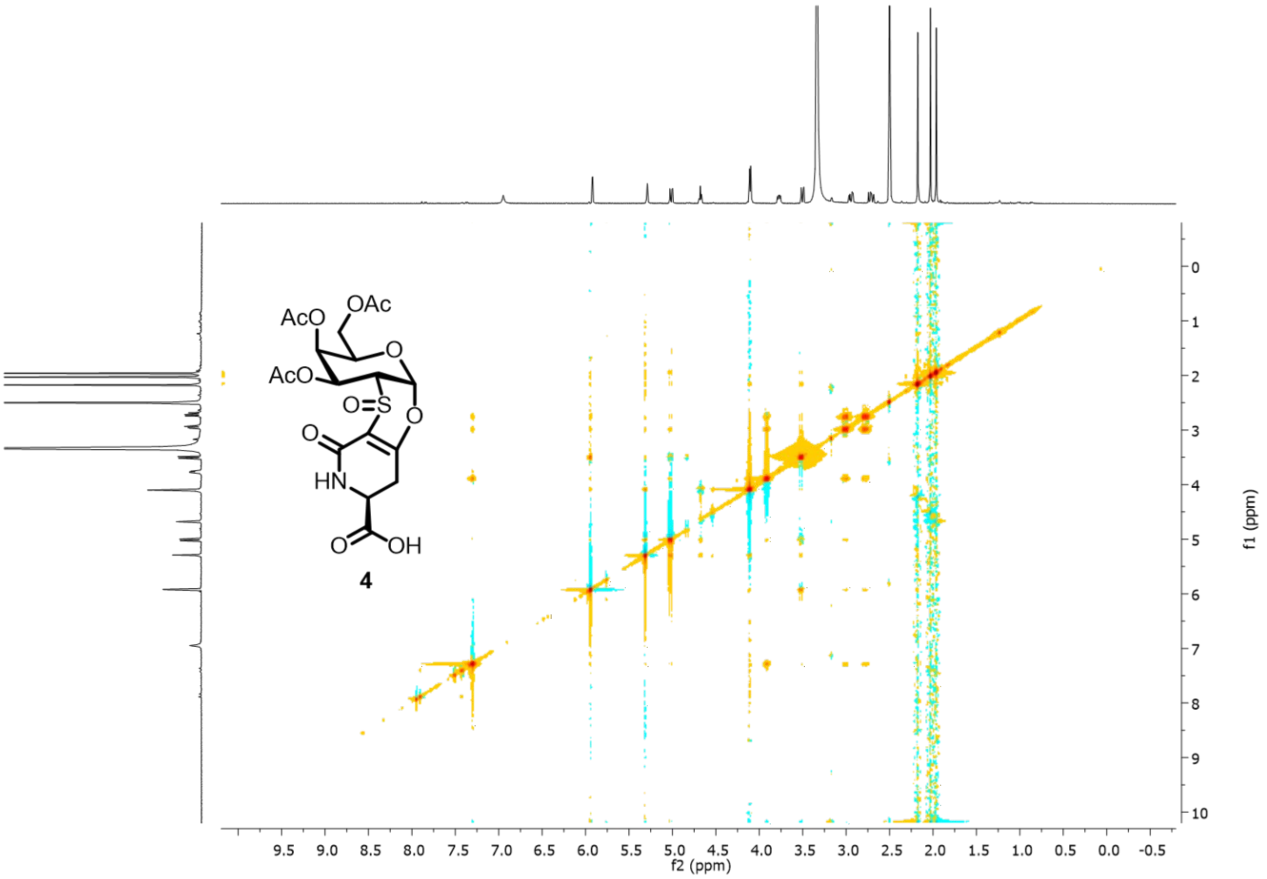

^1H-^{13}C HSQC (500 MHz in DMSO-d_6 - 25 deg)

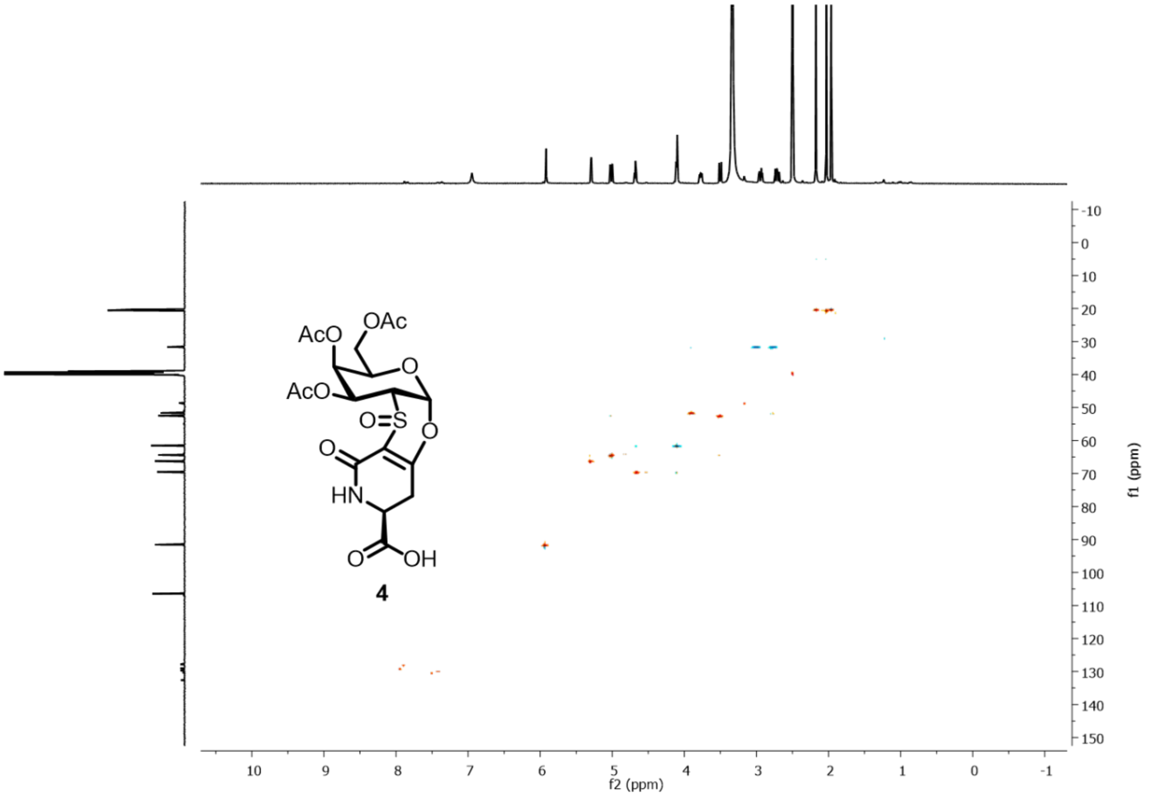

Compound 5 (Figure S14)

^{1}H NMR (500 MHz in D_2O- 25 deg)

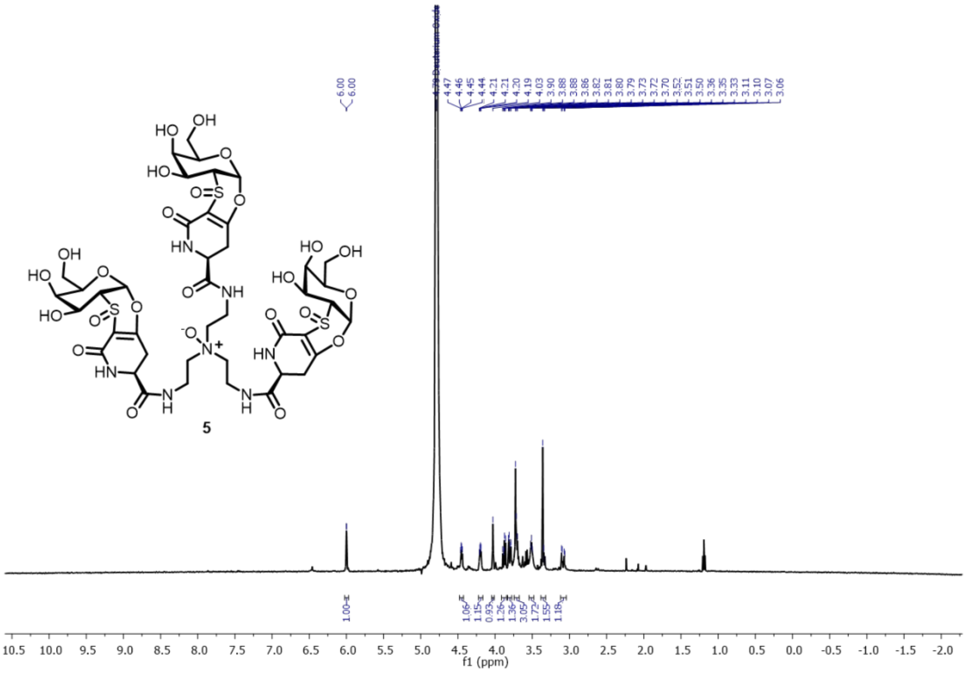

^{13}C NMR (125 MHz in D_2O - 25 deg)

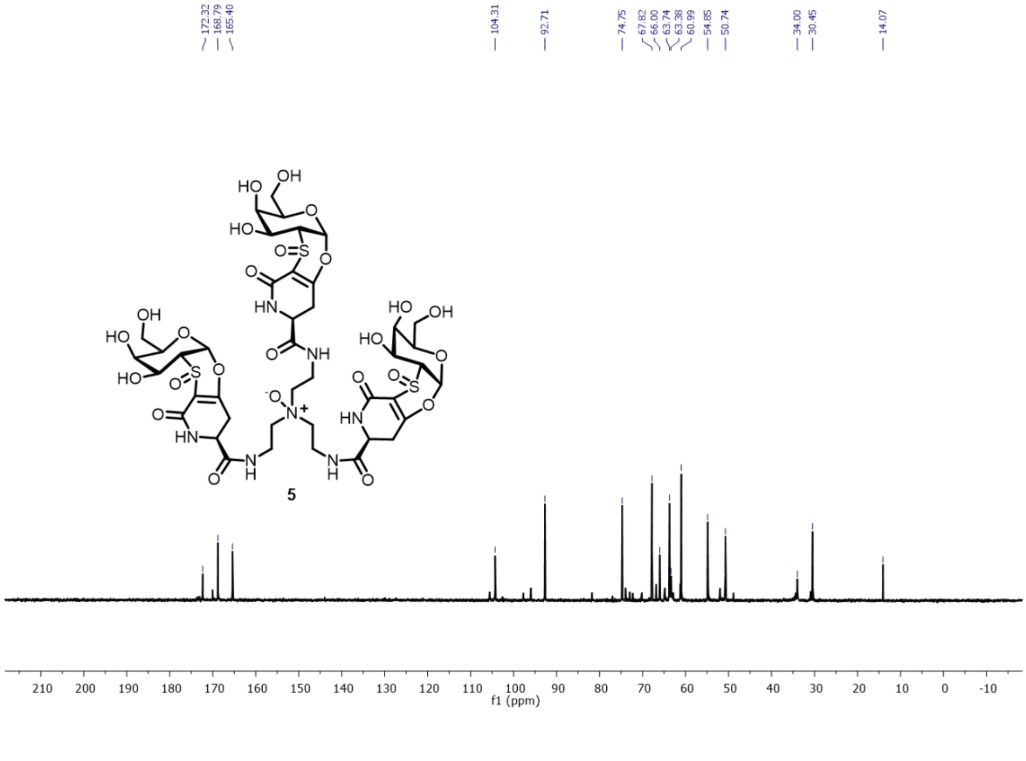

2D COSY (500 MHz in D_2O - 25 deg)

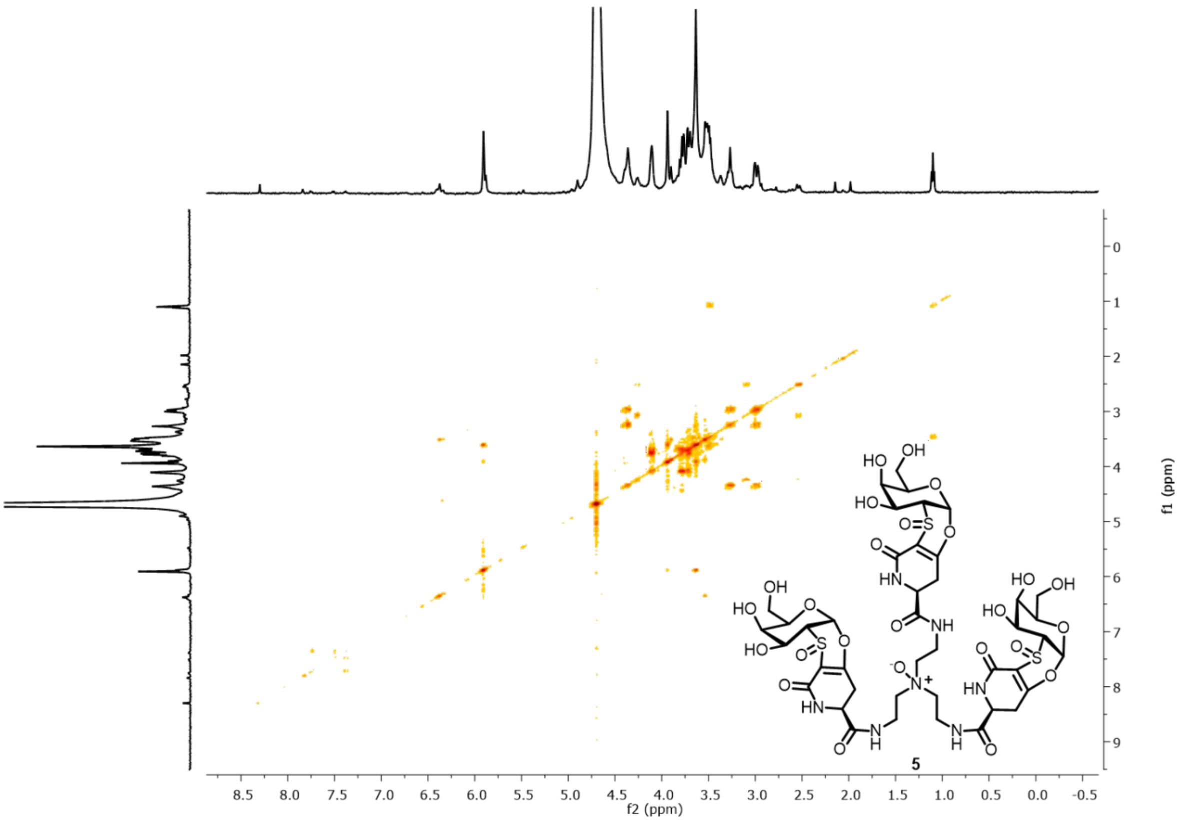

^{1}H-^{13}C HSQC (500 MHz in D_2O - 25 deg)

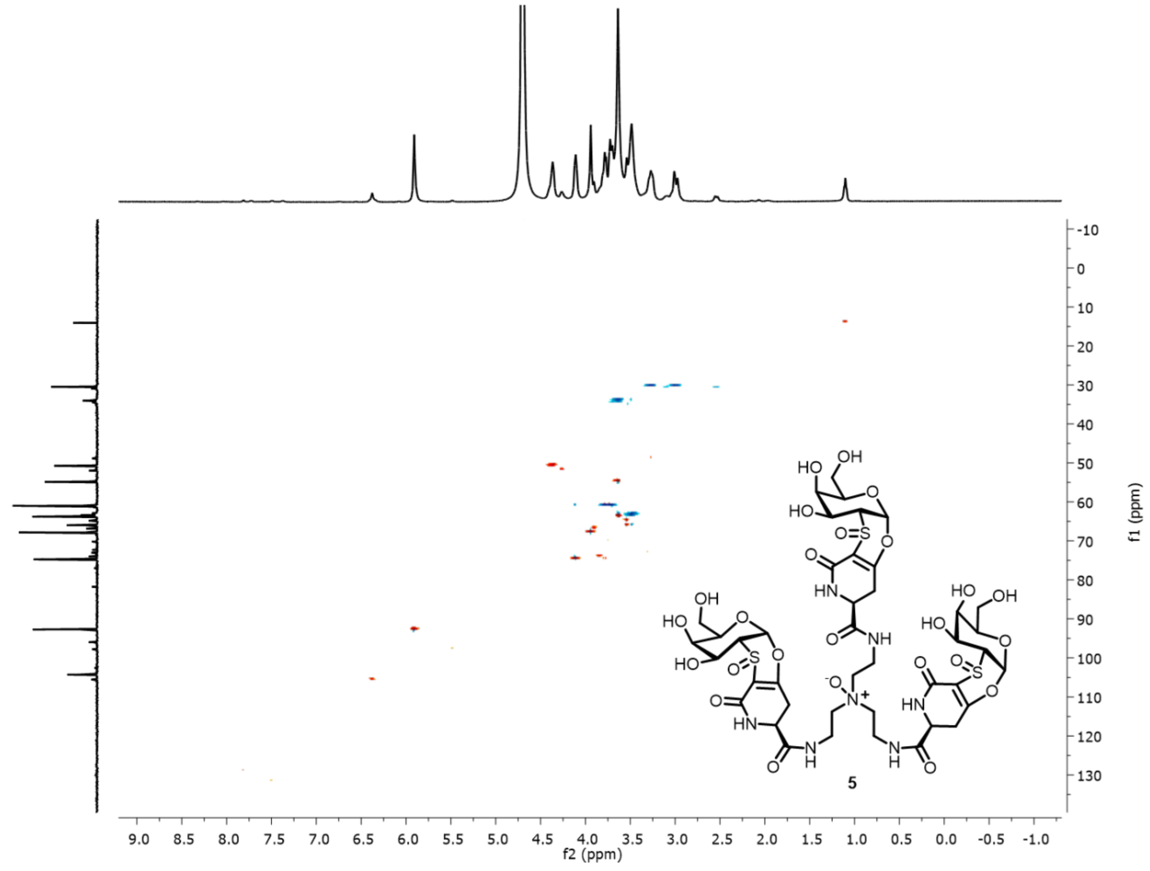

Compound 7 (Figure S15)

^{1}H NMR (500 MHz in $CDCl_3$ - 25 deg)

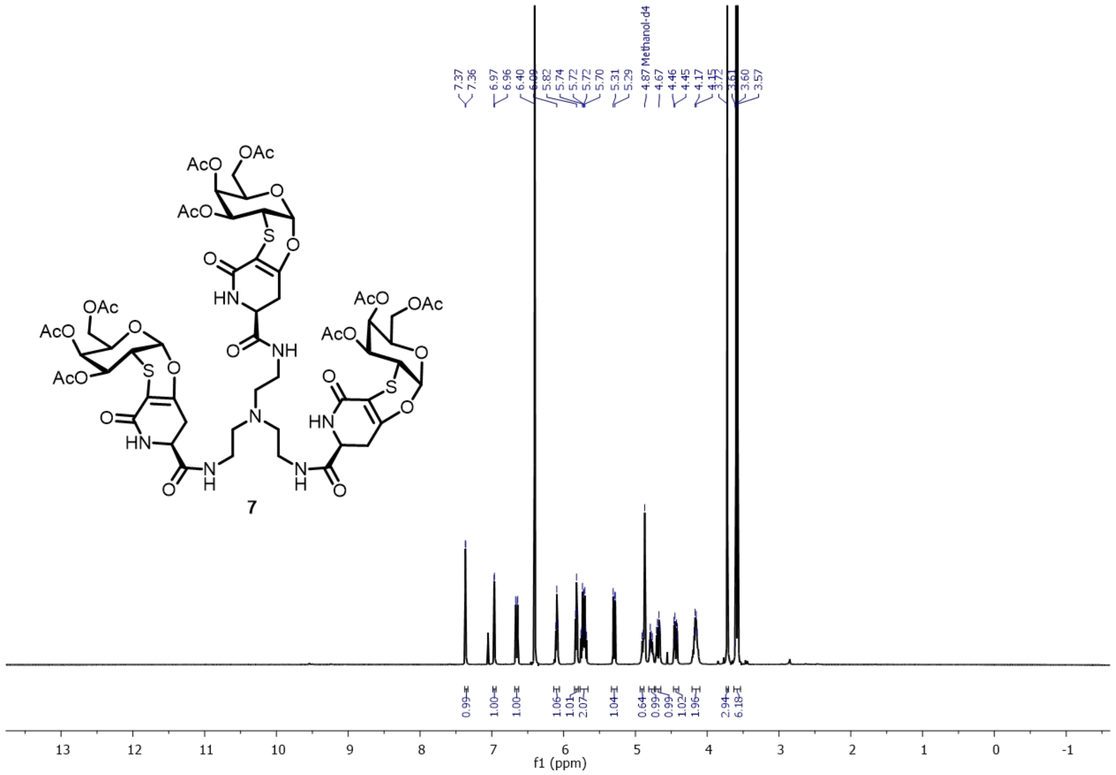

^{13}C NMR (125 MHz in $CDCl_3$ - 25 deg)

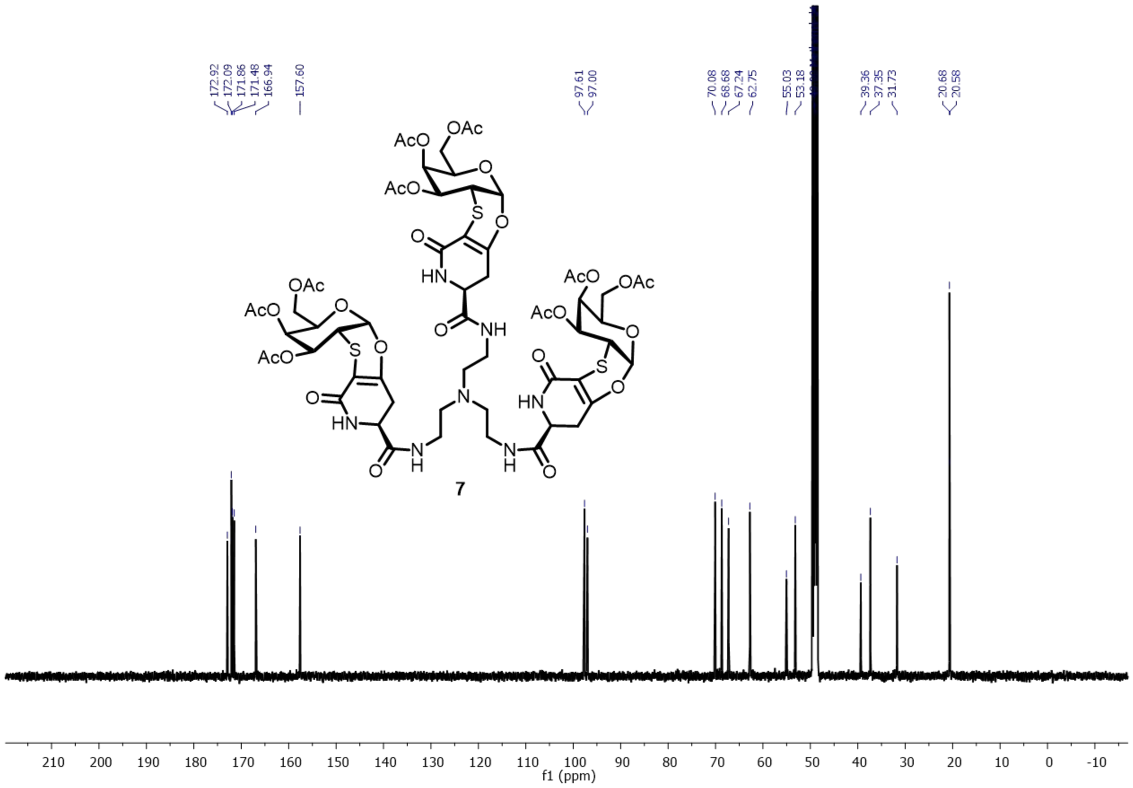

2D COSY (500 MHz in $CDCl_3$ - 25 deg)

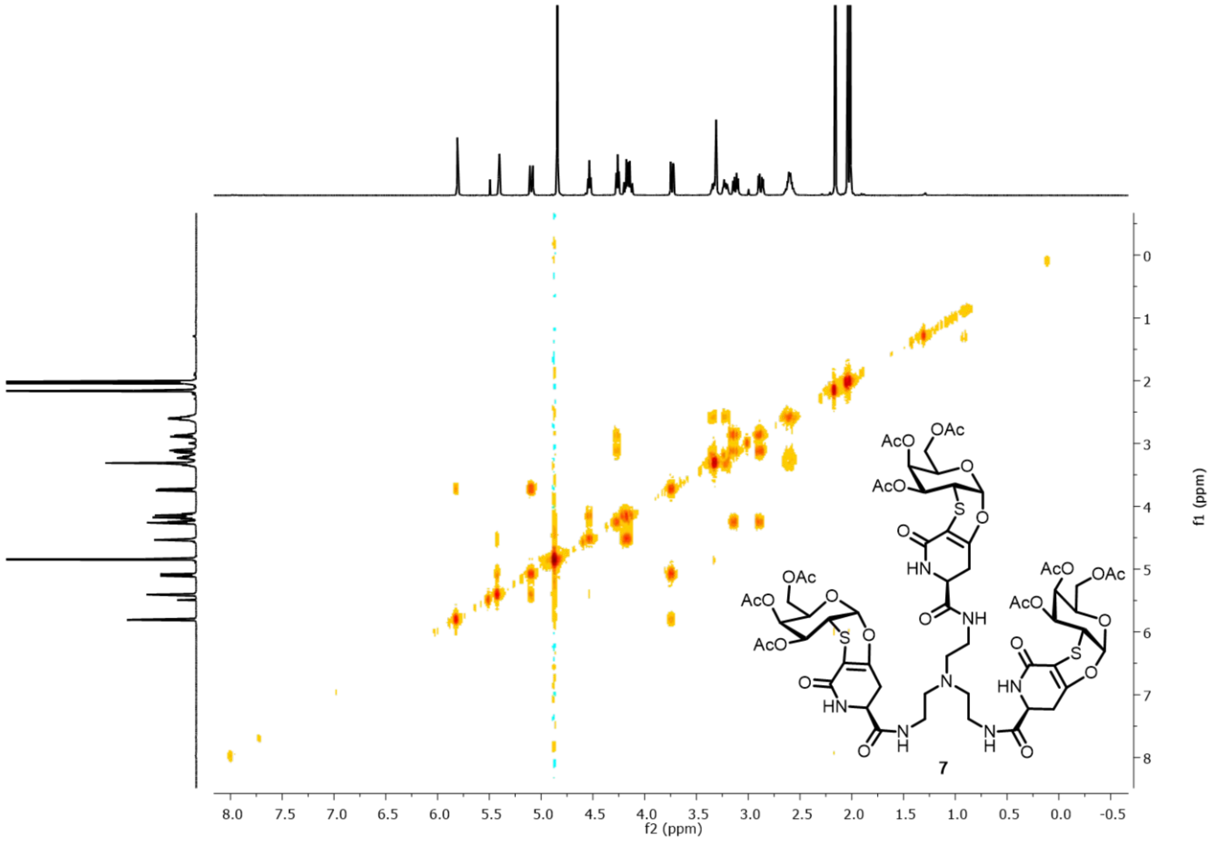

^1H-^{13}C HSQC (500 MHz in $CDCl_3$ - 25 deg)

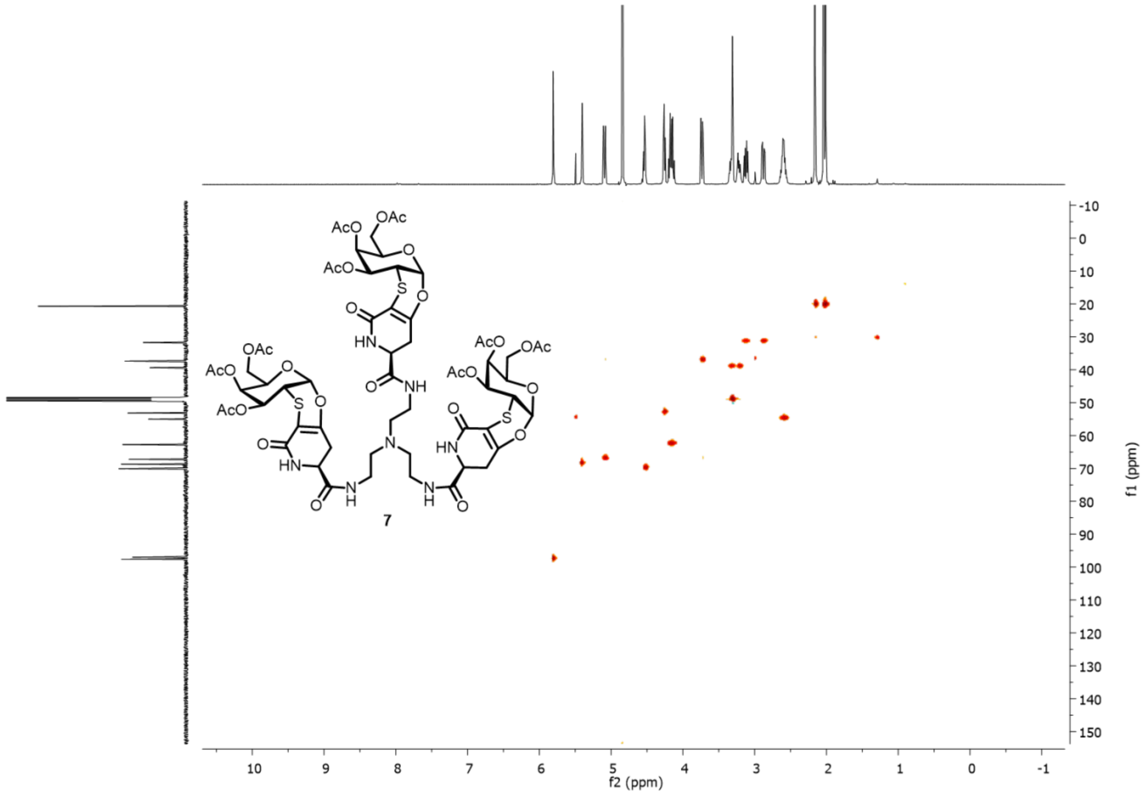

Compound 8 (Figure S16)

^{1}H NMR (500 MHz in D_2O- 25 deg)

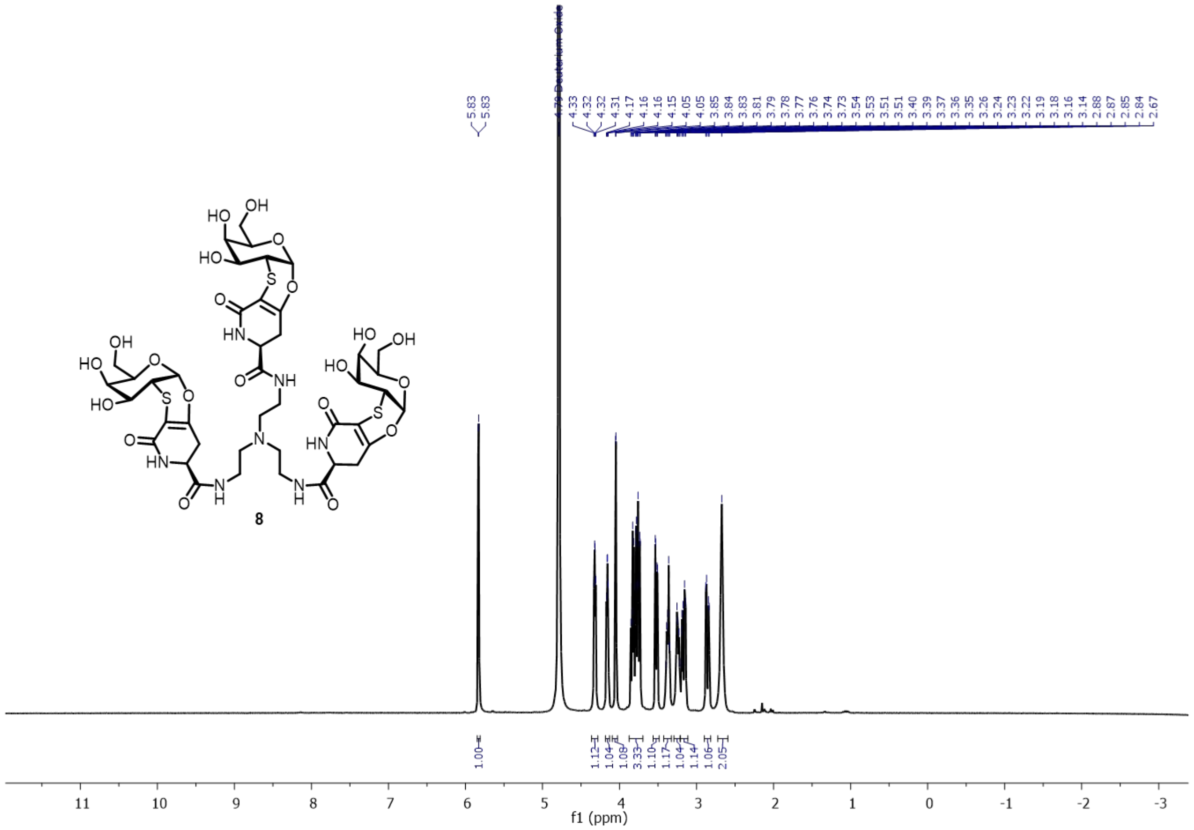

^{13}C NMR (125 MHz in D_2O - 25 deg)

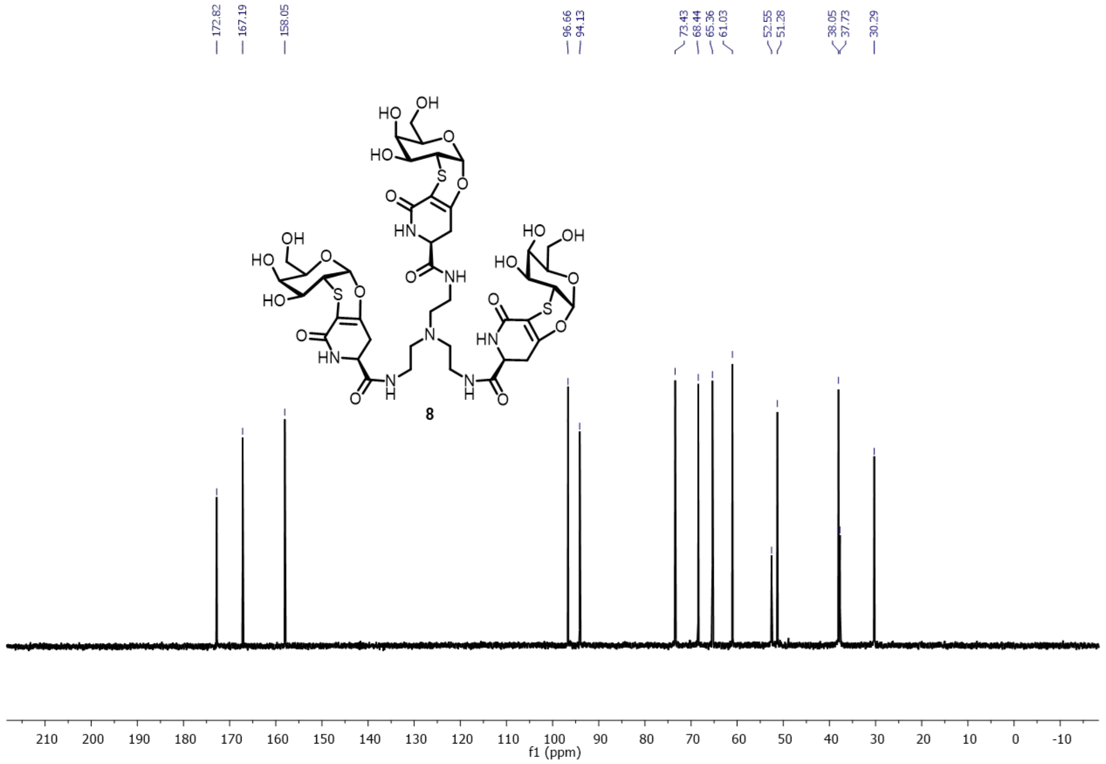

2D COSY (500 MHz in D_2O - 25 deg)

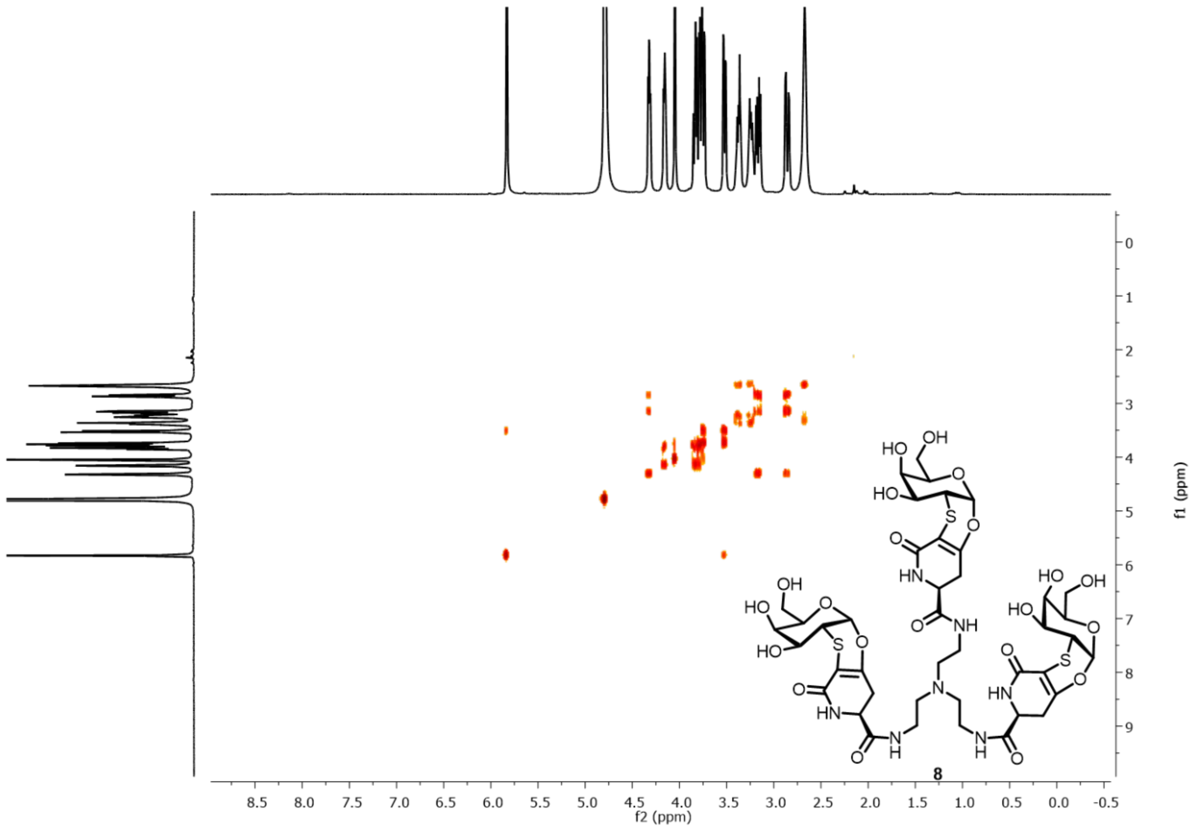

^1H-^{13}C HSQC (500 MHz in D_2O - 25 deg)

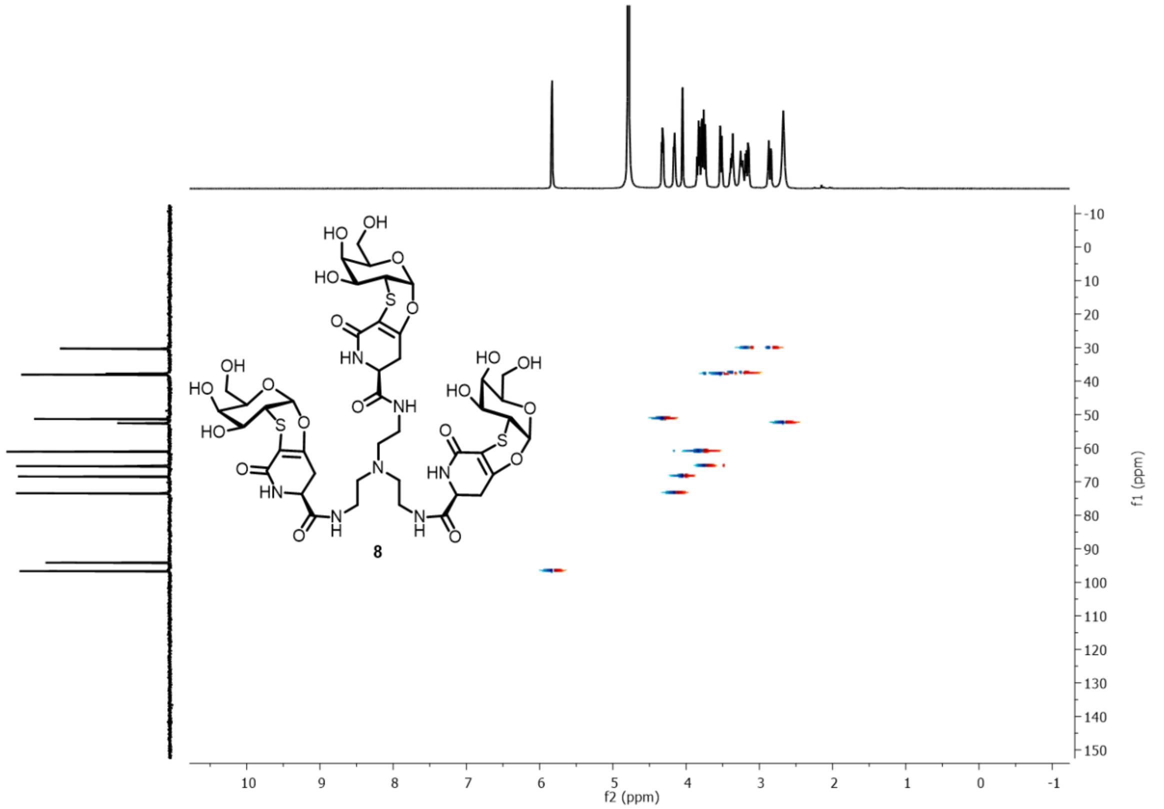

Compound 9 (Figure S17)

^{1}H NMR (500 MHz in D_2O- 25 deg)

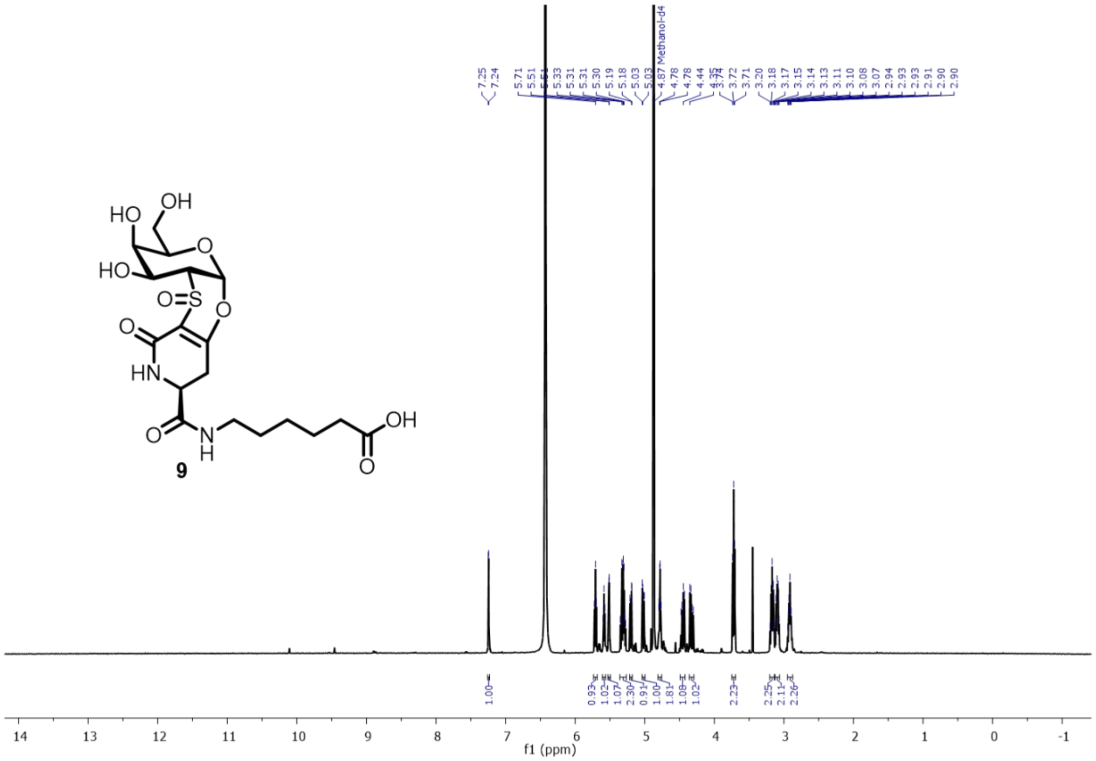

^{13}C NMR (125 MHz in D_2O - 25 deg)

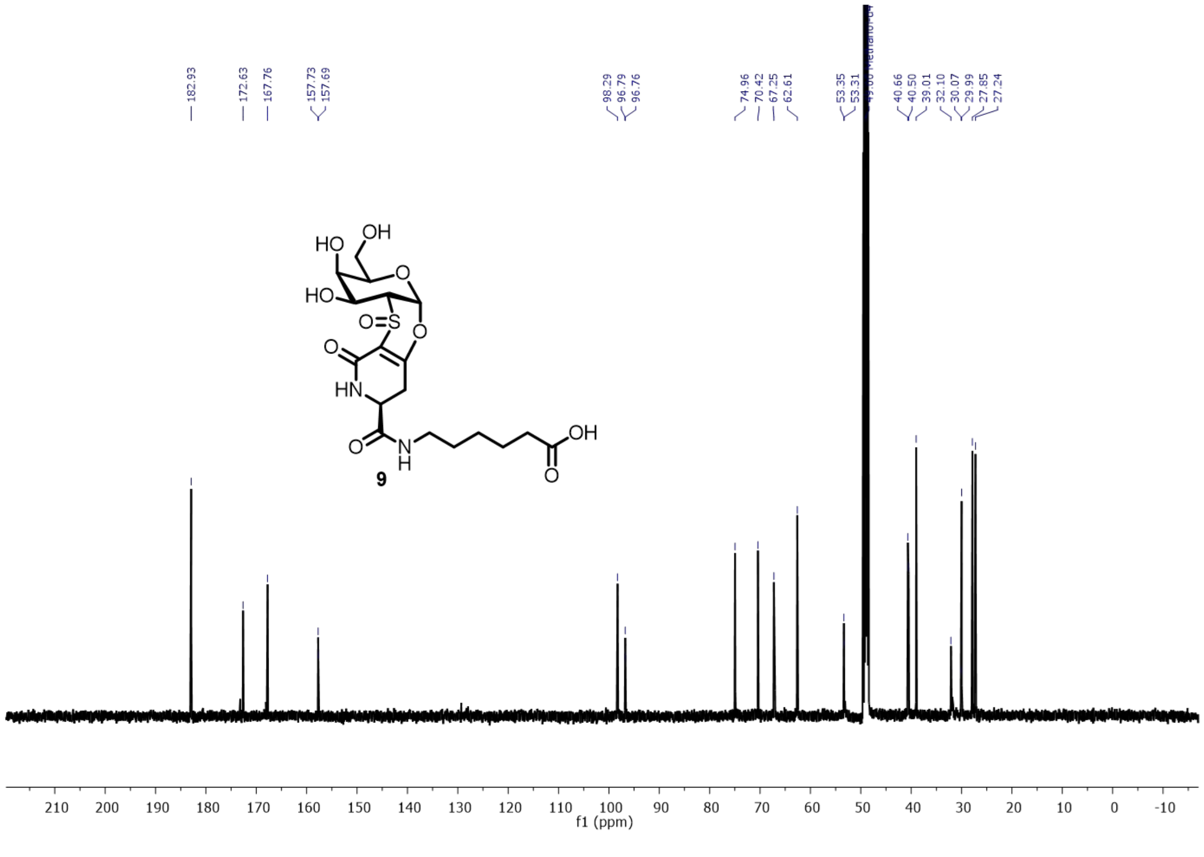

Cartesian coordinates

1
PCM(H_2O)-B3LYP/6-31g(d),
el. energy = -1521.041057 a.u.

C	-1.94892500	1.24709600	0.64318800
C	-1.26636600	0.94497300	-0.70780000
C	-1.02358700	-0.55924100	-0.89621800
C	-2.85028500	-1.12394100	0.54656200
C	-3.19477900	0.35251700	0.78339500
H	-0.66635500	-0.77566600	-1.90525300
H	-1.94705700	1.25069700	-1.50705900
H	-1.25738600	1.04559200	1.46725400
H	-2.19402500	-1.45956600	1.35971200
H	-3.57964300	0.44746600	1.81103900
O	-2.17992100	-1.31261900	-0.71935100
O	-0.05297100	-1.08703700	0.04208000
O	-2.32295100	2.60885000	0.75102100
H	-3.12563500	2.70049100	0.20540200
O	-4.14293700	0.86988300	-0.14759100
H	-4.73048500	0.13040900	-0.41060100
C	-4.06619000	-2.04594800	0.49646700
H	-4.63907500	-1.95320500	1.42260900
H	-3.73366200	-3.08574000	0.39463700
O	-4.95954200	-1.70008700	-0.57185600
H	-4.47229800	-1.84981700	-1.40066700
C	1.08527100	-0.40981200	0.31502600
C	1.37889100	0.84980800	-0.08319000
C	2.01448600	-1.18613500	1.20953700
C	2.66199200	1.48388300	0.28969000
C	3.46932500	-0.74068000	0.99311800
H	1.73739400	-1.02092200	2.25863200
H	4.47493000	1.11387400	1.06215600
S	0.27317500	1.89218100	-1.00586800
O	2.91452400	2.65716900	0.00668200
N	3.54781900	0.70781700	1.00311800
H	4.07643000	-1.12989400	1.81621500
H	1.91333400	-2.25469100	1.00794800
C	4.06303600	-1.32019600	-0.30427700
O	4.49639300	-0.66498400	-1.22636000
O	4.06502300	-2.66612100	-0.27380500
H	4.45985300	-2.98303300	-1.10951500

mCPBA
PCM(H_2O)-B3LYP/6-31g(d),
el. energy = -955.547402 a.u.

O	3.62003700	-1.43905600	0.00009500
H	3.97973400	-0.51476900	0.00004700
O	2.20538200	-1.16438500	0.00132200
C	1.96490900	0.17350600	-0.00018800
O	2.88159200	0.97885700	-0.00153100
C	0.51884000	0.49071100	0.00018600
C	0.14988700	1.84270600	0.00029500
C	-0.45904600	-0.51547500	-0.00003000
C	-1.19854300	2.19019600	0.00047300
H	0.92176300	2.60389900	0.00031700
C	-1.79826700	-0.14123900	0.00005900
H	-0.18248200	-1.56251700	-0.00044700
C	-2.18359300	1.20056800	0.00033500
H	-1.49046700	3.23542400	0.00057200
H	-3.23527200	1.46551000	0.00031800
Cl	-3.03614700	-1.39274800	-0.00039300

VdW adduct-*S*
PCM(H_2O)-B3LYP/6-31g(d),
el. energy = -2476.591510 a.u.

C	2.44427400	2.83599500	-0.52222900
C	1.47954500	1.75400700	-1.05051100
C	0.77827300	1.00773600	0.09413300
C	1.00292700	2.88392100	1.56707200
C	1.69280300	3.72631000	0.48546100
H	0.00293500	0.34293000	-0.29175800
H	0.69163100	2.25162800	-1.62271900
H	3.30148800	2.36654400	-0.02956000
H	1.77921700	2.40062600	2.17393400
H	2.42316300	4.38268400	0.98446600
O	0.15012800	1.87375600	0.98550000
O	1.70032000	0.21844700	0.88834500
O	2.96542900	3.63422700	-1.57011300
H	2.24488200	4.24444800	-1.81251800
O	0.78503700	4.50302700	-0.29332700
H	0.02582700	4.73297200	0.28280000
C	0.09504600	3.68632100	2.49595300
H	0.66679500	4.48596900	2.97367600
H	-0.30346000	3.02794000	3.27690900
O	-0.97670500	4.32131300	1.78342600
H	-1.52538200	3.60760500	1.41414900
C	2.65978300	-0.52735000	0.29400700
C	2.98881800	-0.50275800	-1.01804300
C	3.33075800	-1.46409700	1.26372200
C	3.96699000	-1.46580500	-1.57188300
C	4.72287500	-1.84183700	0.74183900
H	3.41487800	-0.98165300	2.24060900
H	5.37107400	-2.82890800	-1.01602200
S	2.24074300	0.56822700	-2.22243500
O	4.12667100	-1.60135500	-2.78880800
N	4.63767500	-2.22925600	-0.65584200
H	5.38068500	-0.96479900	0.86401100
H	2.70600700	-2.35908800	1.39082900
C	5.37771100	-2.97211200	1.52393300
O	5.91582600	-3.93359200	1.01699400
O	5.32309800	-2.74937300	2.84626700
H	5.78611300	-3.48499900	3.29297800
O	-1.54983600	-0.75939100	-1.92664200
H	-1.27858000	-1.65258300	-1.58857600
O	-2.90117500	-0.69386600	-1.42757500
C	-3.20404400	-1.79848700	-0.69275000
O	-2.38755100	-2.69079000	-0.53359100
C	-4.58213700	-1.76148600	-0.15484900
C	-5.01367800	-2.85839600	0.60324400
C	-5.43715300	-0.67330900	-0.38665700
C	-6.30247600	-2.86833900	1.13025400
H	-4.33656000	-3.68836000	0.77020900
C	-6.71897700	-0.70867200	0.15106800

H	-5.11109500	0.17837600	-0.97069300
C	-7.16545100	-1.79333200	0.90820900
H	-6.64280000	-3.71484000	1.71786400
H	-8.17022000	-1.79606600	1.31638300
Cl	-7.80279200	0.64903800	-0.13134000

VdW adduct-*R*
PCM(H_2O)-B3LYP/6-31g(d),
el. energy = -2476.592778 a.u.

C	-1.74853500	-1.93716900	1.16612100
C	-3.15812800	-1.60385700	0.63355500
C	-3.17240000	-1.46837200	-0.89629100
C	-1.28022900	-2.91972000	-1.12475700
C	-1.19732600	-3.14780500	0.39038200
H	-4.19362500	-1.37179500	-1.27166500
H	-3.81636700	-2.44329500	0.87456900
H	-1.07858700	-1.08283200	1.03154000
H	-0.59938000	-2.09998900	-1.38715500
H	-0.13573200	-3.27746100	0.65390600
O	-2.62341000	-2.57779000	-1.53277500
O	-2.41435200	-0.32486800	-1.36041300
O	-1.76789900	-2.22664000	2.55357800
H	-2.12858000	-3.12948700	2.62441800
O	-1.95587300	-4.27006100	0.83682300
H	-1.96198000	-4.92794900	0.11007700
C	-0.90882600	-4.14277900	-1.95946800
H	0.09897200	-4.47639300	-1.69928200
H	-0.92510600	-3.87918200	-3.02366600
O	-1.78349700	-5.25197200	-1.70635200
H	-2.66932400	-4.98010000	-2.00329400
C	-2.54681900	0.87729400	-0.75237900
C	-3.18626200	1.10933000	0.41656400
C	-1.93161200	1.98958100	-1.55883500
C	-3.38809200	2.49145000	0.90744600
C	-1.59987900	3.17547300	-0.64412600
H	-1.02092800	1.62754800	-2.04189600
H	-2.79763300	4.41178300	0.59085200
S	-3.94508400	-0.14663300	1.41979300
O	-4.14293500	2.73956800	1.85312300
N	-2.73403000	3.47263900	0.21535800
H	-0.71709400	2.89556300	-0.04608900
H	-2.63107300	2.28759000	-2.35219400
C	-1.21788400	4.43816800	-1.40208500
O	-1.65510200	5.54324400	-1.15833700
O	-0.30015600	4.18454900	-2.34848100
H	-0.06938500	5.03280200	-2.77540500
O	0.61143800	0.81909000	0.54097900
H	0.67423800	0.99612500	1.51628400
O	1.99353900	0.54425400	0.23575400
C	2.76597400	0.63674000	1.35391200
O	2.27483000	0.91857700	2.43408500
C	4.19390900	0.36327200	1.08040200
C	5.08617500	0.42852200	2.15927700
C	4.65288100	0.04910700	-0.20784900
C	6.44006600	0.17940400	1.95031600
H	4.71003300	0.67301600	3.14614300
C	6.00969700	-0.19515600	-0.38884500
H	3.97008200	-0.00322100	-1.04678600
C	6.91247000	-0.13440100	0.67436400
H	7.13623200	0.22834100	2.78131200
H	7.96604000	-0.32905700	0.50617900
Cl	6.60029400	-0.59014500	-1.99894900

TS-*S*
PCM(H_2O)-B3LYP/6-31g(d),
el. energy = -2476.580023 a.u.
im. frequency -382.18

C	-2.68041600	2.17744100	1.38355200
C	-1.36390900	1.49005600	0.96813500
C	-1.22686300	1.37293100	-0.55558900
C	-2.61572200	3.29596900	-0.89395700
C	-2.79224600	3.50810500	0.61644200
H	-0.24300200	0.99019200	-0.82946300
H	-0.52688100	2.10218900	1.31202900
H	-3.53624700	1.53998700	1.14144000
H	-3.46583100	2.70778300	-1.26203700
H	-3.79926400	3.92149300	0.78264400
O	-1.38571400	2.59428800	-1.19112100
O	-2.22601100	0.48157500	-1.12935400
O	-2.72834400	2.41088400	2.77811400
H	-2.15134100	3.18129000	2.93433000
O	-1.80525000	4.36008800	1.18991900
H	-1.54339400	5.01033400	0.50393700
C	-2.54154900	4.59174800	-1.69779400
H	-3.44505300	5.18259000	-1.52725100
H	-2.47568700	4.35609400	-2.76633100
O	-1.43591100	5.41308500	-1.29681900
H	-0.62582700	4.92890900	-1.53307200
C	-2.48321000	-0.70652200	-0.55803300
C	-2.05064500	-1.11910900	0.66070500
C	-3.30506100	-1.60898800	-1.43869400
C	-2.27093800	-2.50960400	1.12512600
C	-4.04018800	-2.65034700	-0.58315700
H	-4.02291000	-1.01092100	-2.00497200
H	-3.33737800	-4.19062900	0.69406100
S	-1.10217700	-0.13252500	1.77174400
O	-1.67941000	-2.95535200	2.11243900
N	-3.12040800	-3.25675000	0.36489100
H	-4.86119200	-2.13866100	-0.05424000
H	-2.63801400	-2.09709000	-2.16229800
C	-4.69031700	-3.75569000	-1.40529900
O	-4.59468100	-4.93819300	-1.15495200
O	-5.41544100	-3.25160100	-2.41439600
H	-5.83460200	-3.99787700	-2.88613200
O	1.06688200	-0.05318000	1.03975300
H	1.23175700	-0.99110100	1.32081500
O	2.70981600	0.08025800	0.47930900
C	3.29475300	-1.07075500	0.71116400
O	2.72426500	-2.03535100	1.23469800
C	4.73262700	-1.12907300	0.28615300
C	5.44475100	-2.31686900	0.49290000
C	5.35989100	-0.02440100	-0.30553400

C	6.78179100	-2.40159600	0.10934300
H	4.94113900	-3.15980800	0.95231100
C	6.69522400	-0.13278600	-0.68025500
H	4.81270400	0.89632400	-0.46698400
C	7.41956500	-1.30857500	-0.48147800
H	7.33650600	-3.32118300	0.26856100
H	8.45995100	-1.36783400	-0.78232000
Cl	7.49149500	1.25364500	-1.42589800

TS-*R*
PCM(H_2O)-B3LYP/6-31g(d),
el. energy = -2476.575496 a.u.
im. frequency -389.54

C	1.47373300	-2.02390200	0.21265800
C	2.27504100	-1.40451000	-0.95190100
C	3.60460000	-0.78852600	-0.49327400
C	3.70294000	-2.24527800	1.40335800
C	2.40406100	-2.95861000	1.00605500
H	4.21575700	-0.48457500	-1.34700500
H	2.52656900	-2.20644500	-1.65084000
H	1.07990700	-1.23909000	0.86288600
H	3.45893000	-1.44184500	2.10961200
H	1.88728200	-3.26192700	1.92971200
O	4.36259300	-1.67938100	0.24602200
O	3.40827000	0.38236700	0.34359700
O	0.36110700	-2.75227800	-0.27376600
H	0.72075300	-3.60056900	-0.59471600
O	2.61596900	-4.08488100	0.16015300
H	3.47203200	-4.48892600	0.41716100
C	4.73969200	-3.15845300	2.05293500
H	4.31245400	-3.61680700	2.94843800
H	5.61407600	-2.56684700	2.34773700
O	5.13055900	-4.23617700	1.19099600
H	5.61120800	-3.84108600	0.44330900
C	2.64040800	1.38535500	-0.12129900
C	1.74011700	1.28999600	-1.12835000
C	2.91363200	2.69828700	0.56036200
C	1.07246800	2.49436400	-1.68209700
C	1.69850100	3.62483400	0.41697400
H	3.12953200	2.51914900	1.61652900
H	0.75161300	4.46581700	-1.28427900
S	1.30618600	-0.20596000	-1.96150000
O	0.45126300	2.45212200	-2.74645500
N	1.24397700	3.63987800	-0.96347700
H	0.90379200	3.25038200	1.08284400
H	3.80932400	3.14999800	0.11296000
C	1.97742900	5.05746400	0.85277200
O	1.67261900	6.03804900	0.20805300
O	2.56455700	5.09558100	2.05771600
H	2.68974900	6.03352900	2.30234200
O	-0.71938700	-0.19875800	-1.06380600
H	-0.90279000	-1.15015800	-1.27779900
O	-2.37007800	-0.06281700	-0.33835700
C	-3.07182900	-1.07920400	-0.75705200
O	-2.62899500	-1.99999700	-1.45457900
C	-4.51180300	-1.06079400	-0.30970300
C	-5.34065300	-2.13150400	-0.66533700
C	-5.02360000	0.00152400	0.44686800
C	-6.67630600	-2.14266500	-0.26699200
H	-4.92534100	-2.94329800	-1.25174600
C	-6.35988700	-0.03052400	0.83317800
H	-4.38593400	0.83220600	0.72355600
C	-7.19903600	-1.08994200	0.48758900
H	-7.31994000	-2.97277400	-0.54221000
H	-8.23737800	-1.09147800	0.80119500
Cl	-7.00960500	1.30663900	1.78597700

mCBA
PCM(H_2O)-B3LYP/6-31g(d),
el. energy = -880.424008 a.u.

O	2.29992400	-1.75740800	-0.00000200
C	2.31030500	-0.40773700	0.00037100
O	3.34151100	0.24023600	-0.00005300
C	0.94416700	0.18576600	0.00018800
C	0.82722700	1.58159500	0.00010000
C	-0.20364300	-0.61919200	0.00010400
C	-0.43315500	2.17402500	-0.00005100
H	1.72770300	2.18553200	0.00016100
C	-1.45191800	-0.00484600	-0.00005000
H	-0.12004200	-1.69877600	0.00016700
C	-1.58520400	1.38406900	-0.00012800
H	-0.52719500	3.25541800	-0.00010800
H	-2.57006500	1.83843800	-0.00024700
Cl	-2.90114400	-1.00906900	-0.00014500
H	3.23090600	-2.05115400	-0.00027200

2-*S*
PCM(H_2O)-B3LYP/6-31g(d),
el. energy = -1596.218344 a.u.

C	-1.77066100	0.50063800	1.27645800
C	-1.50138600	1.04227900	-0.13645500
C	-1.34318400	-0.06945400	-1.16801100
C	-2.75033000	-1.53004700	0.10006600
C	-2.96889400	-0.46541200	1.18768200
H	-1.24010200	0.34108000	-2.17542100
H	-2.36401700	1.63863700	-0.44794800
H	-0.89711600	-0.03238200	1.66383800
H	-1.92484900	-2.18001300	0.41661500
H	-3.06768000	-0.98922000	2.15108800
O	-2.42180600	-0.93890300	-1.18090100
O	-0.16183700	-0.88795200	-0.90840100
O	-2.04474300	1.54537800	2.19277900
H	-2.95714500	1.82995900	1.99954000
O	-4.10518200	0.36121100	0.95625800
H	-4.76293500	-0.17202400	0.46114600
C	-3.97504600	-2.40170300	-0.16659600
H	-4.28555100	-2.89289300	0.75898400
H	-3.72158100	-3.17339700	-0.90266200
O	-5.09797300	-1.63036800	-0.61577900
H	-4.86014900	-1.26806000	-1.48681300
C	0.98919100	-0.31410200	-0.54427800
C	1.18035400	0.99726500	-0.24597800

C	2.13296600	-1.29461400	-0.51723700
C	2.53327000	1.53021200	0.03699200
C	3.22617200	-0.80136100	0.44090600
H	1.76771400	-2.27609700	-0.20715600
H	4.43847900	0.93648600	0.44543600
S	-0.10399000	2.24866400	-0.25853000
O	2.74990300	2.74421000	0.09618400
N	3.51925600	0.59832400	0.18421600
H	2.86359300	-0.94068900	1.47287700
H	2.52424500	-1.39329000	-1.53849500
C	4.52098500	-1.59791800	0.33851500
O	5.62379400	-1.09967100	0.26448700
O	4.28812900	-2.91788000	0.38432100
H	5.15033100	-3.37688300	0.34930500
O	-0.23963700	2.84987500	-1.65466900

2-*R*
PCM(H_2O)-B3LYP/6-31g(d),
el. energy = -1596.208204 a.u.

C	-2.21264800	0.90302600	0.97414000
C	-1.53980500	0.98244800	-0.41133100
C	-1.21081100	-0.38441600	-1.01395700
C	-2.85464300	-1.47799400	0.31743800
C	-3.33314800	-0.15960100	0.93248800
H	-0.89815600	-0.29192700	-2.05845000
H	-2.24764000	1.45666800	-1.09694800
H	-1.47837900	0.64303000	1.74163300
H	-2.09171100	-1.91621900	0.97318100
H	-3.65616600	-0.36019500	1.96589400
O	-2.28687400	-1.25461600	-0.99375600
O	-0.13022000	-1.06548400	-0.31069400
O	-2.78199900	2.14629800	1.34464000
H	-3.63750300	2.18048400	0.87679800
O	-4.39218600	0.44147500	0.19059600
H	-4.93046600	-0.28220300	-0.19465700
C	-3.95960500	-2.51129400	0.11089500
H	-4.43941700	-2.73112100	1.06793800
H	-3.52426800	-3.43760900	-0.28160400
O	-4.99304400	-2.03496600	-0.76272900
H	-4.59560100	-1.94072100	-1.64562000
C	1.05564800	-0.44233000	-0.27019200
C	1.24026000	0.88954600	-0.42328000
C	2.21282400	-1.39060800	-0.10659700
C	2.60625300	1.45742600	-0.54687800
C	3.42251000	-0.63939400	0.46518900
H	1.92066700	-2.20763400	0.55776000
H	4.56884800	0.97682000	-0.28764900
S	-0.04826200	2.15413000	-0.44286800
O	2.79790400	2.61663700	-0.92415500
N	3.62910600	0.59715000	-0.26908800
H	3.22291200	-0.43198700	1.52954400
H	2.44626800	-1.83001500	-1.08576400
C	4.71100600	-1.45066600	0.42809800
O	5.77358900	-1.02605800	0.02704800
O	4.52936200	-2.67933500	0.93410700
H	5.38999100	-3.14214600	0.91667400
O	0.04497000	2.85752200	0.90178000

References

(1) Jiménez-Barbero, J.; Dragoni, E.; Venturi, C.; Nannucci, F.; Ardá, A.; Fontanella, M.; André, S.; Cañada, F. J.; Gabius, H.-J.; Nativi, C. α- O -Linked Glycopeptide Mimetics: Synthesis, Conformation Analysis, and Interactions with Viscumin, a Galactoside-Binding Model Lectin. *Chem. - A Eur. J.* **2009**, *15* (40), 10423–10431. https://doi.org/10.1002/chem.200901077.

(2) Amedei, A.; Asadzadeh, F.; Papi, F.; Vannucchi, M. G.; Ferrucci, V.; Bermejo, I. A.; Fragai, M.; De Almeida, C. V.; Cerofolini, L.; Giuntini, S.; Bombaci, M.; Pesce, E.; Niccolai, E.; Natali, F.; Guarini, E.; Gabel, F.; Traini, C.; Catarinicchia, S.; Ricci, F.; Orzalesi, L.; Berti, F.; Corzana, F.; Zollo, M.; Grifantini, R.; Nativi, C. A Structurally Simple Vaccine Candidate Reduces Progression and Dissemination of Triple-Negative Breast Cancer. *iScience* **2020**, *23* (6), 101250. https://doi.org/10.1016/j.isci.2020.101250.

(3) Pesce, E.; Sodini, A.; Palmieri, E.; Valensin, S.; Tinti, C.; Rossi, M.; De Rosa, A.; Fragai,

M.; Papi, F.; Cordiglieri, C.; Berti, F.; Grifantini, R.; Micoli, F.; Nativi, C. GMMA Decorated with Mucin 1 Tn/STn Mimetics Elicit Specific Antibodies Response and Inhibit Tumor Growth. *npj Vaccines* **2025**, *10* (1), 1–14. https://doi.org/10.1038/s41541-025-01127-8.

(4) Schumann, F. H.; Riepl, H.; Maurer, T.; Gronwald, W.; Hans, K. N.; Kalbitzer, R. Combined Chemical Shift Changes and Amino Acid Specific Chemical Shift Mapping of Protein – Protein Interactions. *J Biomol NMR* **2007**, *39*, 275–289. https://doi.org/10.1007/s10858-007-9197-z.

(5) Martínez-Sáez, N.; Peregrina, J. M.; Corzana, F. Principles of Mucin Structure: Implications for the Rational Design of Cancer Vaccines Derived from MUC1-Glycopeptides. *Chem. Soc. Rev.* **2017**, *46* (23), 7154–7175. https://doi.org/10.1039/c6cs00858e.

(6) Schuman, J.; Campbell, A. P.; Koganty, R. R.; Longenecker, B. M. Probing the Conformational and Dynamical Effects of O-Glycosylation within the Immunodominant Region of a MUC1 Peptide Tumor Antigen. *J. Pept. Res.* **2003**, *61* (3), 91–108. https://doi.org/10.1034/j.1399-3011.2003.00031.x.

(7) Kim, J.; Ryu, C.; Ha, J.; Lee, J.; Kim, D.; Ji, M.; Park, C. S.; Lee, J.; Kim, D. K.; Kim, H. H. Structural and Quantitative Characterization of Mucin-Type O -Glycans and the Identification of O -Glycosylation Sites in Bovine Submaxillary Mucin. *Biomolecules* **2020**, *10* (4), 636–650. https://doi.org/doi:10.3390/biom10040636.
